# Composition and diversity of the subgingival microbiome and its relationship with age in postmenopausal women: an epidemiologic investigation

**DOI:** 10.1186/s12903-019-0906-2

**Published:** 2019-11-13

**Authors:** Michael J. LaMonte, Robert J. Genco, Michael J. Buck, Daniel I. McSkimming, Lu Li, Kathleen M. Hovey, Christopher A. Andrews, Wei Zheng, Yijun Sun, Amy E. Millen, Maria Tsompana, Hailey R. Banack, Jean Wactawski-Wende

**Affiliations:** 1 Department of Epidemiology and Environmental Health, School of Public Health and Health Professions, University at Buffalo, 270 Farber Hall, 3435 Main Street, Buffalo, NY 14214 USA; 2 Department of Oral Biology, School of Dental Medicine, UB Microbiome Center, University at Buffalo, Buffalo, NY USA; 3 Department of Biochemistry, School of Medicine and Biomedical Sciences, NY State Center of Excellence in Bioinformatics and Life Sciences, University at Buffalo, Buffalo, NY USA; 4 Genome, Environment, and Microbiome Center of Excellence, University at Buffalo, Buffalo, NY USA; 5 Department of Microbiology and Immunology and Department of Computer and Engineering Science, NY State Center of Excellence in Bioinformatics and Life Sciences, University at Buffalo, Buffalo, NY USA; 6 Department of Ophthalmology, School of Medicine, University of Michigan, Ann Arbor, MI USA

**Keywords:** Aging, Women, Oral Microbiome, Epidemiology

## Abstract

**Background:**

The extent to which the composition and diversity of the oral microbiome varies with age is not clearly understood.

**Methods:**

The 16S rRNA gene of subgingival plaque in 1219 women, aged 53–81 years, was sequenced and its taxonomy annotated against the Human Oral Microbiome Database (v.14.5). Composition of the subgingival microbiome was described in terms of centered log(2)-ratio (CLR) transformed OTU values, relative abundance, and prevalence. Correlations between microbiota abundance and age were evelauted using Pearson Product Moment correlations. *P*-values were corrected for multiple testing using the Bonferroni method.

**Results:**

Of the 267 species identified overall, *Veillonella dispar* was the most abundant bacteria when described by CLR OTU (mean 8.3) or relative abundance (mean 8.9%); whereas *Streptococcus oralis, Veillonella dispar and Veillonella parvula* were most prevalent (100%, all) when described as being present at any amount. Linear correlations between age and several CLR OTUs (Pearson *r* = − 0.18 to 0.18), of which 82 (31%) achieved statistical significance (*P* < 0.05). The correlations lost significance following Bonferroni correction. Twelve species that differed across age groups (each corrected *P* < 0.05); 5 (42%) were higher in women ages 50–59 compared to ≥70 (corrected *P* < 0.05), and 7 (48%) were higher in women 70 years and older.

**Conclusions:**

We identified associations between several bacterial species and age across the age range of postmenopausal women studied. Understanding the functions of these bacteria could identify intervention targets to enhance oral health in later life.

## Background

The availability of high throughput metagenomics sequencing technology has allowed for deeper understanding of complex microbiota ecologies and their aggregate functional capacities within a defined microbiome [1, 2]. Marked differences in composition and function of microbiomes have been shown between various body sites among individuals [3, 4]. It has become increasingly clear that the microbiota and microbiome are correlated with both health and disease states in humans [5], and that the aging process could be an important determinant of these relationships [6, 7]. Aging is a complex, multifactorial process characterized by progressively lower resilience to stress, increased homeostatic imbalance, and greater susceptibility to pathologic insult and disease onset [8]. Changes in microbiome diversity and function have been observed with increasing age [9]. Alterations in the host environment that occur with physiologic aging processes could enable untoward shifts in relative abundance of commensal and pathogenic bacteria, and enhanced expression of pathogen genomes which, in turn, could heighten disease susceptibility. In support of this hypothesis are studies demonstrating links between human microbiomes and several diseases of aging including obesity, diabetes, heart disease, and certain cancers [5, 7, 10].

The oral microbiota comprise one of the most complex and diverse human microbiomes [3, 11, 12]. Oral bacteria have important functional roles that contribute to maintenance of oral health [13], to oral diseases such as caries and periodontitis in the setting of dysbiosis [14, 15], and potentially to systemic diseases of aging by way of bacterial translocation through ulcerated oral epithelium, aspiration, or ingestion [7, 16]. This could have important implications to public health given the rapid growth in numbers of older adults expected in coming decades.

Surprisingly, there exists a limited understanding of oral microbiota in aging populations. Feres et al. [17] conducted a comprehensive review of published literature and concluded that the majority of oral microbiome studies have included younger and middle-aged adults. Only a small number of studies have described the microbiome in older adults, among which sample sizes of adults 60 years and older tended to be, on average, modest (e.g., < 200), the majority of whom were men and were selected to have moderate to severe periodontitis [17–19]. A majority of previous studies have used low throughput microbial measurement techniques, such as microbial culture and targeted DNA probes, which result in an incomplete characterization of the oral microbiome composition and diversity in relationship to groups of men and women of differing ages. Recent investigations have extended these previous studies by using next generation sequencing methods, but again relatively small sample sizes (< 100) limited the contrasts that could be performed in relation to age in the majority of these studies [20–23].

Thus, at present, an incomplete understanding of the composition and characteristics of the oral microbiome exists in the context of aging, particularly in women. A critical step in advancing knowledge on how the oral microbiome relates with the frequency of oral (e.g., periodontitis) or systemic (e.g., breast cancer) diseases of aging, is to first understand the extent of the composition and how the microbiota vary with host characteristics, such as age. This information will be important in later understanding the interplay of the microbiome with pathogenic changes over time. Application of epidemiologic study methods to study populations *not* selected on disease status is a suggested approach to establish a foundational understanding of microbiome diversity expected in a population that then allow for hypotheses pertaining to disease-related variation that can then be accurately evaluated [24]. The objective of this current cross-sectional investigation was to describe the composition and diversity of the subgingival plaque microbiome and its relationship with age in a cohort of ambulatory postmenopausal women, aged 53–81 years, who were enrolled in an ongoing study from the community dwelling women without selection on periodontal health status at enrollment.

## Methods

### Participants

**Fig. 1:**
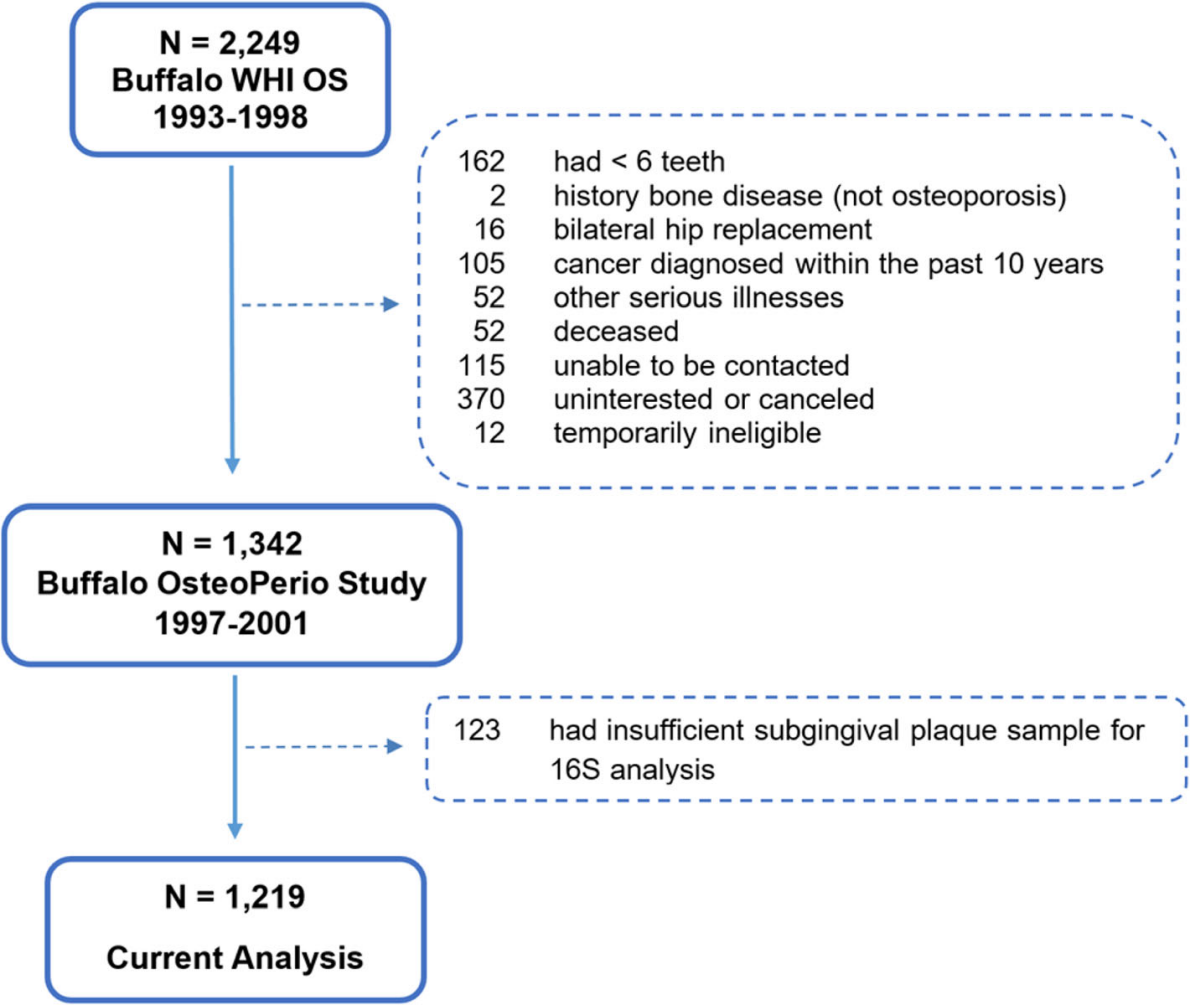
Flow of participants into the Buffalo OsteoPerio Study

The present study included 1219 postmenopausal women enrolled in the Buffalo Osteoporosis and Periodontitis (OsteoPerio) Study, which is an ancillary study conducted at the Buffalo (NY) clinical center of the Women’s Health Initiative Observational Study (WHI OS). Participants provided written informed consent for all components of the studies, which were conducted in accord with the Helsinki Declaration on human subjects research. Experimental protocols for all aspects of the WHI study, the OsteoPerio Study, and the microbiome study detailed in this paper were approved by the Institutional Review Board at the University at Buffalo. This manuscript conforms to the STROBE guidelines for human observational studies. Details about recruitment, enrollment criteria, study implementation and measurements have been published for the WHI OS [25] and the OsteoPerio study [26, 27]. Briefly, 2249 postmenopausal women, ages 50–79, enrolled into the WHI OS at the Buffalo center between 1994 and 1998. Of these, 1362 enrolled into the OsteoPerio study 3 years later in 1997–2001 (mean age 66; range 53–81 years). Enrollment into the OsteoPerio study required at least 6 teeth present and no history of bone disease other than osteoporosis and no history of cancer in the previous 10 years. Women completed standardized questionnaires pertaining to demographic information, lifestyle habits, and personal health history, as well as undertaking a whole mouth oral examination conducted by trained and calibrated examiners. Neighborhood socioeconomic status was derived from questionnaire responses and census tract information [28]. Detailed descriptions of the oral examination measures and their reproducibility have been published [26]. Figure 1 shows a flow chart of participant enrollment into the OsteoPerio study.

### Subgingival plaque samples

A protocol for obtaining subgingival plaque samples was developed for this study and has been published [29]. Fine paper points – (#504; Henry Schein, Melville, NY) were placed in the gingival pockets of up to 12 pre-specified teeth (6 maxillary and 6 mandibular arch teeth) for 10 S*. index* teeth [3, 5, 7, 9, 12, 14, 19, 21, 23, 25, 28, 30, and] were usually sampled. Alternative teeth [2, 4, 8, 10, 13, 15, 18, 20, 24, 26, 29, 31, and] were used if the corresponding index tooth was missing. Paper points containing all subgingival plaque samples from each arch were placed directly into 4 mL lactated Ringer’s solution. The solution was taken to the lab where it was vortexed for dispersion of microorganisms, placed in cryogenic straws, frozen immediately at -80 °C and later placed in cryogenic tanks at -196 °C as previously described [29]. Before next generation sequencing, samples were placed in − 80 freezers and later thawed, with upper and lower arch samples combined into a single aliquot for the purpose of sequencing.

### DNA isolation and purification

Genomic DNA was isolated using the QIAsymphony SP automated system (Qiagen, Valencia, CA) with the QIAsymphony DSP Virus/Pathogen Mini Kit (Qiagen, Valencia, CA) and the Complex200_V6_DSP protocol after enzymatic pretreatment. In detail, 500 μl of oral plaque solution contained in a barcoded 2 ml tube was equilibrated at room temperature (15-25 °C). Bacteria was pelleted by centrifugation at 5000×g for 10 min, resuspended in a 300 μl lysis solution (20 mg/ml lysozyme in 20 mM Tris-HCl, pH 8.0; 2 mM EDTA; 1.2% Triton X-100) and incubated at 37 °C for 30 min. Following incubation, tubes were briefly centrifuged to remove drops from inside the lid and then placed in the tube carrier of the QIAsymphony SP.

DNA extraction and purification was done according to the Qiasymphony DSP Virus /Pathogen Kit Instructions. Carrier RNA-AVE mixture was added to all samples for increased recovery of nucleic acids. After DNA purification, samples were eluted in a barcoded 96 well elution plate (Qiagen, Valencia, CA). All batches of samples were performed with DNA extraction negative controls and positive controls from a single large pool of mixed plaque samples.

### 16S rRNA amplification and sequencing

Metagenomic amplification of the extracted DNA for 16S amplification of the V3–V4 hypervariable region proceeded following the Illumina manufacturer protocol (Illumina Inc., San Diego, CA) with modifications developed for our study [30]. The Illumina protocol relies on *limited* cycle PCR for addition of Illumina sequencing adapters and dual-index barcodes to the 16S rRNA V3-V4 ampli. We also included as part of the 96-well plates, samples of the UltraClean DNA free PCR water (MO BIO Laboratories, Carlsbad, CA) and RNase/DNase free water (Ambion, Foster City, CA) as negative controls, and genomic DNA from microbial community HM-277D (microbial community B; BEI Resources; Manassas, VA) as a positive control during the amplification process. Metagenomic DNA was amplified using the 16S V3 (341F) forward and V4 (805R) reverse primer pairs with added Illumina adapter overhang nucleotide sequences. Amplicon PCR was completed with 42 μl of genomic DNA, 4 μl of amplicon PCR forward primer (5 μM), 4 μl of amplicon PCR reverse primer (5 μM), and 50 μl of 2x KAPA HiFi HotStart Ready Mix (KapaBiosystems) at 95 °C initial denaturation for 3 min, followed by 25 cycles of 95 °C for 30 s, 62.3 °C for 30 s, and 72 °C for 30 s, and a final extension at 72 °C for 5 min. Reactions were cleaned with Agencourt AMPure XP beads (Beckman Coulter Genomics, South Plainfield, NJ) according to the manufacturer’ s protocol.

Library generation was performed using 5 μl of amplicon PCR product DNA, 5 μl of Illumina Nextera XT Index Primer 1 (N7xx), 5 μl of Nextera XTIndex Primer 2 (S5xx), 25 μ l of 2x KAPA HiFi HotStart Ready Mix, and 10 μl of PCR-grade water (UltraClean MO BIO Laboratories, Inc.), with thermocycling at 95 °C for 3 min, followed by 8 cycles of 95 °C for 30 s, 55 °C for 30 s, and 72 °C for 30 s, and a final extension at 72 °C for 5 min. 16S metagenomic libraries were purified with Agencourt AMPure XP beads and quantified with Quant-iT PicoGreen. Nextera index primer sets (A, B, and C) were rotated for each batch to reduce sequence carryover between MiSeq runs.

Library quality control was performed with the Fragment Analyzer (Advanced Analytical Technologies, Inc., Ankeny, IA) to ascertain average size distribution. Generated 16S rRNA V3-V4 libraries were further quality-controlled using the following internal study criteria: 1. Library concentration of all negative(s) is < 5 ng/μl, 2. Participant samples have a fragment peak distribution with average size of ~ 600 bp, and 3. Negative controls yield a straight line when run in the Fragment Analyzer. If the above cutoffs were met, libraries were normalized and pooled to 4 nM based on PicoGreen concentrations. The pool of normalized libraries were then quantified with the NEBNext Library Quant Kit (New England Biolabs, Inc., Ipswich, M.), denatured with NaOH and diluted to a final concentration of 10 pM with a 20% PhiX (Illumina, Inc., San Diego, CA). 2 × 300 bp paired-end sequencing is performed in the Illumina MiSeq System (Illumina Inc., San Diego, CA) by multiplexing 96 samples per sequencing run with the MiSeq Reagent Kit.

Joining of Illumina paired-end reads were completed using Paired-End reAd mergeR (PEAR version 0.9.6). The percentage of successfully joined pair-end defined the “merge rate”; paired-end reads that could not be joined were removed from downstream analyses. Sequence quality filtering was done with the Fastx-Toolkit (V.0.013) to isolate reads with 90% of their bases having a score higher than Q30, which defined the “pass rate”; reads not meeting this criterion were removed. Primer sequences were trimmed based on the length of the forward and reverse sequencing primers. Following quality-filtering, reads were deduplicated by recording the number and type of identical sequences to reduce downstream processing time.

Taxonomy annotation was done with BLAST [31] at a 97% similarity, for species-level assignment approximation, against bacterial sequences from the HOMD version 14.5. Input query reads were given the same taxonomic label as the best hit in the reference sequence collection, defining the “hit count”; reads with no hits were excluded from downstream analyses. Sequences with the same labels were clustered into one OTU and the raw OTU table was constructed by combining absolute sequence abundances from the deduplication step, generated taxonomy annotations and manually generated metadata. We subsequently filtered the raw OTU table by discarding OTUs with a frequency < 0.02% of the total read count. At the preprocessing sequence analysis step we require a ‘Merge Rate’ ≥ 90%, ‘Pass Rate’ ≥ 60%, and ‘Hit Count’ per sample ≥ 3000.

### Statistical analysis

For this analysis we used several approaches to characterize the composition and diversity of the subgingival microbiome and their relationships with age. Individual OTU counts were normalized using the centered log(2)-ratio (CLR) transformation. Gloor et al. [32] recommends the CLR transformation to account for the complex compositional data structure, to reduce the likelihood of spurious correlations, and to enhance the meaningfulness of subcomposition comparisons. A positive CLR OTU value for given taxon indicates a relatively higher amount than the overall composition mean, which is 0; a negative value indicates relatively lower amount. The fold-difference for a reported CLR OTU value relative to the compositional mean, can be determined by raising 2 to the power of the base 2 logarithm. For example, a mean CLR of 3, reflects an 8-fold (2^3^) higher abundance compared to the compositional mean; a mean CLR of − 3 reflects an 8-fold lower abundance. The CLR distribution of each OTU was approximately normal and the variances in groups were similar by visual inspection. Alpha diversity was used to assess species richness and evenness across age categories. The rarefaction curve, bias-corrected Chao1 (richness), OTU count (richness), and Shannon entropy (evenness) values were calculated for each sample using scikit- bio v0.5.5. Beta diversity was evaluated using principal component analysis (PCA) [33]. T-tests were used to evaluate differences in alpha diversity, and PERMANOVA was used to evaluate differences in beta diversity, using SciPy v1.3.0. Comparisons of microbiota between age categories was performed using analysis of variance and evaluation of linear relationships between microbiota and age performed using Pearson product-moment correlations. We nominally defined correlations of |r| < 0.10 as weak, 0.10–0.49 as moderate, 0.50–0.70 as strong, and > 0.70 as very strong. We report uncorrected *p*-values and indicate which are statistically significant after Bonferroni correction for multiple testing.

To provide additional perspective and comparability with previous studies, we also describe microbiome composition and diversity according to conventional measures of relative abundance (the amount of a specific taxon relative to the total composition of the sample in which it is measured) and prevalence (presence of a taxon regardless of relative composition). To minimize the total number of hypothesis tests performed, formal comparisons using these measures were not conducted and these data are presented for descriptive purposes only.

## Results

### Characteristics of study group

Participant characteristics are shown for descriptive purposes in Table 1. Women in the present study were, on average, 66 years of age and the vast majority (97%) were Caucasian. Prevalence of current smoking (3%) and diabetes history (5.2%) was modest, and about half the group reported current use of hormone therapy. The group retained the majority of their natural teeth (mean, 23), the frequency of reported teeth brushing two or more times per day was high (77%) as was frequency of dental visits one or more times per year (91%). Mean pocket depth was 2.2 mm (range 1.2–3.8). As expected, prevalence of current smoking and current hormone therapy use declined with increasing age, and, prevalence of diabetes history was highest among the oldest women. The number of teeth present and frequency of dental visits declined with increasing age and, frequency of teeth brushing was higher in older than younger women. Both neighborhood socioeconomic status and mean pocket depth were similar across age groups.

**Table 1:** Baseline characteristics of OsteoPerio Microbiome Study participants for the overall cohort and by age groups

Characteristic	Overall (*N* = 1219)	50–59 (*N* = 239)	60–69 (*N* = 554)	≥70 (*N* = 426)
Age (years), mean (SD)	66.2 (7.0)	56.7 (1.8)	64.2 (2.9)	74.1 (3.3)
Race-ethnicity: White, *N* (%)	1187 (97.4)	233 (97.5)	537 (96.9)	417 (97.9)
Neighborhood SES, mean (SD)	76.2 (6.9)	75.7 (7.5)	76.6 (6.8)	75.9 (6.7)
Smoking, *N* (%)				
Never	642 (52.7)	117 (48.9)	280 (50.5)	245 (57.6)
Former	537 (44.1)	107 (44.8)	257 (46.4)	173 (40.7)
Current	39 (3.2)	15 (6.3)	17 (3.1)	7 (1.7)
History of treated diabetes, *N* (%)	63 (5.2)	9 (3.8)	26 (4.7)	28 (6.6)
History of treated hypertension, *N* (%)	392 (32.2)	63 (26.4)	148 (26.7)	181 (42.5)
History of treated high cholesterol, *N* (%)	201 (16.5)	26 (10.9)	76 (13.7)	99 (23.4)
Hormone therapy use, *N* (%)				
Never	390 (32.0)	53 (22.2)	158 (28.5)	179 (42.0)
Former E-Alone	132 (10.9)	12 (5.0)	50 (9.0)	70 (16.5)
Former E + P	111 (9.1)	24 (10.1)	58 (10.5)	29 (6.8)
Current E-Alone	307 (25.2)	66 (27.7)	136 (24.6)	105 (24.7)
Current E + P	277 (22.8)	83 (34.9)	152 (27.4)	42 (9.9)
Years taking hormone therapy^a^	5.6 (7.3)	4.6 (4.5)	6.2 (7.0)	5.5 (8.8)
Number of teeth present, mean (SD)	23.2 (5.3)	24.8 (4.0)	23.6 (5.2)	21.9 (5.8)
Brush teeth ≥2 times/day, *N* (%)	942 (77.3)	178 (74.5)	422 (76.2)	342 (80.3)
Floss teeth daily, *N* (%)	529 (43.6)	90 (37.7)	247 (44.8)	192 (45.4)
Dental visit ≥1 time/year, *N* (%)	1114 (91.4)	225 (94.1)	504 (91.0)	385 (90.4)
Mean Pocket Depth (mm), mean (SD)	2.2 (0.4)	2.2 (0.4)	2.2 (0.4)	2.1 (0.4)
Gingival Bleeding (%), mean (SD)	34.4 (23.2)	33.0 (23.5)	34.5 (22.9)	35.1 (23.4)

*SES* socioeconomic statusn. See methods section for its definition and derivation, *E* estrogen, *P* progesti

^a^Never users coded as 0 years

### Microbial community structure and composition

**Fig. 2:**
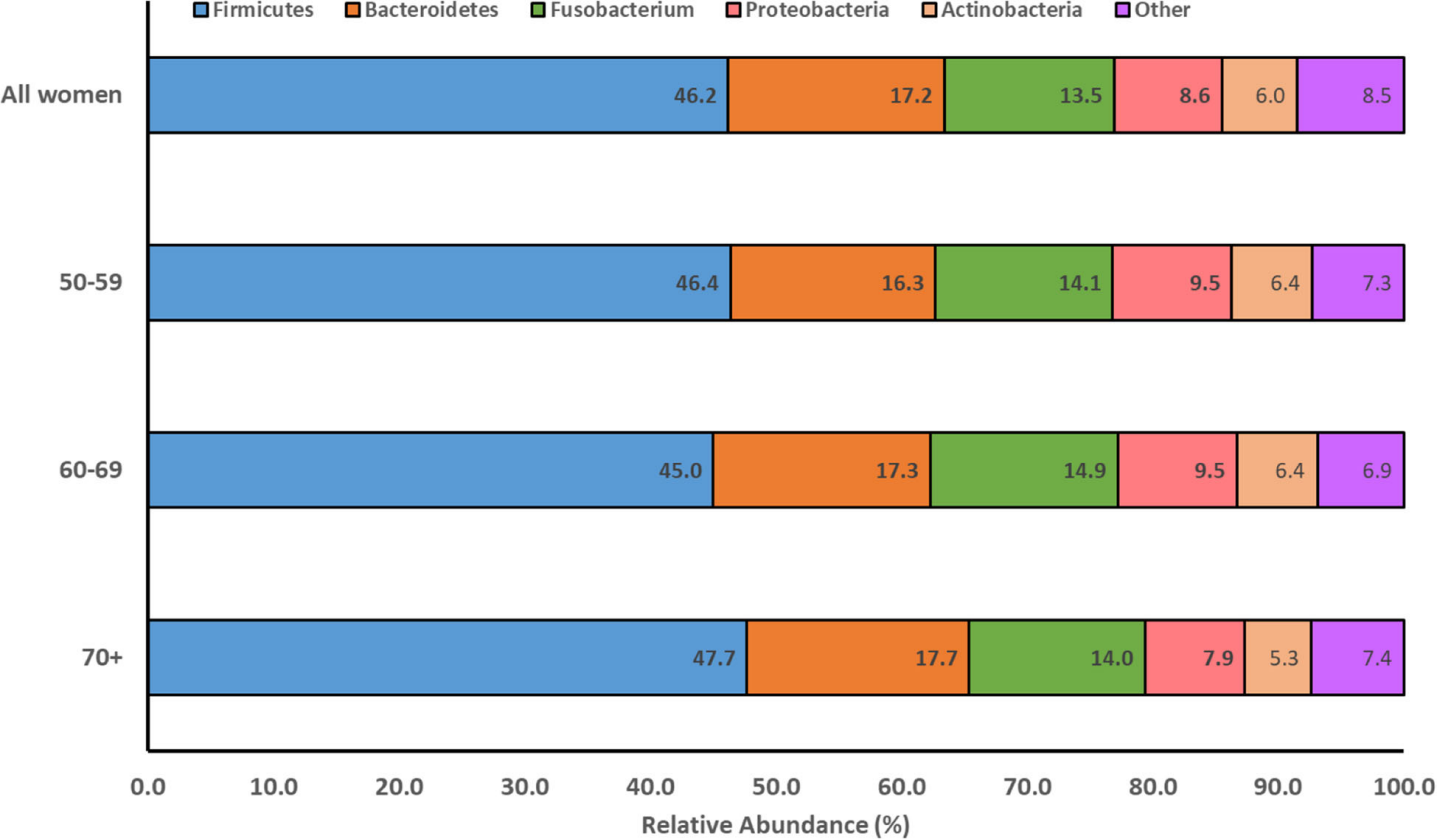
Distribution of phyla among the total reads identified, overall and according to categories of age. Numbers on chart are mean relative abundance. The “other” phylum category comprises 5 phyla ranging in frequency from 0.02 to 3.9%

After filtering out OTUs < 0.02%, the total number of sequence reads for the overall cohort of 1219 women was 120,388,085 (mean reads per sample, 98,760; range 3034 to 1,080,317). Sequence reads per sample was somewhat higher with increasing age, with means (SDs) of 89,442 (71,698), 97,794 (86,908), and 105,243 (80,183) reads in women ages 50–59, 60–69, and ≥ 70 years, respectively. There were 267 microbial taxa identified in the subgingival plaque samples after filtering at 0.02%. The taxonomic classification and mean reads for each taxon overall and by age groups, are presented in Table 2. Of the 120,388,085 read, 46.2% were of the phylum *Firmicutes*, 17.2% *Bacteroidetes*, 13.5% *Fusobacterium,* 8.6% *Proteobacteria*, 6.0% *Actinobacteria*, and the remaining were among other phyla of < 4%, each (Fig. 2). The distribution of phyla was consistent across age groups. At the genus level, the highest mean relative abundance was for *Veillonella* (16.7%), followed by *Streptococcus* (14.2%), *Fusobacterium* (10.7%), *Prevotella* (8.6%), and *Selenomonas* (7.7%); relative abundance of the remaining genera was < 4%, each. This pattern was consistent across age groups. At the species level, among all women, the highest number of mean reads was for *Veillonella dispar* (*Firmicutes phylum;* mean, 8136) and *Veillonella parvula* (*Firmicutes phylum*; mean, 6262) (Table 2). Mean reads for each taxon increased across incremental age groups.

**Table 2:** Taxonomic classification and mean reads for the 267 bacteria identified, overall and by age groups

Phyla	Class	Genus	Species		Age Categories (years)		
				Overall	50–59	60–69	≥70
p__Actinobacteria	c__Actinobacteria	g__Actinobaculum	s__sp._oral_taxon_183	89.8	95.0	88.9	88.0
p__Actinobacteria	c__Actinobacteria	g__Actinobaculum	s__sp._oral_taxon_848	48.2	37.3	52.9	48.2
p__Actinobacteria	c__Actinobacteria	g__Actinomyces	s__gerencseriae	68.6	60.5	66.7	75.6
p__Actinobacteria	c__Actinobacteria	g__Actinomyces	s__israelii	25.1	19.5	24.1	29.5
p__Actinobacteria	c__Actinobacteria	g__Actinomyces	s__johnsonii	106.0	104.9	105.2	107.6
p__Actinobacteria	c__Actinobacteria	g__Actinomyces	s__massiliensis	112.1	134.2	121.5	87.4
p__Actinobacteria	c__Actinobacteria	g__Actinomyces	s__meyeri	59.3	64.8	61.6	53.3
p__Actinobacteria	c__Actinobacteria	g__Actinomyces	s__naeslundii	453.1	482.6	447.6	443.6
p__Actinobacteria	c__Actinobacteria	g__Actinomyces	s__oris	225.7	236.6	218.8	228.5
p__Actinobacteria	c__Actinobacteria	g__Actinomyces	s__sp._oral_taxon_169	195.8	251.2	183.7	180.4
p__Actinobacteria	c__Actinobacteria	g__Actinomyces	s__sp._oral_taxon_170	60.4	44.8	59.8	70.0
p__Actinobacteria	c__Actinobacteria	g__Actinomyces	s__sp._oral_taxon_171	88.2	86.5	89.2	87.9
p__Actinobacteria	c__Actinobacteria	g__Actinomyces	s__sp._oral_taxon_178	27.0	23.9	26.8	29.0
p__Actinobacteria	c__Actinobacteria	g__Actinomyces	s__sp._oral_taxon_180	119.0	121.7	123.6	111.5
p__Actinobacteria	c__Actinobacteria	g__Bifidobacterium	s__dentium	88.5	83.0	75.8	108.2
p__Actinobacteria	c__Actinobacteria	g__Corynebacterium	s__durum	121.3	153.3	120.2	104.7
p__Actinobacteria	c__Actinobacteria	g__Corynebacterium	s__matruchotii	1107	1095	1178	1020
p__Actinobacteria	c__Actinobacteria	g__Microbacterium	s__flavescens	1.5	1.8	1.6	1.1
p__Actinobacteria	c__Actinobacteria	g__Rothia	s__aeria	323.3	311.6	378.9	257.6
p__Actinobacteria	c__Actinobacteria	g__Rothia	s__dentocariosa	975.6	915.0	1039	927.8
p__Actinobacteria	c__Actinobacteria	g__Rothia	s__mucilaginosa	185.3	125.4	179.1	226.9
p__Actinobacteria	c__Actinobacteria	g__Scardovia	s__wiggsiae	68.7	74.1	69.7	64.4
p__Actinobacteria	c__Coriobacteriia	g__Atopobium	s__parvulum	77.9	57.2	78.3	88.8
p__Actinobacteria	c__Coriobacteriia	g__Atopobium	s__rimae	200.0	107.8	200.0	251.7
p__Actinobacteria	c__Coriobacteriia	g__Atopobium	s__sp._oral_taxon_199	47.4	39.1	61.2	34.0
p__Actinobacteria	c__Coriobacteriia	g__Atopobium	s__sp._oral_taxon_416	25.4	1.1	14.6	53.1
p__Actinobacteria	c__Coriobacteriia	g__Olsenella	s__sp._oral_taxon_807	46.9	38.6	44.5	54.7
p__Bacteroidetes	c__Bacteroidetes_[C-1]	g__Bacteroidetes_[G-5]	s__sp._oral_taxon_511	137.4	87.8	155.7	141.4
p__Bacteroidetes	c__Bacteroidia	g__Alloprevotella	s__rava	82.4	52.8	87.6	92.3
p__Bacteroidetes	c__Bacteroidia	g__Alloprevotella	s__sp._oral_taxon_308	28.6	21.3	24.9	37.4
p__Bacteroidetes	c__Bacteroidia	g__Alloprevotella	s__sp._oral_taxon_473	77.5	63.0	82.6	79.1
p__Bacteroidetes	c__Bacteroidia	g__Alloprevotella	s__tannerae	1562	1427	1580	1615
p__Bacteroidetes	c__Bacteroidia	g__Bacteroidaceae_[G-1]	s__sp._oral_taxon_272	39.5	24.6	28.6	62.2
p__Bacteroidetes	c__Bacteroidia	g__Bacteroidales_[G-2]	s__sp._oral_taxon_274	885.8	548.7	935.5	1010
p__Bacteroidetes	c__Bacteroidia	g__Porphyromonas	s__catoniae	104.1	111.4	115.8	84.7
p__Bacteroidetes	c__Bacteroidia	g__Porphyromonas	s__endodontalis	602.5	560.5	657.1	555.0
p__Bacteroidetes	c__Bacteroidia	g__Porphyromonas	s__gingivalis	1055	781.4	752.8	1603
p__Bacteroidetes	c__Bacteroidia	g__Porphyromonas	s__sp._oral_taxon_275	46.0	29.0	62.6	33.9
p__Bacteroidetes	c__Bacteroidia	g__Porphyromonas	s__sp._oral_taxon_278	39.2	22.1	43.1	43.6
p__Bacteroidetes	c__Bacteroidia	g__Porphyromonas	s__sp._oral_taxon_279	310.5	255.9	315.5	334.7
p__Bacteroidetes	c__Bacteroidia	g__Porphyromonas	s__sp._oral_taxon_284	183.2	187.5	187.8	174.9
p__Bacteroidetes	c__Bacteroidia	g__Prevotella	s__baroniae	50.3	29.2	60.5	48.8
p__Bacteroidetes	c__Bacteroidia	g__Prevotella	s__buccae	53.1	17.3	63.0	60.4
p__Bacteroidetes	c__Bacteroidia	g__Prevotella	s__dentalis	102.3	63.3	120.3	100.8
p__Bacteroidetes	c__Bacteroidia	g__Prevotella	s__denticola	773.5	501.4	724.4	989.9
p__Bacteroidetes	c__Bacteroidia	g__Prevotella	s__histicola	100.3	48.8	72.7	165.3
p__Bacteroidetes	c__Bacteroidia	g__Prevotella	s__intermedia	671.5	613.0	766.5	580.8
p__Bacteroidetes	c__Bacteroidia	g__Prevotella	s__loescheii	119.6	131.7	138.7	87.8
p__Bacteroidetes	c__Bacteroidia	g__Prevotella	s__maculosa	185.9	139.8	180.0	219.4
p__Bacteroidetes	c__Bacteroidia	g__Prevotella	s__melaninogenica	339.1	236.0	321.8	419.4
p__Bacteroidetes	c__Bacteroidia	g__Prevotella	s__micans	42.7	37.6	37.1	52.9
p__Bacteroidetes	c__Bacteroidia	g__Prevotella	s__multiformis	46.7	9.2	36.1	81.6
p__Bacteroidetes	c__Bacteroidia	g__Prevotella	s__nigrescens	1997	1719	1960	2200
p__Bacteroidetes	c__Bacteroidia	g__Prevotella	s__oralis	174.4	78.8	158.9	248.2
p__Bacteroidetes	c__Bacteroidia	g__Prevotella	s__oris	1968	1700	2093	1956
p__Bacteroidetes	c__Bacteroidia	g__Prevotella	s__oulorum	211.7	216.2	182.8	246.8
p__Bacteroidetes	c__Bacteroidia	g__Prevotella	s__pallens	94.4	91.5	71.4	125.9
p__Bacteroidetes	c__Bacteroidia	g__Prevotella	s__pleuritidis	579.6	478.7	606.4	601.4
p__Bacteroidetes	c__Bacteroidia	g__Prevotella	s__saccharolytica	73.7	52.8	80.9	76.1
p__Bacteroidetes	c__Bacteroidia	g__Prevotella	s__salivae	120.5	94.7	117.3	139.1
p__Bacteroidetes	c__Bacteroidia	g__Prevotella	s__sp._oral_taxon_292	69.4	50.1	57.5	95.7
p__Bacteroidetes	c__Bacteroidia	g__Prevotella	s__sp._oral_taxon_300	269.5	256.3	243.5	310.7
p__Bacteroidetes	c__Bacteroidia	g__Prevotella	s__sp._oral_taxon_306	41.1	17.2	38.9	57.5
p__Bacteroidetes	c__Bacteroidia	g__Prevotella	s__sp._oral_taxon_313	70.5	86.9	44.0	95.8
p__Bacteroidetes	c__Bacteroidia	g__Prevotella	s__sp._oral_taxon_314	65.2	59.2	52.7	85.0
p__Bacteroidetes	c__Bacteroidia	g__Prevotella	s__sp._oral_taxon_317	832.0	631.4	832.4	944.0
p__Bacteroidetes	c__Bacteroidia	g__Prevotella	s__sp._oral_taxon_376	44.8	45.1	43.6	46.1
p__Bacteroidetes	c__Bacteroidia	g__Prevotella	s__sp._oral_taxon_472	250.3	270.4	279.0	201.9
p__Bacteroidetes	c__Bacteroidia	g__Prevotella	s__sp._oral_taxon_475	21.8	19.1	17.4	28.9
p__Bacteroidetes	c__Bacteroidia	g__Prevotella	s__sp._oral_taxon_526	55.1	21.5	70.7	53.7
p__Bacteroidetes	c__Bacteroidia	g__Prevotella	s__veroralis	113.8	84.1	160.3	70.0
p__Bacteroidetes	c__Bacteroidia	g__Tannerella	s__forsythia	577.6	374.7	542.0	737.8
p__Bacteroidetes	c__Bacteroidia	g__Tannerella	s__sp._oral_taxon_286	93.0	68.6	100.9	96.6
p__Bacteroidetes	c__Bacteroidia	g__Tannerella	s__sp._oral_taxon_808	35.6	25.4	32.6	45.3
p__Bacteroidetes	c__Flavobacteriia	g__Bergeyella	s__sp._oral_taxon_322	164.1	194.8	173.9	134.1
p__Bacteroidetes	c__Flavobacteriia	g__Bergeyella	s__sp._oral_taxon_907	34.8	34.7	36.7	32.4
p__Bacteroidetes	c__Flavobacteriia	g__Capnocytophaga	s__gingivalis	502.9	619.2	471.7	478.3
p__Bacteroidetes	c__Flavobacteriia	g__Capnocytophaga	s__granulosa	597.4	513.3	629.6	602.6
p__Bacteroidetes	c__Flavobacteriia	g__Capnocytophaga	s__leadbetteri	614.5	534.6	619.4	653.0
p__Bacteroidetes	c__Flavobacteriia	g__Capnocytophaga	s__sp._oral_taxon_323	37.7	26.7	40.2	40.5
p__Bacteroidetes	c__Flavobacteriia	g__Capnocytophaga	s__sp._oral_taxon_324	33.8	19.5	32.3	43.8
p__Bacteroidetes	c__Flavobacteriia	g__Capnocytophaga	s__sp._oral_taxon_326	258.0	222.7	281.8	246.9
p__Bacteroidetes	c__Flavobacteriia	g__Capnocytophaga	s__sp._oral_taxon_332	64.5	104.3	60.6	47.3
p__Bacteroidetes	c__Flavobacteriia	g__Capnocytophaga	s__sp._oral_taxon_336	159.5	127.8	157.8	179.6
p__Bacteroidetes	c__Flavobacteriia	g__Capnocytophaga	s__sp._oral_taxon_338	62.3	62.5	56.7	69.5
p__Bacteroidetes	c__Flavobacteriia	g__Capnocytophaga	s__sp._oral_taxon_380	29.6	23.3	40.3	19.1
p__Bacteroidetes	c__Flavobacteriia	g__Capnocytophaga	s__sp._oral_taxon_412	43.7	42.0	46.4	41.2
p__Bacteroidetes	c__Flavobacteriia	g__Capnocytophaga	s__sp._oral_taxon_864	76.3	76.5	80.4	70.9
p__Bacteroidetes	c__Flavobacteriia	g__Capnocytophaga	s__sp._oral_taxon_902	45.4	51.0	43.5	44.7
p__Bacteroidetes	c__Flavobacteriia	g__Capnocytophaga	s__sp._oral_taxon_903	37.1	26.6	37.4	42.5
p__Bacteroidetes	c__Flavobacteriia	g__Capnocytophaga	s__sputigena	416.4	448.8	405.0	413.0
p__Chloroflexi	c__Anaerolineae	g__Anaerolineae_[G-1]	s__sp._oral_taxon_439	59.1	30.1	54.2	81.8
p__Firmicutes	c__Bacilli	g__Abiotrophia	s__defectiva	104.2	135.7	109.9	79.0
p__Firmicutes	c__Bacilli	g__Gemella	s__haemolysans	338.8	412.1	338.9	297.4
p__Firmicutes	c__Bacilli	g__Gemella	s__morbillorum	603.8	670.7	622.2	542.2
p__Firmicutes	c__Bacilli	g__Gemella	s__sanguinis	44.3	33.8	36.2	60.7
p__Firmicutes	c__Bacilli	g__Granulicatella	s__adiacens	532.6	564.2	523.2	527.1
p__Firmicutes	c__Bacilli	g__Granulicatella	s__elegans	39.7	49.7	37.7	36.7
p__Firmicutes	c__Bacilli	g__Lactobacillus	s__gasseri	31.4	9.3	37.7	35.5
p__Firmicutes	c__Bacilli	g__Streptococcus	s__anginosus	479.8	409.3	468.1	534.6
p__Firmicutes	c__Bacilli	g__Streptococcus	s__australis	28.9	21.0	24.1	39.5
p__Firmicutes	c__Bacilli	g__Streptococcus	s__constellatus	283.7	208.0	279.1	332.2
p__Firmicutes	c__Bacilli	g__Streptococcus	s__cristatus	516.2	457.7	546.4	509.7
p__Firmicutes	c__Bacilli	g__Streptococcus	s__gordonii	998.1	853.8	991.3	1088
p__Firmicutes	c__Bacilli	g__Streptococcus	s__intermedius	897.1	1038	949.4	749.9
p__Firmicutes	c__Bacilli	g__Streptococcus	s__lactarius	56.8	97.9	40.2	55.3
p__Firmicutes	c__Bacilli	g__Streptococcus	s__mutans	530.8	392.2	500.4	648.0
p__Firmicutes	c__Bacilli	g__Streptococcus	s__oralis	6725	8031	6651	6089
p__Firmicutes	c__Bacilli	g__Streptococcus	s__parasanguinis_I	59.7	48.6	51.2	77.0
p__Firmicutes	c__Bacilli	g__Streptococcus	s__parasanguinis_II	141.3	124.2	126.4	170.2
p__Firmicutes	c__Bacilli	g__Streptococcus	s__salivarius	460.0	399.7	455.1	500.2
p__Firmicutes	c__Bacilli	g__Streptococcus	s__sanguinis	1128	1441	1133	945.5
p__Firmicutes	c__Bacilli	g__Streptococcus	s__sinensis	29.3	32.1	14.3	47.4
p__Firmicutes	c__Bacilli	g__Streptococcus	s__sobrinus	52.0	2.5	19.9	121.7
p__Firmicutes	c__Bacilli	g__Streptococcus	s__sp._oral_taxon_056	90.0	102.7	82.4	92.7
p__Firmicutes	c__Bacilli	g__Streptococcus	s__sp._oral_taxon_074	68.3	54.3	71.9	71.6
p__Firmicutes	c__Clostridia	g__Butyrivibrio	s__sp._oral_taxon_080	24.6	14.1	35.9	15.9
p__Firmicutes	c__Clostridia	g__Catonella	s__morbi	230.1	228.9	222.5	240.8
p__Firmicutes	c__Clostridia	g__Filifactor	s__alocis	368.2	274.3	418.9	355.0
p__Firmicutes	c__Clostridia	g__Johnsonella	s__ignava	114.9	104.7	111.8	124.5
p__Firmicutes	c__Clostridia	g__Johnsonella	s__sp._oral_taxon_166	32.7	17.7	43.8	26.7
p__Firmicutes	c__Clostridia	g__Lachnoanaerobaculum	s__orale	31.4	35.3	24.9	37.6
p__Firmicutes	c__Clostridia	g__Lachnoanaerobaculum	s__saburreum	152.3	124.4	153.0	167.0
p__Firmicutes	c__Clostridia	g__Lachnoanaerobaculum	s__umeaense	52.9	50.6	55.5	50.7
p__Firmicutes	c__Clostridia	g__Lachnospiraceae_[G-3]	s__sp._oral_taxon_100	132.2	118.4	143.0	125.9
p__Firmicutes	c__Clostridia	g__Lachnospiraceae_[G-8]	s__sp._oral_taxon_500	45.1	40.6	48.1	43.7
p__Firmicutes	c__Clostridia	g__Oribacterium	s__sp._oral_taxon_078	112.2	68.2	102.9	149.0
p__Firmicutes	c__Clostridia	g__Parvimonas	s__micra	848.4	791.4	915.0	793.8
p__Firmicutes	c__Clostridia	g__Parvimonas	s__sp._oral_taxon_393	265.1	361.3	250.6	230.0
p__Firmicutes	c__Clostridia	g__Peptostreptococcaceae_[XI][G-1]	s__[Eubacterium]_infirmum	38.9	32.2	32.2	51.2
p__Firmicutes	c__Clostridia	g__Peptostreptococcaceae_[XI][G-5]	s__[Eubacterium]_saphenum	87.3	55.6	107.1	79.2
p__Firmicutes	c__Clostridia	g__Peptostreptococcaceae_[XI][G-6]	s__[Eubacterium]_nodatum	64.3	60.2	55.8	77.7
p__Firmicutes	c__Clostridia	g__Peptostreptococcaceae_[XI][G-7]	s__[Eubacterium]_yurii_subsps._yur	146.2	167.1	151.3	127.9
p__Firmicutes	c__Clostridia	g__Peptostreptococcaceae_[XI][G-9]	s__[Eubacterium]_brachy	200.8	208.5	203.6	192.7
p__Firmicutes	c__Clostridia	g__Peptostreptococcus	s__stomatis	107.9	130.7	103.5	100.7
p__Firmicutes	c__Clostridia	g__Pseudoramibacter	s__alactolyticus	70.8	73.3	52.7	93.0
p__Firmicutes	c__Clostridia	g__Ruminococcaceae_[G-1]	s__sp._oral_taxon_075	83.8	109.4	79.6	74.9
p__Firmicutes	c__Clostridia	g__Shuttleworthia	s__satelles	35.3	21.3	40.5	36.3
p__Firmicutes	c__Clostridia	g__Stomatobaculum	s__longum	55.2	47.4	52.5	63.0
p__Firmicutes	c__Erysipelotrichia	g__Solobacterium	s__moorei	42.1	40.4	41.0	44.6
p__Firmicutes	c__Mollicutes	g__Mycoplasma	s__salivarium	33.0	24.9	30.9	40.4
p__Firmicutes	c__Negativicutes	g__Anaeroglobus	s__geminatus	767.6	402.1	582.4	1214
p__Firmicutes	c__Negativicutes	g__Centipeda	s__periodontii	93.0	45.3	106.0	102.9
p__Firmicutes	c__Negativicutes	g__Dialister	s__invisus	612.2	466.3	625.5	676.8
p__Firmicutes	c__Negativicutes	g__Dialister	s__pneumosintes	225.5	179.8	224.8	252.1
p__Firmicutes	c__Negativicutes	g__Megasphaera	s__micronuciformis	208.0	136.7	212.5	242.2
p__Firmicutes	c__Negativicutes	g__Megasphaera	s__sp._oral_taxon_123	158.7	166.4	138.4	180.8
p__Firmicutes	c__Negativicutes	g__Mitsuokella	s__sp._oral_taxon_131	117.5	40.4	143.3	127.2
p__Firmicutes	c__Negativicutes	g__Mitsuokella	s__sp._oral_taxon_521	31.2	11.1	13.3	65.7
p__Firmicutes	c__Negativicutes	g__Selenomonas	s__artemidis	738.5	720.4	712.4	782.7
p__Firmicutes	c__Negativicutes	g__Selenomonas	s__dianae	47.4	36.6	36.3	67.9
p__Firmicutes	c__Negativicutes	g__Selenomonas	s__flueggei	166.3	137.2	160.7	190.0
p__Firmicutes	c__Negativicutes	g__Selenomonas	s__infelix	272.6	194.0	268.0	322.7
p__Firmicutes	c__Negativicutes	g__Selenomonas	s__noxia	1502	1474	1514	1500
p__Firmicutes	c__Negativicutes	g__Selenomonas	s__sp._oral_taxon_126	102.4	92.1	105.4	104.2
p__Firmicutes	c__Negativicutes	g__Selenomonas	s__sp._oral_taxon_133	44.7	44.7	43.4	46.3
p__Firmicutes	c__Negativicutes	g__Selenomonas	s__sp._oral_taxon_134	357.6	200.4	361.3	440.9
p__Firmicutes	c__Negativicutes	g__Selenomonas	s__sp._oral_taxon_136	318.2	182.9	317.6	394.8
p__Firmicutes	c__Negativicutes	g__Selenomonas	s__sp._oral_taxon_137	494.2	423.6	520.8	499.2
p__Firmicutes	c__Negativicutes	g__Selenomonas	s__sp._oral_taxon_146	119.4	98.3	117.8	133.2
p__Firmicutes	c__Negativicutes	g__Selenomonas	s__sp._oral_taxon_149	19.7	9.7	25.0	18.4
p__Firmicutes	c__Negativicutes	g__Selenomonas	s__sp._oral_taxon_442	27.3	14.0	37.6	21.3
p__Firmicutes	c__Negativicutes	g__Selenomonas	s__sp._oral_taxon_478	15.5	12.0	12.6	21.2
p__Firmicutes	c__Negativicutes	g__Selenomonas	s__sp._oral_taxon_892	223.3	222.2	211.7	239.0
p__Firmicutes	c__Negativicutes	g__Selenomonas	s__sp._oral_taxon_919	187.4	170.1	178.5	208.7
p__Firmicutes	c__Negativicutes	g__Selenomonas	s__sp._oral_taxon_936	83.7	62.0	73.5	109.1
p__Firmicutes	c__Negativicutes	g__Selenomonas	s__sp._oral_taxon_937	22.1	14.6	22.5	25.7
p__Firmicutes	c__Negativicutes	g__Selenomonas	s__sputigena	3283	2195	2957	4319
p__Firmicutes	c__Negativicutes	g__Veillonella	s__atypica	586.0	380.8	569.5	722.5
p__Firmicutes	c__Negativicutes	g__Veillonella	s__denticariosi	201.2	265.6	159.9	218.6
p__Firmicutes	c__Negativicutes	g__Veillonella	s__dispar	8720	7556	8534	9615
p__Firmicutes	c__Negativicutes	g__Veillonella	s__parvula	6529	5964	6121	7376
p__Firmicutes	c__Negativicutes	g__Veillonella	s__rogosae	202.3	138.4	208.0	230.9
p__Firmicutes	c__Negativicutes	g__Veillonella	s__sp._oral_taxon_780	109.3	147.1	100.8	99.2
p__Firmicutes	c__Negativicutes	g__Veillonellaceae_[G-1]	s__sp._oral_taxon_129	54.4	27.0	45.9	81.0
p__Firmicutes	c__Negativicutes	g__Veillonellaceae_[G-1]	s__sp._oral_taxon_145	59.1	39.5	58.4	70.9
p__Firmicutes	c__Negativicutes	g__Veillonellaceae_[G-1]	s__sp._oral_taxon_150	295.0	204.8	251.6	402.1
p__Firmicutes	c__Negativicutes	g__Veillonellaceae_[G-1]	s__sp._oral_taxon_155	265.6	194.6	212.4	374.6
p__Fusobacteria	c__Fusobacteriia	g__Fusobacterium	s__naviforme	826.5	725.4	813.9	899.6
p__Fusobacteria	c__Fusobacteriia	g__Fusobacterium	s__nucleatum_subsp._animalis	1650	1387	1575	1896
p__Fusobacteria	c__Fusobacteriia	g__Fusobacterium	s__nucleatum_subsp._nucleatum	223.2	165.8	149.4	351.4
p__Fusobacteria	c__Fusobacteriia	g__Fusobacterium	s__nucleatum_subsp._polymorphum	1439	1467	1499	1345
p__Fusobacteria	c__Fusobacteriia	g__Fusobacterium	s__nucleatum_subsp._vincentii	3930	3647	3798	4262
p__Fusobacteria	c__Fusobacteriia	g__Fusobacterium	s__periodonticum	87.3	57.2	88.7	102.2
p__Fusobacteria	c__Fusobacteriia	g__Fusobacterium	s__sp._oral_taxon_203	1968	1576	2223	1857
p__Fusobacteria	c__Fusobacteriia	g__Fusobacterium	s__sp._oral_taxon_370	34.1	36.1	37.0	29.2
p__Fusobacteria	c__Fusobacteriia	g__Leptotrichia	s__buccalis	399.0	365.5	436.8	368.7
p__Fusobacteria	c__Fusobacteriia	g__Leptotrichia	s__goodfellowii	22.9	26.0	24.7	18.7
p__Fusobacteria	c__Fusobacteriia	g__Leptotrichia	s__hofstadii	346.7	418.6	364.3	283.4
p__Fusobacteria	c__Fusobacteriia	g__Leptotrichia	s__hongkongensis	388.3	297.8	360.4	475.3
p__Fusobacteria	c__Fusobacteriia	g__Leptotrichia	s__shahii	387.0	312.2	250.1	607.1
p__Fusobacteria	c__Fusobacteriia	g__Leptotrichia	s__sp._oral_taxon_212	253.4	237.4	264.5	248.1
p__Fusobacteria	c__Fusobacteriia	g__Leptotrichia	s__sp._oral_taxon_215	107.2	117.0	107.5	101.2
p__Fusobacteria	c__Fusobacteriia	g__Leptotrichia	s__sp._oral_taxon_219	36.9	37.3	35.4	38.6
p__Fusobacteria	c__Fusobacteriia	g__Leptotrichia	s__sp._oral_taxon_223	78.4	39.0	82.6	95.1
p__Fusobacteria	c__Fusobacteriia	g__Leptotrichia	s__sp._oral_taxon_225	244.7	299.5	328.2	105.5
p__Fusobacteria	c__Fusobacteriia	g__Leptotrichia	s__sp._oral_taxon_392	195.9	187.9	212.8	178.3
p__Fusobacteria	c__Fusobacteriia	g__Leptotrichia	s__sp._oral_taxon_417	363.2	241.0	408.8	372.5
p__Fusobacteria	c__Fusobacteriia	g__Leptotrichia	s__sp._oral_taxon_498	210.4	135.1	176.6	296.4
p__Fusobacteria	c__Fusobacteriia	g__Leptotrichia	s__sp._oral_taxon_879	52.3	57.8	31.4	76.5
p__Fusobacteria	c__Fusobacteriia	g__Leptotrichia	s__wadei	767.1	783.8	675.4	877.0
p__Gracilibacteria_(GN02)	c__GN02_[C-2]	g__GN02_[G-2]	s__sp._oral_taxon_873	24.8	31.7	18.7	29.0
p__Proteobacteria	c__Alphaproteobacteria	g__Bradyrhizobium	s__elkanii	31.9	36.6	31.5	29.8
p__Proteobacteria	c__Alphaproteobacteria	g__Brevundimonas	s__diminuta	0.6	0.9	0.5	0.6
p__Proteobacteria	c__Alphaproteobacteria	g__Porphyrobacter	s__tepidarius	0.2	0.1	0.1	0.3
p__Proteobacteria	c__Alphaproteobacteria	g__Sphingomonas	s__echinoides	9.2	8.0	9.3	9.7
p__Proteobacteria	c__Alphaproteobacteria	g__Sphingomonas	s__sp._oral_taxon_006	0.5	0.3	0.2	1.1
p__Proteobacteria	c__Betaproteobacteria	g__Eikenella	s__corrodens	264.4	284.9	279.0	233.9
p__Proteobacteria	c__Betaproteobacteria	g__Kingella	s__denitrificans	159.5	149.8	146.3	182.3
p__Proteobacteria	c__Betaproteobacteria	g__Kingella	s__oralis	311.9	324.2	285.2	339.8
p__Proteobacteria	c__Betaproteobacteria	g__Lautropia	s__mirabilis	145.1	173.9	144.3	129.9
p__Proteobacteria	c__Betaproteobacteria	g__Leptothrix	s__sp._oral_taxon_025	0.3	0.6	0.4	0.2
p__Proteobacteria	c__Betaproteobacteria	g__Neisseria	s__bacilliformis	84.2	70.1	80.2	97.4
p__Proteobacteria	c__Betaproteobacteria	g__Neisseria	s__elongata	499.3	682.7	506.5	387.0
p__Proteobacteria	c__Betaproteobacteria	g__Neisseria	s__flavescens	440.2	302.3	352.9	631.0
p__Proteobacteria	c__Betaproteobacteria	g__Neisseria	s__oralis	376.2	558.0	272.4	409.2
p__Proteobacteria	c__Betaproteobacteria	g__Neisseria	s__pharyngis	70.1	86.1	41.5	98.3
p__Proteobacteria	c__Betaproteobacteria	g__Neisseria	s__sicca	730.6	656.1	784.9	701.7
p__Proteobacteria	c__Betaproteobacteria	g__Neisseria	s__subflava	174.1	125.9	194.7	174.4
p__Proteobacteria	c__Betaproteobacteria	g__Ottowia	s__sp._oral_taxon_894	70.0	88.8	70.2	59.3
p__Proteobacteria	c__Deltaproteobacteria	g__Desulfobulbus	s__sp._oral_taxon_041	139.1	91.1	132.8	174.4
p__Proteobacteria	c__Epsilonproteobacteria	g__Campylobacter	s__concisus	242.1	186.5	241.4	274.2
p__Proteobacteria	c__Epsilonproteobacteria	g__Campylobacter	s__curvus	39.6	46.6	31.6	46.1
p__Proteobacteria	c__Epsilonproteobacteria	g__Campylobacter	s__gracilis	858.3	734.1	849.7	939.1
p__Proteobacteria	c__Epsilonproteobacteria	g__Campylobacter	s__showae	496.9	422.2	521.4	507.0
p__Proteobacteria	c__Gammaproteobacteria	g__Aggregatibacter	s__actinomycetemcomitans	49.2	68.1	46.4	42.2
p__Proteobacteria	c__Gammaproteobacteria	g__Aggregatibacter	s__aphrophilus	312.9	232.7	392.3	254.5
p__Proteobacteria	c__Gammaproteobacteria	g__Aggregatibacter	s__paraphrophilus	111.5	126.7	141.8	63.6
p__Proteobacteria	c__Gammaproteobacteria	g__Aggregatibacter	s__segnis	233.8	295.3	259.7	165.6
p__Proteobacteria	c__Gammaproteobacteria	g__Aggregatibacter	s__sp._oral_taxon_458	158.1	130.8	151.6	181.8
p__Proteobacteria	c__Gammaproteobacteria	g__Aggregatibacter	s__sp._oral_taxon_513	61.5	35.1	85.1	45.7
p__Proteobacteria	c__Gammaproteobacteria	g__Cardiobacterium	s__hominis	277.9	342.4	295.3	219.1
p__Proteobacteria	c__Gammaproteobacteria	g__Cardiobacterium	s__valvarum	234.5	257.9	245.6	207.0
p__Proteobacteria	c__Gammaproteobacteria	g__Haemophilus	s__haemolyticus	50.2	95.7	34.4	45.4
p__Proteobacteria	c__Gammaproteobacteria	g__Haemophilus	s__parahaemolyticus	78.5	71.7	121.9	26.0
p__Proteobacteria	c__Gammaproteobacteria	g__Haemophilus	s__parainfluenzae	1042	1480	940.9	927.4
p__Proteobacteria	c__Gammaproteobacteria	g__Haemophilus	s__sp._oral_taxon_036	75.0	82.2	71.0	76.2
p__Proteobacteria	c__Gammaproteobacteria	g__Pseudomonas	s__fluorescens	52.5	52.2	53.4	51.6
p__SR1	c__SR1_[C-1]	g__SR1_[G-1]	s__sp._oral_taxon_874	26.9	26.4	29.2	24.3
p__Saccharibacteria_(TM7)	c__TM7_[C-1]	g__TM7_[G-1]	s__sp._oral_taxon_346	1103	901.9	1098	1223
p__Saccharibacteria_(TM7)	c__TM7_[C-1]	g__TM7_[G-1]	s__sp._oral_taxon_347	100.0	143.7	105.6	68.1
p__Saccharibacteria_(TM7)	c__TM7_[C-1]	g__TM7_[G-1]	s__sp._oral_taxon_348	106.9	75.8	123.5	102.9
p__Saccharibacteria_(TM7)	c__TM7_[C-1]	g__TM7_[G-1]	s__sp._oral_taxon_349	1107	900.9	1062	1279
p__Saccharibacteria_(TM7)	c__TM7_[C-1]	g__TM7_[G-1]	s__sp._oral_taxon_352	49.4	42.8	43.6	60.5
p__Saccharibacteria_(TM7)	c__TM7_[C-1]	g__TM7_[G-1]	s__sp._oral_taxon_488	136.2	146.7	156.0	104.4
p__Saccharibacteria_(TM7)	c__TM7_[C-1]	g__TM7_[G-1]	s__sp._oral_taxon_869	125.1	120.6	128.5	123.1
p__Saccharibacteria_(TM7)	c__TM7_[C-1]	g__TM7_[G-1]	s__sp._oral_taxon_952	665.5	706.5	669.6	637.1
p__Saccharibacteria_(TM7)	c__TM7_[C-1]	g__TM7_[G-2]	s__sp._oral_taxon_350	156.1	137.7	139.2	188.3
p__Saccharibacteria_(TM7)	c__TM7_[C-1]	g__TM7_[G-3]	s__sp._oral_taxon_351	33.2	21.1	35.6	36.9
p__Saccharibacteria_(TM7)	c__TM7_[C-1]	g__TM7_[G-5]	s__sp._oral_taxon_356	616.8	371.0	677.8	675.5
p__Saccharibacteria_(TM7)	c__TM7_[C-1]	g__TM7_[G-6]	s__sp._oral_taxon_870	82.6	93.6	83.9	74.6
p__Spirochaetes	c__Spirochaetia	g__Treponema	s__denticola	372.7	246.3	374.0	442.1
p__Spirochaetes	c__Spirochaetia	g__Treponema	s__lecithinolyticum	65.5	62.1	65.8	67.0
p__Spirochaetes	c__Spirochaetia	g__Treponema	s__maltophilum	83.6	56.0	73.1	112.7
p__Spirochaetes	c__Spirochaetia	g__Treponema	s__medium	45.7	27.4	60.7	36.3
p__Spirochaetes	c__Spirochaetia	g__Treponema	s__socranskii	297.0	211.8	294.6	348.0
p__Spirochaetes	c__Spirochaetia	g__Treponema	s__sp._oral_taxon_231	102.7	75.9	117.3	98.6
p__Spirochaetes	c__Spirochaetia	g__Treponema	s__sp._oral_taxon_237	160.6	104.6	160.5	192.0
p__Spirochaetes	c__Spirochaetia	g__Treponema	s__sp._oral_taxon_247	44.0	8.2	68.9	31.7
p__Spirochaetes	c__Spirochaetia	g__Treponema	s__vincentii	27.6	36.1	33.3	15.4
p__Synergistetes	c__Synergistia	g__Fretibacterium	s__fastidiosum	551.9	462.9	595.3	545.5
p__Synergistetes	c__Synergistia	g__Fretibacterium	s__sp._oral_taxon_358	81.3	46.0	93.4	85.3
p__Synergistetes	c__Synergistia	g__Fretibacterium	s__sp._oral_taxon_359	722.3	648.0	814.1	644.6
p__Synergistetes	c__Synergistia	g__Fretibacterium	s__sp._oral_taxon_360	1247	818.0	1325	1385
p__Synergistetes	c__Synergistia	g__Fretibacterium	s__sp._oral_taxon_361	54.1	4.5	61.8	71.9
p__Synergistetes	c__Synergistia	g__Fretibacterium	s__sp._oral_taxon_362	212.8	161.8	233.9	213.9
p__Synergistetes	c__Synergistia	g__Pyramidobacter	s__piscolens	5.7	7.9	4.6	5.9

For three known highly virulent periodontal pathogens, *Porphyromonas ginigivalis* (*Bacteroidetes phylum*), *Tannerella forsythia* (*Bacteroidetes phylum*), and *Treponema denticola* (*Spirochaetes phylum*), overall mean reads were 1055, 577.6, and 372.7, respectively; mean reads for each increased with age. Mean reads for bacteria typically associated with periodontal health (*Streptococcus oralis, sanguinis and intermedius*; *Firmicutes phylum*) were 6725, 1128, and 897; each decreasing across incremental age groups. To further evaluate the distribution of the two predominant phyla, we computed the *Firmicutes-to-Bacteroidetes* ratio by summing the mean reads separately within each of these phyla (Table 2) and then creating a ratio of these sums. The ratio was 1.56 among all women, and increased with age: 1.45 (50–69 years); 1.55 (60–69 years); and 1.61 (≥70 years).

**Fig. 3:**
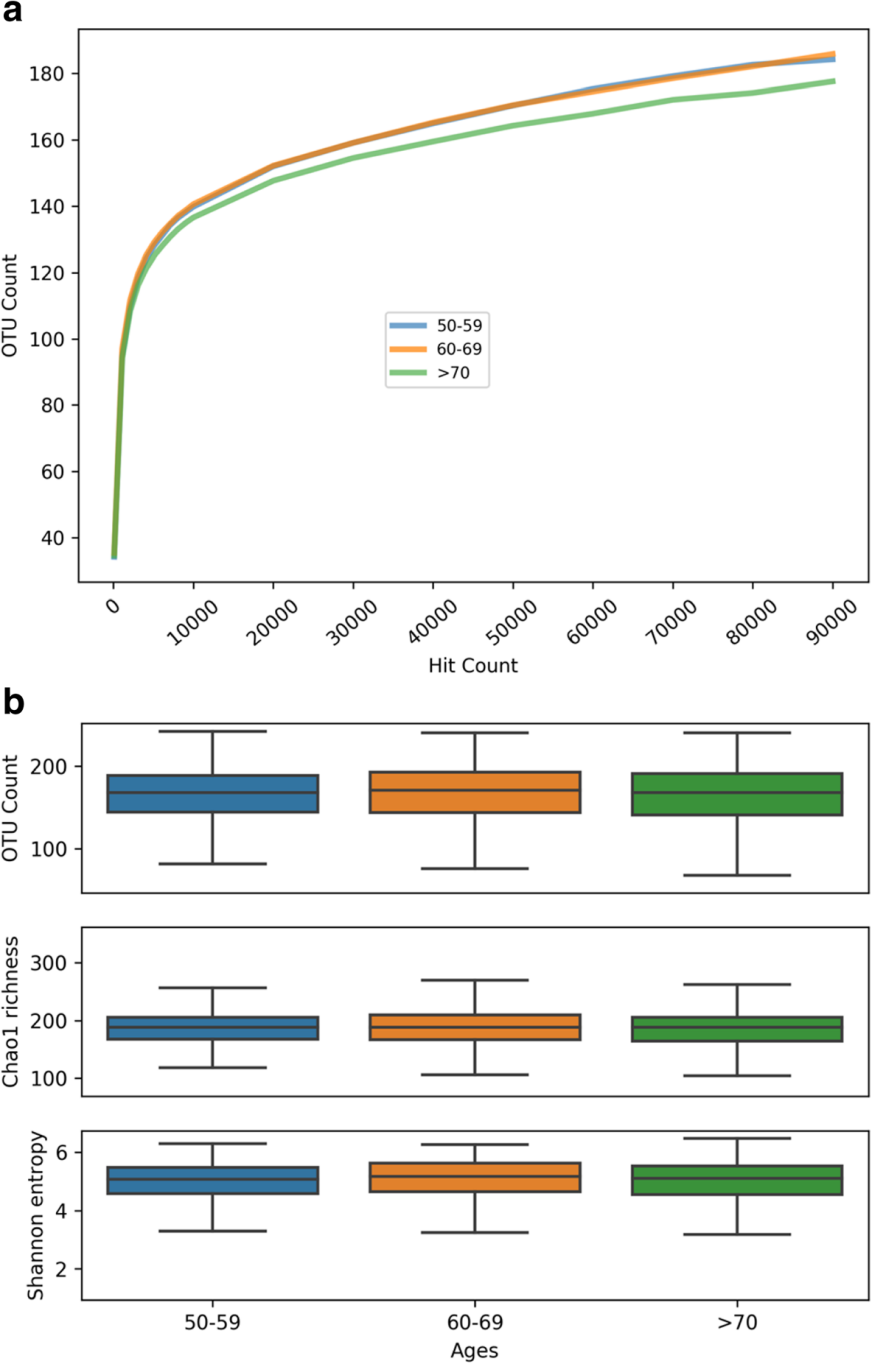
Alpha (within-group) diversity of identified taxa according to age groups. Panel **a** gives the rarefaction curve and Panel **b** gives measures of richness (Chao-1, *P* = 0.55; OTU counts, *P* = 0.35) and evenness (Shannon entropy, *P* = 0.42)

**Fig. 4:**
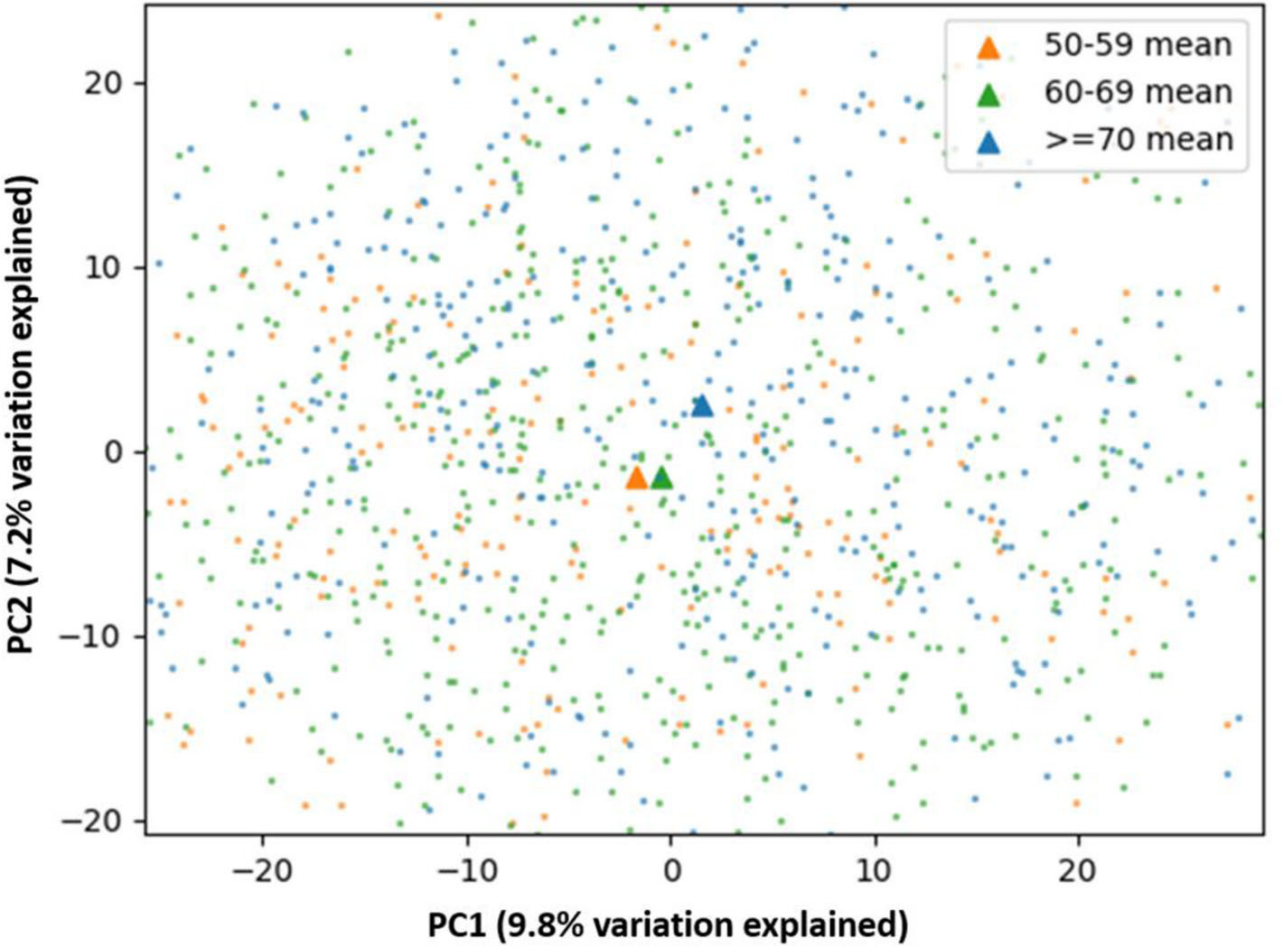
Beta (between-group) diversity of identified taxa according to age groups. Permutation MANOVA test yielded *P* = 0.001, suggesting differences are present in mean vectors (triangles) across age categories, despite unclear clustering in the PCA plot

We next evaluated alpha (within-group) and beta (between-group) diversity of the bacterial species in the overall cohort and according to age categories. For alpha (within-group) diversity, mean (SD), OTU count richness, Chao1 richness, and Shannon entropy evenness were 165 (45.1), 185.0 (31.2), and 5.0 (0.7), respectively, among all women, and remained consistent across age categories (Fig. 3). Beta (between-group) diversity is shown in the PCA plot in Fig. 4. A Permutation MANOVA test yielded *P* = 0.001, suggesting that differences were present in mean vectors across age categories, despite unclear clustering in the PCA plot itself.

Table 3 presents the top 20 highest and top 20 lowest OTUs based on their CLR mean for the overall cohort and according to age categories. Also shown in Table 3 are linear correlations between these OTUs and age, as well as an indication of whether or not the OTU has previously been cultured and named in the HOMD, and a notation of membership within the Socransky color complex of bacterial species previously identified using targeted methods [34]. *Veillonella dispar* (CLR mean, 8.3)*, S. Oralis* (CLR mean 8.1), and *Veillonella parvula* (CLR mean, 7.6) had the greatest abundance, about a 256-fold (2^8^) higher than the overall composition mean. There were 18 (90%) taxa with a 16-fold or greater (CLR mean ≥ 4) elevation in abundance based on CLR mean OTUs. Among the top 20 most abundant bacteria, 19 (95%) were previously named, whereas one (5%) was previously unnamed in HOMD. Among the top 20 taxa were bacteria previously associated with both periodontal health (*S. oralis, sanguinis, gordonii, and intermedius*) and periodontal disease (*V. parvula; Fusobacterium nucleatum; Parvomonas micra; Prevotella nigrescens; Rothia dentocariosa; Actinomyces naeslundii*). Ten of the top 20 bacteria were included in Socransky’s complex organization, with four (20%) from the *yellow* complex typically associated with healthy periodontium and four (20%) from the *orange* complex which is associated with periodontitis. Among taxa with reduced abundance, *Porphyrobacter tepidarius* (CLR mean, − 3.6), *Sphingomonas sp._oral_taxon 006* (CLR mean, − 3.6), *Pyramidobacter piscolens* (CLR mean, − 3.5), *Leptothirix sp._oral_taxon 025* (CLR mean, − 3.5), and *Treponema sp._oral_taxon 247* (CLR mean, − 3.5) each had a 11-fold or lower abundance relative to the overall composition mean. Seven (35%) of the 20 least abundant bacteria have been previously named in HOMD; two (10%) are unnamed; and, 11 (55%) have been phylotyped, but as yet not named.

**Table 3:** Top 20 highest* and lowest* mean CLR OTU for the overall cohort and by age categories, and their linear correlation with age

Rank Order*	OTU Label	Culture Status	Socransky Complex	Overall Cohort (*N* = 1219)	Age Categories (years)				Linear Correlation	
					50–59 (*N* = 239)	60–69 (*N* = 554)	≥70 (*N* = 426)	*p*-value	Pearson r	*p*-value
				CLR OTU Mean (SE)	CLR OTU Mean (SE)	CLR OTU Mean (SE)	CLR OTU Mean (SE)			
20 Most Abundant Species										
1	Veillonella dispar	N	–	8.25 (0.06)	8.23 (0.13)	8.11 (0.09)	8.45 (0.10)	**0.045**	0.08	**0.008**
2	Streptococcus oralis	N	Y	8.06 (0.05)	8.40 (0.11)	8.06 (0.08)	7.87 (0.09)	**0.002**	−0.10	**<.001**
3	Veillonella parvula	N	P	7.60 (0.07)	7.48 (0.15)	7.45 (0.09)	7.86 (0.11)	**0.014**	0.10	**0.001**
4	Fusobacterium nucleatum_subsp._vincentii	N	O	6.43 (0.08)	6.21 (0.17)	6.48 (0.11)	6.50 (0.13)	0.356	0.02	0.587
5	Selenomonas sputigena	N	–	5.63 (0.08)	5.34 (0.17)	5.44 (0.12)	6.03 (0.14)	**0.001**	0.10	**<.001**
6	Fusobacterium nucleatum_subsp._animalis	N	–	5.39 (0.07)	5.29 (0.14)	5.29 (0.10)	5.57 (0.12)	0.136	0.10	0.106
7	Campylobacter gracilis	N	O	5.19 (0.05)	5.02 (0.11)	5.15 (0.07)	5.33 (0.09)	0.052	0.10	**0.016**
8	Fusobacterium nucleatum_subsp._polymorphum	N	O	5.19 (0.07)	5.36 (0.15)	5.28 (0.09)	4.97 (0.12)	0.052	−0.10	**0.019**
9	Prevotella oris	N	–	5.08 (0.09)	5.25 (0.18)	5.05 (0.13)	5.03 (0.16)	0.646	−0.10	0.118
10	Streptococcus sanguinis	N	Y	4.91 (0.07)	5.57 (0.15)	5.04 (0.10)	4.38 (0.13)	<.001*	−0.18	<.001*
11	Corynebacterium matruchotii	N	–	4.81 (0.07)	4.87 (0.17)	4.86 (0.10)	4.70 (0.12)	0.550	−0.04	0.171
12	Selenomonas noxia	N	–	4.81 (0.08)	4.63 (0.18)	4.69 (0.11)	5.06 (0.13)	**0.049**	0.10	**<.001**
13	Prevotella nigrescens	N	O	4.31 (0.10)	4.48 (0.21)	4.28 (0.15)	4.26 (0.18)	0.714	− 0.03	0.370
14	Parvimonas micra	N	–	4.29 (0.08)	4.08 (0.17)	4.35 (0.11)	4.33 (0.12)	0.401	0.00	0.967
15	Rothia dentocariosa	U	–	4.28 (0.09)	4.46 (0.19)	4.38 (0.13)	4.04 (0.15)	0.113	−0.10	**0.008**
16	Fusobacterium sp._oral_taxon_203	N	–	4.23 (0.10)	4.08 (0.22)	4.36 (0.15)	4.16 (0.17)	0.497	−0.01	0.713
17	Streptococcus gordonii	N	Y	4.19 (0.08)	3.93 (0.18)	4.15 (0.12)	4.40 (0.13)	0.090	0.04	**0.039**
18	Granulicatella adiacens	N	–	4.15 (0.06)	4.23 (0.14)	4.11 (0.09)	4.15 (0.10)	0.749	0.00	0.991
19	Streptococcus intermedius	N	Y	3.94 (0.10)	4.33 (0.21)	3.86 (0.15)	3.81 (0.16)	0.119	−0.10	**0.016**
20	Actinomyces naeslundii	N	B	3.85 (0.06)	4.00 (0.14)	3.88 (0.09)	3.72 (0.11)	0.230	−0.10	0.008
20 Least Abundant Species										
1	Porphyrobacter tepidarius	N	–	− 3.58 (0.03)	−3.51 (0.08)	− 3.65 (0.05)	− 3.54 (0.06)	0.216	0.01	0.869
2	Sphingomonas sp._oral_taxon_006	P	–	− 3.55 (0.04)	− 3.49 (0.08)	− 3.60 (0.05)	− 3.52 (0.06)	0.407	−0.00	0.989
3	Pyramidobacter piscolens	P	–	− 3.53 (0.04)	− 3.44 (0.10)	− 3.60 (0.06)	− 3.47 (0.07)	0.251	− 0.01	0.919
4	Leptothrix sp._oral_taxon_025	P	–	−3.50 (0.04)	− 3.37 (0.08)	− 3.53 (0.06)	−3.53 (0.06)	0.216	−0.04	0.221
5	Treponema sp._oral_taxon_247	P	–	−3.45 (0.05)	−3.35 (0.09)	− 3.50 (0.07)	−3.44 (0.08)	0.436	− 0.02	0.566
6	Atopobium sp._oral_taxon_416	P	–	−3.38 (0.05)	−3.45 (0.09)	− 3.44 (0.07)	−3.27 (0.09)	0.202	0.10	**0.042**
7	Brevundimonas diminuta	N	–	−3.30 (0.04)	−3.17 (0.09)	− 3.35 (0.06)	−3.30 (0.07)	0.232	−0.03	0.306
8	Prevotella multiformis	N	–	−3.08 (0.06)	−3.12 (0.11)	− 3.10 (0.09)	−3.05 (0.10)	0.901	0.03	0.359
9	GN02_[G-2] sp._oral_taxon_873	P	–	−3.06 (0.05)	−3.08 (0.12)	− 3.03 (0.08)	−3.10 (0.09)	0.851	−0.00	0.927
10	Streptococcus sobrinus	U	–	−3.04 (0.06)	−3.21 (0.10)	− 3.19 (0.09)	−2.75 (0.14)	**0.005**	0.11	<.001*
11	Aggregatibacter actinomycetemcomitans	P	–	− 3.01 (0.07)	− 2.97 (0.15)	− 3.09 (0.10)	− 2.94 (0.11)	0.575	0.03	0.349
12	Fretibacterium sp._oral_taxon_361	P	–	−2.98 (0.06)	−3.18 (0.10)	−2.97 (0.09)	− 2.88 (0.11)	0.207	0.02	0.469
13	Butyrivibrio sp._oral_taxon_080	P	–	−2.96 (0.06)	−2.85 (0.13)	− 2.90 (0.09)	−3.10 (0.09)	0.193	−0.10	**0.038**
14	Lactobacillus gasseri	N	–	−2.95 (0.06)	−3.08 (0.12)	−3.09 (0.09)	−2.70 (0.12)	**0.012**	0.10	**0.002**
15	Mitsuokella sp._oral_taxon_521	N	–	−2.90 (0.06)	−2.91 (0.12)	− 2.92 (0.08)	−2.87 (0.11)	0.929	0.02	0.603
16	Microbacterium flavescens	P	–	−2.89 (0.04)	−2.73 (0.09)	− 2.89 (0.06)	−2.97 (0.07)	0.151	−0.10	0.051
17	Prevotella sp._oral_taxon_475	N	–	−2.87 (0.06)	−2.81 (0.12)	− 2.84 (0.09)	−2.95 (0.09)	0.575	−0.04	0.157
18	Neisseria pharyngis	N	–	−2.87 (0.06)	−2.81 (0.13)	− 2.95 (0.09)	−2.80 (0.11)	0.479	0.01	0.760
19	Fretibacterium sp._oral_taxon_358	U	–	−2.84 (0.07)	−2.87 (0.14)	− 2.88 (0.10)	−2.77 (0.12)	0.708	0.02	0.494
20	Treponema medium	P	–	−2.79 (0.06)	−2.85 (0.14)	− 2.74 (0.10)	−2.81 (0.10)	0.777	0.02	0.579

*OTUs are ranked according to mean CLR OTU. The 20 most abundant OTUs have positive mean CRL; the 20 least abundant OTUs have negative mean CLR

The CLR OTU can be interpreted as a log [2] fold-difference for the given species relative to the overall compositional geometric mean. A mean CLR of 3 indicates a 8-fold [23] higher abundance, and a mean CLR of −3 indicates a 8-fold lower abundance, relative to the overall compositional geometric mean

*SE* standard error, *Pearson r* the Pearson product-moment correlation coefficient

*p*-values: **bolded** are significant at alpha .05; asterisk are significant at alpha 0.05 after Bonferroni correction

Culture status annotation in HOMD: N = named; U = unnamed; P *=* phylotyped

Socransky complex^32^: R = red; O = orange; P = purple; G = green; Y = yellow; B = blue; −-- = not part of the Socransky classification

Linear correlations (Table 3) among the 20 most abundant bacteria ranged from *r* = − 0.18 to *r* = 0.10, with 11 (55%) of the correlations achieving statistical significance (uncorrected *P* < 0.05; bolded). After Bonferroni correction, only 1 (9%) of these remained statistically significant (*S. sanguinis*, *r* = − 0.18; corrected *P* < 0.001). Among the 20 least abundant bacteria, linear correlations ranged from − 0.10 to 0.11. Four (20%) correlations achieved statistical significance (uncorrected *P* < 0.05; bolded), of which 1 (25%) remained significant after Bonferonni correction (*Streptococcus sobrinus*, *r* = 0.11; corrected *P* < 0.001).

Differences in mean CLR across age categories achieved statistical significance (*P* < 0.05) for 8 (40%) of the 20 most abundant bacteria, of which only 1 (12.5%) remained significant following Bonferroni correction (*S. sanguinis*, corrected *P* < 0.001). Mean CLR differences across age categories among the least abundant bacteria were significant (*P* < 0.05) for two bacteria, neither of which remained significant after Bonferroni correction.

Table 4 presents the rank ordered mean CLR OTUs for all 267 taxa identified, as well as their linear correlations with age, culture status and Socransky classification. A total of 148 (55.4%) taxa had names previously annotated in the HOMD database, whereas 60 (22.5%) were unnamed and are OTUs potentially identifying new bacteria. In the overall cohort, 117 (43.8%) taxa demonstrated elevated abundance (CLR > 0), the remaining 150 (57.3%) demonstrating reduced abundance (CLR < 0), relative to the overall composition mean. Twenty eight (10.5%) taxa that demonstrated a 8-fold (i.e., 2^3^) or greater elevation in abundance based on mean CLR OTUs. There were 15 (5.6%) taxa with a 8-fold lower abundance relative to the overall composition mean. Of the virulent periodontal pathogens included in Socransky’s classification, [34] only *T. forsythia* (mean CLR, 1.87) and *F. nucleatum* (mean CLR, 6.4) had an elevated abundance, whereas *T. denticola* (mean CLR, − 0.28), *P. gingivalis* (mean CLR, − 0.56), *P. intermedia* (mean CLR, − 1.36) were, on average, in lower abundance. Several bacteria associated with healthy periodontium were in higher abundance: *S. oralis* (mean CLR, 5.5)*, sanguinis* (mean CLR, 3.4)*, gordonii* (mean CLR, 2.8)*, and intermedius* (mean CLR, 2.6); *P. micra* (mean CLR, 3.0).

**Table 4:** Mean CLR OTUs for each of 267 bacterial species in the overall cohort and by age categories, and its linear correlation with age

Rank Order*	OTU Label	Culture Status	Socransky Complex	Overall Cohort (*N* = 1219)	Age Categories (years)				Linear Correlation	
				CLR OTU Mean (SE)	50–59 (*N* = 239) CLR OTU Mean (SE)	60–69 (*N* = 554) CLR OTU Mean (SE)	≥70 (*N* = 426) CLR OTU Mean (SE)	*p*-value	Pearson r	*p*-value
1	Veillonella dispar	N	–	8.25 (0.06)	8.23 (0.13)	8.11 (0.09)	8.45 (0.10)	**0.045**	0.08	**0.008**
2	Streptococcus oralis	N	Y	8.06 (0.05)	8.40 (0.11)	8.06 (0.08)	7.87 (0.09)	**0.002**	−0.10	**<.001**
3	Veillonella parvula	N	P	7.60 (0.07)	7.48 (0.15)	7.45 (0.09)	7.86 (0.11)	**0.014**	0.10	**0.001**
4	Fusobacterium nucleatum_subsp._vincentii	N	O	6.43 (0.08)	6.21 (0.17)	6.48 (0.11)	6.50 (0.13)	0.356	0.02	0.587
5	Selenomonas sputigena	N	–	5.63 (0.08)	5.34 (0.17)	5.44 (0.12)	6.03 (0.14)	**0.001**	0.10	**<.001**
6	Fusobacterium nucleatum_subsp._animalis	N	–	5.39 (0.07)	5.29 (0.14)	5.29 (0.10)	5.57 (0.12)	0.136	0.10	0.106
7	Campylobacter gracilis	N	O	5.19 (0.05)	5.02 (0.11)	5.15 (0.07)	5.33 (0.09)	0.052	0.10	**0.016**
8	Fusobacterium nucleatum_subsp._polymorphum	N	O	5.19 (0.07)	5.36 (0.15)	5.28 (0.09)	4.97 (0.12)	0.052	−0.10	**0.019**
9	Prevotella oris	N	–	5.08 (0.09)	5.25 (0.18)	5.05 (0.13)	5.03 (0.16)	0.646	−0.10	0.118
10	Streptococcus sanguinis	N	Y	4.91 (0.07)	5.57 (0.15)	5.04 (0.10)	4.38 (0.13)	<.001*	−0.18	<.001*
11	Corynebacterium matruchotii	N	–	4.81 (0.07)	4.87 (0.17)	4.86 (0.10)	4.70 (0.12)	0.550	−0.04	0.171
12	Selenomonas noxia	N	–	4.81 (0.08)	4.63 (0.18)	4.69 (0.11)	5.06 (0.13)	**0.049**	0.10	**<.001**
13	Prevotella nigrescens	N	O	4.31 (0.10)	4.48 (0.21)	4.28 (0.15)	4.26 (0.18)	0.714	−0.03	0.370
14	Parvimonas micra	N	–	4.29 (0.08)	4.08 (0.17)	4.35 (0.11)	4.33 (0.12)	0.401	0.00	0.967
15	Rothia dentocariosa	U	–	4.28 (0.09)	4.46 (0.19)	4.38 (0.13)	4.04 (0.15)	0.113	−0.10	**0.008**
16	Fusobacterium sp._oral_taxon_203	N	–	4.23 (0.10)	4.08 (0.22)	4.36 (0.15)	4.16 (0.17)	0.497	−0.01	0.713
17	Streptococcus gordonii	N	Y	4.19 (0.08)	3.93 (0.18)	4.15 (0.12)	4.40 (0.13)	0.090	0.04	**0.039**
18	Granulicatella adiacens	N	–	4.15 (0.06)	4.23 (0.14)	4.11 (0.09)	4.15 (0.10)	0.749	0.00	0.991
19	Streptococcus intermedius	N	Y	3.94 (0.10)	4.33 (0.21)	3.86 (0.15)	3.81 (0.16)	0.119	−0.10	**0.016**
20	Actinomyces naeslundii	N	B	3.85 (0.06)	4.00 (0.14)	3.88 (0.09)	3.72 (0.11)	0.230	−0.10	0.008
21	TM7_[G-1] sp._oral_taxon_346	P	–	3.78 (0.09)	3.63 (0.19)	3.90 (0.12)	3.70 (0.16)	0.421	0.00	0.917
22	Haemophilus parainfluenzae	N	–	3.74 (0.09)	4.21 (0.19)	3.73 (0.13)	3.48 (0.15)	**0.014**	−0.10	**0.004**
23	Fusobacterium naviforme	N	–	3.58 (0.08)	3.61 (0.18)	3.62 (0.12)	3.52 (0.14)	0.852	−0.03	0.294
24	Dialister invisus	N	–	3.51 (0.08)	3.12 (0.16)	3.50 (0.11)	3.75 (0.13)	**0.013**	0.10	**0.003**
25	Capnocytophaga gingivalis	N	G	3.36 (0.08)	3.39 (0.18)	3.45 (0.11)	3.23 (0.13)	0.423	−0.04	0.172
26	Streptococcus cristatus	N	–	3.27 (0.07)	3.17 (0.16)	3.27 (0.11)	3.32 (0.13)	0.776	0.01	0.754
27	Gemella morbillorum	N	–	3.14 (0.09)	3.37 (0.20)	3.35 (0.13)	2.73 (0.16)	**0.004**	−0.11	<.001*
28	Streptococcus salivarius	U	–	3.02 (0.08)	3.05 (0.18)	2.79 (0.13)	3.29 (0.14)	**0.028**	0.10	0.090
29	TM7_[G-1] sp._oral_taxon_349	N	–	2.99 (0.10)	2.71 (0.22)	3.15 (0.14)	2.95 (0.18)	0.251	0.04	0.204
30	Bacteroidales_[G-2] sp._oral_taxon_274	N	–	2.96 (0.10)	2.47 (0.22)	3.15 (0.14)	3.00 (0.16)	**0.030**	0.03	0.328
31	Campylobacter showae	P	O	2.89 (0.08)	3.00 (0.17)	2.81 (0.11)	2.93 (0.14)	0.609	−0.01	0.685
32	Veillonella atypica	N	–	2.88 (0.09)	2.47 (0.20)	2.56 (0.13)	3.53 (0.14)	<.001*	0.16	<.001*
33	*Eikenella corrodens*	N	–	2.87 (0.07)	3.00 (0.15)	3.07 (0.10)	2.53 (0.12)	**0.001**	−0.11	<.001*
34	Capnocytophaga leadbetteri	N	–	2.86 (0.09)	2.87 (0.20)	2.93 (0.13)	2.78 (0.16)	0.758	−0.03	0.334
35	Fretibacterium sp._oral_taxon_360	N	–	2.81 (0.11)	2.29 (0.23)	2.89 (0.16)	3.00 (0.19)	**0.048**	0.04	0.148
36	Prevotella sp._oral_taxon_317	N	–	2.73 (0.10)	2.79 (0.22)	2.68 (0.16)	2.76 (0.18)	0.907	−0.00	0.965
37	Capnocytophaga granulosa	U	–	2.72 (0.09)	2.39 (0.21)	2.84 (0.13)	2.75 (0.16)	0.189	0.04	0.152
38	Alloprevotella tannerae	N	–	2.71 (0.12)	2.69 (0.24)	2.78 (0.17)	2.64 (0.20)	0.865	0.01	0.788
39	Kingella oralis	P	–	2.66 (0.08)	2.66 (0.19)	2.65 (0.11)	2.68 (0.14)	0.983	−0.01	0.847
40	TM7_[G-1] sp._oral_taxon_952	U	–	2.58 (0.10)	2.80 (0.22)	2.83 (0.14)	2.12 (0.16)	**0.002**	−0.10	**<.001**
41	Selenomonas artemidis	N	–	2.51 (0.11)	2.77 (0.24)	2.39 (0.16)	2.52 (0.19)	0.419	−0.00	0.957
42	Campylobacter concisus	N	G	2.41 (0.06)	2.22 (0.14)	2.37 (0.10)	2.56 (0.11)	0.152	0.10	**0.036**
43	Actinomyces oris	N	B	2.36 (0.08)	2.63 (0.17)	2.37 (0.11)	2.18 (0.13)	0.104	−0.10	**0.021**
44	Treponema socranskii	N	–	2.22 (0.07)	1.84 (0.17)	2.16 (0.11)	2.52 (0.13)	**0.004**	0.10	**<.001**
45	Cardiobacterium hominis	N	–	2.21 (0.08)	2.50 (0.19)	2.45 (0.12)	1.75 (0.14)	<.001*	−0.11	**<.001**
46	Leptotrichia wadei	N	–	2.15 (0.10)	2.21 (0.23)	1.94 (0.16)	2.38 (0.18)	0.159	0.03	0.305
47	Anaeroglobus geminatus	P	–	2.12 (0.10)	1.32 (0.22)	1.94 (0.15)	2.79 (0.18)	<.001*	0.17	<.001*
48	Gemella haemolysans	N	–	2.11 (0.09)	2.42 (0.20)	2.10 (0.13)	1.96 (0.14)	0.161	−0.10	**0.018**
49	Capnocytophaga sputigena	N	G	2.11 (0.09)	2.31 (0.21)	2.23 (0.13)	1.84 (0.17)	0.113	−0.10	0.094
50	Rothia aeria	N	–	2.11 (0.09)	2.49 (0.20)	2.42 (0.14)	1.48 (0.15)	<.001*	−0.15	<.001*
51	Bergeyella sp._oral_taxon_322	N	–	2.08 (0.07)	2.38 (0.16)	2.11 (0.10)	1.87 (0.11)	**0.028**	−0.10	**0.008**
52	Leptotrichia hongkongensis	N	–	2.02 (0.09)	2.02 (0.21)	1.94 (0.14)	2.13 (0.16)	0.660	0.03	0.337
53	Rothia mucilaginosa	U	–	1.95 (0.08)	1.79 (0.17)	1.90 (0.11)	2.09 (0.13)	0.321	0.03	0.237
54	Prevotella melaninogenica	N	–	1.90 (0.08)	1.75 (0.17)	1.78 (0.12)	2.14 (0.14)	0.095	0.10	**0.049**
55	Actinomyces sp._oral_taxon_169	N	–	1.89 (0.09)	2.28 (0.20)	1.96 (0.13)	1.59 (0.15)	**0.016**	−0.10	**<.001**
56	Tannerella forsythia	N	R	1.87 (0.10)	1.50 (0.21)	1.80 (0.14)	2.18 (0.17)	**0.042**	0.10	**0.029**
57	Selenomonas sp._oral_taxon_136	N	–	1.86 (0.08)	1.21 (0.18)	1.76 (0.12)	2.36 (0.15)	<.001*	0.16	<.001*
58	Catonella morbi	N	–	1.86 (0.08)	2.08 (0.17)	1.89 (0.11)	1.69 (0.13)	0.184	−0.10	**0.020**
59	Prevotella denticola	N	–	1.74 (0.10)	1.46 (0.23)	1.60 (0.15)	2.10 (0.18)	**0.040**	0.10	**0.006**
60	Neisseria sicca	N	–	1.72 (0.11)	1.58 (0.25)	1.88 (0.16)	1.60 (0.18)	0.416	−0.01	0.678
61	Peptostreptococcaceae_[XI][G-9] [Eubacterium]_brac	U	–	1.70 (0.08)	2.08 (0.16)	1.72 (0.12)	1.47 (0.14)	**0.030**	−0.10	**0.003**
62	Neisseria elongata	N	–	1.63 (0.10)	1.95 (0.25)	1.81 (0.15)	1.22 (0.17)	**0.012**	−0.10	**0.008**
63	Cardiobacterium valvarum	U	–	1.49 (0.09)	1.60 (0.21)	1.63 (0.13)	1.26 (0.14)	0.137	−0.10	**0.044**
64	Porphyromonas sp._oral_taxon_279	P	–	1.49 (0.09)	1.46 (0.20)	1.62 (0.13)	1.34 (0.16)	0.394	−0.02	0.494
65	Selenomonas infelix	U	–	1.48 (0.08)	1.56 (0.17)	1.44 (0.12)	1.50 (0.15)	0.871	−0.01	0.709
66	Leptotrichia sp._oral_taxon_212	U	–	1.44 (0.09)	1.63 (0.21)	1.64 (0.13)	1.08 (0.16)	**0.013**	−0.10	**0.011**
67	Actinomyces sp._oral_taxon_180	N	–	1.44 (0.07)	1.54 (0.15)	1.45 (0.10)	1.38 (0.11)	0.704	−0.03	0.235
68	Prevotella sp._oral_taxon_300	N	–	1.43 (0.09)	1.31 (0.19)	1.24 (0.13)	1.75 (0.15)	**0.025**	0.10	**0.007**
69	Prevotella maculosa	N	–	1.42 (0.08)	1.07 (0.17)	1.32 (0.11)	1.75 (0.13)	**0.003**	0.10	**0.001**
70	Streptococcus mutans	N	–	1.39 (0.11)	0.98 (0.24)	1.19 (0.16)	1.87 (0.20)	**0.005**	0.10	**<.001**
71	Actinomyces massiliensis	N	B	1.38 (0.07)	1.68 (0.17)	1.50 (0.10)	1.05 (0.11)	**0.002**	−0.12	<.001*
72	Fretibacterium fastidiosum	N	–	1.30 (0.10)	1.12 (0.22)	1.25 (0.15)	1.47 (0.17)	0.393	0.04	0.173
73	Veillonellaceae_[G-1] sp._oral_taxon_150	N	–	1.23 (0.09)	0.74 (0.20)	1.00 (0.13)	1.81 (0.16)	<.001*	0.14	<.001*
74	Neisseria flavescens	N	–	1.23 (0.10)	0.98 (0.21)	1.28 (0.14)	1.30 (0.17)	0.425	0.02	0.533
75	Prevotella oulorum	P	–	1.22 (0.09)	1.23 (0.20)	1.00 (0.13)	1.50 (0.15)	**0.041**	0.10	0.059
76	*Streptococcus anginosus*	N	Y	1.17 (0.11)	1.03 (0.23)	0.92 (0.16)	1.56 (0.19)	**0.024**	0.10	**0.013**
77	Lachnoanaerobaculum saburreum	N	–	1.07 (0.08)	0.85 (0.18)	1.12 (0.12)	1.13 (0.14)	0.384	0.04	0.208
78	Veillonellaceae_[G-1] sp._oral_taxon_155	N	–	1.00 (0.09)	0.66 (0.20)	0.84 (0.13)	1.39 (0.15)	**0.004**	0.10	**<.001**
79	Selenomonas sp._oral_taxon_892	L	–	0.98 (0.08)	1.18 (0.19)	1.05 (0.12)	0.76 (0.15)	0.148	−0.10	0.030
80	Actinomyces johnsonii	N	B	0.97 (0.07)	1.18 (0.16)	0.92 (0.11)	0.92 (0.12)	0.365	−0.04	0.229
81	Selenomonas sp._oral_taxon_137	U	–	0.95 (0.11)	0.95 (0.25)	1.21 (0.17)	0.61 (0.20)	0.064	−0.04	0.145
82	Corynebacterium durum	U	–	0.93 (0.08)	1.53 (0.19)	1.07 (0.12)	0.42 (0.13)	<.001*	−0.15	<.001*
83	Lautropia mirabilis	U	–	0.88 (0.09)	1.28 (0.21)	0.99 (0.13)	0.53 (0.15)	**0.006**	−0.10	**<.001**
84	Veillonella rogosae	N	–	0.87 (0.09)	1.06 (0.20)	1.08 (0.13)	0.49 (0.16)	**0.010**	−0.10	**<.001**
85	Leptotrichia sp._oral_taxon_417	N	–	0.86 (0.09)	0.41 (0.20)	0.95 (0.13)	1.01 (0.16)	**0.048**	0.10	**0.006**
86	Selenomonas flueggei	N	–	0.81 (0.08)	0.58 (0.18)	0.81 (0.12)	0.93 (0.14)	0.317	0.04	0.122
87	Selenomonas sp._oral_taxon_134	P	–	0.77 (0.10)	0.55 (0.21)	0.75 (0.14)	0.94 (0.18)	0.367	0.03	0.251
88	Streptococcus parasanguinis_II	U	–	0.74 (0.09)	0.58 (0.19)	0.49 (0.13)	1.15 (0.15)	**0.003**	0.11	<.001*
89	Megasphaera micronuciformis	N	–	0.70 (0.08)	0.47 (0.19)	0.46 (0.13)	1.14 (0.14)	**<.001**	0.12	<.001*
90	Capnocytophaga sp._oral_taxon_336	N	–	0.69 (0.09)	0.68 (0.19)	0.69 (0.13)	0.71 (0.16)	0.991	0.02	0.480
91	Oribacterium sp._oral_taxon_078	U	–	0.66 (0.08)	−0.03 (0.17)	0.51 (0.11)	1.26 (0.14)	<.001*	0.18	<.001*
92	Actinomyces sp._oral_taxon_171	P	–	0.66 (0.08)	0.82 (0.18)	0.66 (0.12)	0.57 (0.13)	0.527	−0.10	0.090
93	Lachnospiraceae_[G-3] sp._oral_taxon_100	U	–	0.59 (0.08)	0.60 (0.18)	0.73 (0.12)	0.41 (0.14)	0.200	−0.03	0.239
94	Parvimonas sp._oral_taxon_393	U	–	0.58 (0.11)	1.11 (0.24)	0.63 (0.16)	0.22 (0.17)	**0.010**	−0.12	<.001*
95	Actinomyces gerencseriae	N	B	0.56 (0.07)	0.49 (0.17)	0.37 (0.11)	0.86 (0.13)	0.012	0.10	**0.008**
96	Dialister pneumosintes	P	–	0.55 (0.09)	0.64 (0.20)	0.42 (0.14)	0.65 (0.16)	0.477	0.03	0.357
97	Kingella denitrificans	N	–	0.54 (0.09)	0.21 (0.20)	0.45 (0.13)	0.84 (0.15)	**0.025**	0.10	**0.024**
98	Porphyromonas endodontalis	N	–	0.52 (0.12)	1.09 (0.27)	0.56 (0.17)	0.13 (0.20)	**0.015**	−0.10	**<.001**
99	TM7_[G-5] sp._oral_taxon_356	P	–	0.49 (0.11)	0.30 (0.23)	0.61 (0.16)	0.43 (0.19)	0.517	0.02	0.580
100	Selenomonas sp._oral_taxon_919	U	–	0.47 (0.08)	0.72 (0.18)	0.45 (0.12)	0.35 (0.15)	0.287	−0.10	0.096
101	Leptotrichia buccalis	N	–	0.45 (0.10)	0.23 (0.23)	0.77 (0.14)	0.16 (0.16)	**0.011**	−0.03	0.375
102	Prevotella sp._oral_taxon_472	N	–	0.45 (0.10)	0.65 (0.23)	0.80 (0.15)	−0.13 (0.17)	<.001*	−0.10	**<.001**
103	Capnocytophaga sp._oral_taxon_326	P	–	0.41 (0.10)	0.38 (0.23)	0.62 (0.15)	0.15 (0.17)	0.121	−0.10	0.080
104	Streptococcus constellatus	N	O	0.35 (0.10)	0.25 (0.21)	0.18 (0.15)	0.63 (0.17)	0.114	0.04	0.139
105	Prevotella salivae	N	–	0.34 (0.08)	−0.22 (0.18)	0.32 (0.12)	0.69 (0.14)	**<.001**	0.15	<.001*
106	Leptotrichia sp._oral_taxon_392	U	–	0.33 (0.09)	0.49 (0.20)	0.58 (0.13)	− 0.08 (0.15)	**0.003**	−0.10	**0.001**
107	Actinobaculum sp._oral_taxon_183	N	–	0.32 (0.08)	0.29 (0.17)	0.28 (0.12)	0.40 (0.14)	0.765	0.01	0.742
108	Neisseria oralis	U	–	0.31 (0.11)	0.47 (0.25)	0.35 (0.15)	0.19 (0.18)	0.618	−0.04	0.133
109	Atopobium rimae	N	–	0.21 (0.09)	−0.08 (0.19)	0.17 (0.13)	0.43 (0.16)	0.124	0.10	0.108
110	Leptotrichia hofstadii	N	–	0.20 (0.10)	0.10 (0.22)	0.27 (0.15)	0.17 (0.17)	0.804	0.01	0.743
111	Streptococcus sp._oral_taxon_074	N	–	0.15 (0.07)	0.15 (0.16)	0.25 (0.11)	0.02 (0.12)	0.340	−0.04	0.154
112	Fretibacterium sp._oral_taxon_359	L	–	0.15 (0.11)	0.14 (0.25)	0.04 (0.16)	0.30 (0.18)	0.537	0.02	0.552
113	Leptotrichia shahii	U	–	0.11 (0.10)	0.30 (0.22)	−0.09 (0.15)	0.26 (0.19)	0.209	0.03	0.298
114	Tannerella sp._oral_taxon_286	U	–	0.11 (0.07)	− 0.15 (0.16)	0.26 (0.10)	0.06 (0.13)	0.101	0.03	0.370
115	Porphyromonas sp._oral_taxon_284	N	–	0.08 (0.10)	0.14 (0.21)	0.22 (0.14)	− 0.14 (0.16)	0.223	−0.10	0.080
116	Peptostreptococcaceae_[XI][G-7] [Eubacterium]_yuri	P	–	0.05 (0.10)	0.50 (0.21)	0.19 (0.14)	−0.40 (0.16)	**0.001**	−0.14	<.001*
117	Selenomonas sp._oral_taxon_146	L	–	0.04 (0.08)	0.04 (0.18)	− 0.03 (0.12)	0.14 (0.14)	0.659	0.014	0.633
118	Fusobacterium periodonticum	N	O	−0.02 (0.07)	−0.12 (0.16)	0.13 (0.11)	−0.17 (0.13)	0.174	−0.02	0.488
119	Actinomyces meyeri	L	B	−0.04 (0.08)	0.29 (0.18)	0.05 (0.12)	− 0.35 (0.13)	**0.010**	−0.11	<.001*
120	Leptotrichia sp._oral_taxon_215	N	–	− 0.05 (0.08)	0.16 (0.18)	0.01 (0.12)	− 0.24 (0.14)	0.157	−0.04	0.170
121	Prevotella oralis	N	–	− 0.06 (0.10)	−0.34 (0.20)	− 0.13 (0.14)	0.20 (0.17)	0.105	0.10	**0.030**
122	Prevotella pleuritidis	N	–	− 0.10 (0.12)	−0.07 (0.27)	0.09 (0.18)	−0.38 (0.20)	0.211	−0.04	0.190
123	Abiotrophia defectiva	N	–	−0.15 (0.09)	−0.05 (0.19)	− 0.01 (0.13)	− 0.38 (0.14)	0.138	− 0.10	**0.028**
124	Gemella sanguinis	N	–	−0.19 (0.07)	−0.16 (0.15)	− 0.35 (0.10)	− 0.00 (0.12)	0.077	0.04	0.204
125	Fusobacterium nucleatum_subsp._nucleatum	N	O	−0.25 (0.08)	−0.44 (0.17)	− 0.30 (0.11)	− 0.08 (0.14)	0.209	0.03	0.259
126	Atopobium parvulum	N	–	− 0.25 (0.08)	−0.28 (0.17)	− 0.47 (0.11)	0.06 (0.14)	**0.009**	0.10	**0.005**
127	Aggregatibacter sp._oral_taxon_458	N	–	− 0.25 (0.09)	− 0.11 (0.19)	− 0.17 (0.13)	− 0.44 (0.15)	0.309	−0.10	**0.047**
128	Streptococcus parasanguinis_I	N	–	− 0.26 (0.08)	−0.29 (0.17)	− 0.48 (0.11)	0.05 (0.13)	**0.008**	0.10	**0.008**
129	Aggregatibacter aphrophilus	N	–	− 0.27 (0.11)	−0.18 (0.25)	− 0.01 (0.17)	− 0.66 (0.18)	**0.027**	− 0.10	**0.015**
130	Treponema denticola	N	R	−0.28 (0.10)	−0.16 (0.22)	− 0.25 (0.15)	− 0.39 (0.18)	0.692	− 0.04	0.211
131	Prevotella saccharolytica	U	–	− 0.37 (0.07)	− 0.38 (0.16)	− 0.26 (0.11)	− 0.51 (0.13)	0.336	− 0.01	0.663
132	TM7_[G-1] sp._oral_taxon_488	P	–	− 0.39 (0.10)	− 0.39 (0.22)	− 0.11 (0.15)	− 0.76 (0.16)	**0.015**	−0.04	0.132
133	Johnsonella ignava	U	–	− 0.41 (0.09)	−0.50 (0.21)	− 0.34 (0.14)	− 0.45 (0.16)	0.783	− 0.01	0.783
134	Actinomyces israelii	N	B	−0.47 (0.06)	−0.65 (0.13)	− 0.52 (0.09)	− 0.30 (0.11)	0.091	0.10	**0.012**
135	Lachnoanaerobaculum umeaense	P	–	− 0.47 (0.07)	−0.35 (0.17)	− 0.39 (0.11)	− 0.65 (0.12)	0.198	−0.03	0.316
136	Streptococcus sp._oral_taxon_056	U	–	− 0.49 (0.08)	−0.38 (0.19)	− 0.37 (0.12)	− 0.73 (0.14)	0.127	−0.04	0.050
137	Olsenella sp._oral_taxon_807	N	–	− 0.49 (0.07)	−0.93 (0.15)	− 0.50 (0.10)	− 0.25 (0.12)	**0.002**	0.11	<.001*
138	Solobacterium moorei	U	–	− 0.51 (0.06)	−0.49 (0.15)	− 0.60 (0.09)	− 0.42 (0.11)	0.437	0.04	0.221
139	Treponema maltophilum	P	–	− 0.53 (0.08)	−0.78 (0.16)	− 0.59 (0.11)	− 0.32 (0.13)	0.070	0.10	**0.040**
140	TM7_[G-1] sp._oral_taxon_348	N	–	− 0.53 (0.08)	− 0.66 (0.17)	− 0.29 (0.13)	− 0.77 (0.14)	**0.026**	− 0.03	0.373
141	Porphyromonas gingivalis	N	R	−0.56 (0.12)	−0.70 (0.26)	− 0.60 (0.17)	− 0.43 (0.22)	0.688	0.03	0.348
142	Centipeda periodontii	N	–	− 0.60 (0.08)	− 0.94 (0.15)	− 0.55 (0.11)	− 0.47 (0.13)	0.074	0.06	**0.031**
143	Selenomonas sp._oral_taxon_126	N	–	− 0.60 (0.08)	−0.77 (0.18)	− 0.56 (0.12)	− 0.56 (0.14)	0.588	0.03	0.386
144	Desulfobulbus sp._oral_taxon_041	U	–	−0.66 (0.10)	−1.02 (0.20)	− 0.70 (0.14)	− 0.40 (0.17)	0.064	0.10	**0.027**
145	Alloprevotella rava	U	–	−0.68 (0.08)	−0.78 (0.18)	− 0.64 (0.12)	− 0.69 (0.14)	0.809	0.02	0.464
146	Actinobaculum sp._oral_taxon_848	P	–	− 0.70 (0.08)	−0.83 (0.17)	− 0.82 (0.11)	−0.46 (0.13)	0.080	0.10	**0.016**
147	Stomatobaculum longum	N	–	− 0.70 (0.07)	− 0.95 (0.16)	− 0.76 (0.11)	−0.48 (0.13)	0.054	0.11	**<.001**
148	Prevotella histicola	N	–	− 0.75 (0.09)	− 0.96 (0.18)	−1.04 (0.13)	− 0.26 (0.16)	**<.001**	0.11	<.001*
149	Aggregatibacter segnis	U	–	− 0.79 (0.10)	− 0.42 (0.22)	− 0.69 (0.14)	−1.13 (0.15)	**0.020**	− 0.11	<.001*
150	Leptotrichia sp._oral_taxon_225	N	–	− 0.80 (0.10)	− 0.43 (0.22)	− 0.60 (0.15)	− 1.25 (0.15)	**0.002**	− 0.11	<.001*
151	Ruminococcaceae_[G-1] sp._oral_taxon_075	N	–	− 0.80 (0.08)	− 0.79 (0.17)	− 0.69 (0.12)	−0.94 (0.13)	0.350	−0.03	0.341
152	Peptostreptococcus stomatis	U	–	− 0.80 (0.09)	−0.50 (0.19)	− 0.77 (0.13)	− 1.02 (0.14)	0.085	− 0.10	**0.032**
153	Porphyromonas catoniae	P	–	− 0.92 (0.09)	− 0.50 (0.20)	− 0.79 (0.13)	−1.31 (0.14)	**0.002**	− 0.10	**<.001**
154	Actinomyces sp._oral_taxon_178	P	–	−0.93 (0.06)	− 1.15 (0.14)	− 0.94 (0.09)	− 0.78 (0.10)	0.099	0.05	0.080
155	Prevotella pallens	N	–	− 0.93 (0.08)	−1.00 (0.17)	− 1.05 (0.11)	−0.73 (0.14)	0.191	0.10	0.054
156	Filifactor alocis	U	–	− 0.94 (0.10)	−0.66 (0.23)	− 0.98 (0.15)	− 1.05 (0.18)	0.382	− 0.10	0.077
157	Selenomonas sp._oral_taxon_936	P	–	−0.96 (0.08)	− 0.91 (0.17)	−1.05 (0.12)	− 0.87 (0.14)	0.578	0.03	0.323
158	TM7_[G-1] sp._oral_taxon_347	N	–	− 0.97 (0.09)	−0.64 (0.20)	− 0.95 (0.14)	−1.17 (0.14)	0.107	− 0.10	0.071
159	Haemophilus sp._oral_taxon_036	N	–	−1.01 (0.09)	− 0.65 (0.19)	− 1.00 (0.13)	− 1.21 (0.15)	0.075	− 0.10	**0.002**
160	Prevotella sp._oral_taxon_292	P	–	− 1.06 (0.08)	− 1.34 (0.17)	− 1.15 (0.12)	−0.79 (0.15)	**0.040**	0.10	**0.001**
161	Leptotrichia sp._oral_taxon_498	N	–	− 1.07 (0.09)	−1.28 (0.20)	− 1.20 (0.13)	−0.77 (0.16)	0.059	0.10	**0.013**
162	Capnocytophaga sp._oral_taxon_338	U	–	− 1.08 (0.09)	−1.17 (0.20)	− 1.12 (0.13)	−0.98 (0.15)	0.671	0.01	0.808
163	Alloprevotella sp._oral_taxon_308	N	–	− 1.14 (0.07)	−0.90 (0.14)	−1.28 (0.10)	− 1.10 (0.12)	0.111	−0.01	0.799
164	Treponema sp._oral_taxon_231	P	–	− 1.15 (0.09)	−1.04 (0.18)	− 1.03 (0.13)	− 1.36 (0.15)	0.206	− 0.10	0.074
165	TM7_[G-1] sp._oral_taxon_352	N	–	− 1.15 (0.07)	− 1.31 (0.15)	− 1.11 (0.10)	− 1.11 (0.12)	0.545	0.04	0.173
166	Prevotella buccae	U	–	− 1.23 (0.07)	−1.71 (0.14)	− 1.36 (0.11)	− 0.78 (0.12)	<.001*	0.14	<.001*
167	Prevotella loescheii	N	–	−1.24 (0.09)	−0.96 (0.21)	−0.97 (0.14)	− 1.74 (0.15)	**<.001**	− 0.10	**0.001**
168	Capnocytophaga sp._oral_taxon_864	U	–	− 1.27 (0.08)	−0.98 (0.20)	−1.15 (0.13)	− 1.57 (0.13)	**0.020**	− 0.10	**0.005**
169	Actinomyces sp._oral_taxon_170	U	–	−1.29 (0.08)	− 0.99 (0.19)	− 1.16 (0.13)	− 1.62 (0.13)	**0.011**	− 0.10	**<.001**
170	Lachnoanaerobaculum orale	U	–	− 1.31 (0.07)	− 1.09 (0.16)	− 1.46 (0.09)	−1.23 (0.12)	0.085	0.02	0.513
171	Ottowia sp._oral_taxon_894	N	–	− 1.31 (0.08)	−1.44 (0.18)	− 1.24 (0.12)	− 1.33 (0.14)	0.662	−0.00	0.938
172	Streptococcus lactarius	N	–	− 1.32 (0.07)	− 1.06 (0.15)	− 1.46 (0.10)	− 1.30 (0.12)	0.097	−0.02	0.414
173	Peptostreptococcaceae_[XI][G-1] [Eubacterium]_infi	P	–	− 1.33 (0.07)	− 1.59 (0.15)	− 1.41 (0.09)	− 1.08 (0.12)	**0.014**	0.10	**0.002**
174	Leptotrichia sp._oral_taxon_219	N	–	− 1.34 (0.07)	−1.39 (0.17)	− 1.27 (0.11)	−1.39 (0.12)	0.736	−0.01	0.717
175	Prevotella intermedia	P	O	−1.36 (0.11)	−1.31 (0.25)	− 1.35 (0.17)	− 1.41 (0.18)	0.943	−0.04	0.181
176	Veillonella denticariosi	U	–	− 1.37 (0.09)	− 1.51 (0.19)	− 1.39 (0.13)	− 1.27 (0.15)	0.621	0.04	0.156
177	Fretibacterium sp._oral_taxon_362	N	–	− 1.43 (0.08)	−1.48 (0.19)	− 1.43 (0.12)	− 1.42 (0.14)	0.965	0.00	0.888
178	Bacteroidetes_[G-5] sp._oral_taxon_511	U	–	− 1.47 (0.09)	− 1.44 (0.19)	− 1.43 (0.14)	− 1.54 (0.15)	0.846	−0.03	0.378
179	Prevotella sp._oral_taxon_313	P	–	− 1.49 (0.08)	−1.14 (0.18)	− 1.75 (0.11)	− 1.35 (0.14)	**0.007**	0.02	0.431
180	TM7_[G-3] sp._oral_taxon_351	P	–	− 1.50 (0.06)	−1.84 (0.13)	− 1.39 (0.10)	− 1.45 (0.11)	**0.029**	0.10	**0.018**
181	Streptococcus australis	N	–	− 1.51 (0.07)	−1.62 (0.15)	− 1.57 (0.11)	− 1.38 (0.13)	0.373	0.03	0.295
182	Granulicatella elegans	N	–	− 1.59 (0.07)	−1.18 (0.17)	− 1.72 (0.11)	−1.65 (0.12)	**0.023**	−0.10	0.067
183	TM7_[G-1] sp._oral_taxon_869	P	–	− 1.60 (0.09)	−1.64 (0.20)	− 1.55 (0.14)	− 1.64 (0.15)	0.890	0.01	0.663
184	Capnocytophaga sp._oral_taxon_902	N	–	−1.65 (0.08)	− 1.56 (0.19)	− 1.68 (0.12)	−1.67 (0.14)	0.860	−0.04	0.220
185	Prevotella dentalis	P	–	−1.66 (0.08)	−1.65 (0.19)	− 1.82 (0.12)	−1.47 (0.14)	0.180	0.03	0.326
186	Campylobacter curvus	N	–	− 1.67 (0.07)	−1.57 (0.17)	− 1.79 (0.10)	− 1.58 (0.13)	0.365	0.03	0.335
187	Pseudoramibacter alactolyticus	P	–	− 1.68 (0.08)	− 2.18 (0.17)	−1.73 (0.12)	− 1.35 (0.15)	**0.001**	0.12	<.001*
188	TM7_[G-6] sp._oral_taxon_870	N	–	− 1.71 (0.08)	−1.47 (0.18)	− 1.69 (0.12)	− 1.85 (0.13)	0.220	−0.10	0.085
189	Veillonellaceae_[G-1] sp._oral_taxon_129	N	–	− 1.71 (0.08)	− 1.96 (0.16)	− 1.80 (0.11)	− 1.46 (0.14)	**0.042**	0.10	**0.010**
190	Tannerella sp._oral_taxon_808	U	–	− 1.75 (0.07)	−2.03 (0.14)	−1.70 (0.10)	− 1.65 (0.12)	0.104	0.10	**0.011**
191	Peptostreptococcaceae_[XI][G-6] [Eubacterium]_noda	L	–	− 1.75 (0.08)	− 1.67 (0.18)	− 1.90 (0.11)	−1.61 (0.14)	0.238	0.02	0.425
192	Mitsuokella sp._oral_taxon_131	N	–	− 1.79 (0.09)	−2.18 (0.17)	− 1.96 (0.13)	− 1.36 (0.16)	**<.001**	0.14	<.001*
193	Mycoplasma salivarium	P	–	− 1.80 (0.07)	−1.77 (0.15)	− 1.87 (0.11)	− 1.71 (0.12)	0.579	0.03	0.302
194	Haemophilus haemolyticus	P	–	− 1.81 (0.08)	−1.30 (0.19)	− 1.92 (0.12)	− 1.94 (0.12)	**0.006**	−0.10	**0.043**
195	Bergeyella sp._oral_taxon_907	P	–	−1.81 (0.07)	−1.86 (0.15)	− 1.72 (0.10)	− 1.92 (0.12)	0.405	− 0.01	0.765
196	TM7_[G-2] sp._oral_taxon_350	N	–	− 1.82 (0.09)	− 1.86 (0.18)	− 1.82 (0.13)	− 1.80 (0.15)	0.968	0.02	0.412
197	Prevotella veroralis	U	–	− 1.82 (0.08)	−1.73 (0.18)	− 1.78 (0.13)	− 1.93 (0.13)	0.582	−0.02	0.413
198	Aggregatibacter paraphrophilus	N	–	− 1.82 (0.09)	−1.48 (0.21)	− 1.69 (0.14)	−2.19 (0.13)	**0.007**	−0.10	**<.001**
199	Leptotrichia sp._oral_taxon_223	P	–	− 1.82 (0.08)	−1.71 (0.17)	− 1.98 (0.11)	− 1.69 (0.14)	0.206	0.02	0.499
200	Bifidobacterium dentium	N	–	− 1.85 (0.08)	−2.10 (0.18)	−2.12 (0.12)	−1.37 (0.15)	<.001*	0.15	<.001*
201	Alloprevotella sp._oral_taxon_473	U	–	− 1.87 (0.08)	−1.63 (0.17)	− 1.88 (0.12)	− 1.98 (0.13)	0.300	−0.10	**0.030**
202	Veillonellaceae_[G-1] sp._oral_taxon_145	U	–	− 1.88 (0.08)	−1.98 (0.16)	− 1.83 (0.12)	− 1.88 (0.14)	0.768	0.02	0.551
203	Neisseria bacilliformis	N	–	−1.89 (0.09)	−1.82 (0.20)	− 1.90 (0.13)	− 1.93 (0.14)	0.906	−0.04	0.194
204	Capnocytophaga sp._oral_taxon_412	N	–	− 1.89 (0.08)	−1.74 (0.18)	− 1.81 (0.12)	−2.09 (0.13)	0.173	− 0.04	0.162
205	Neisseria subflava	N	–	− 1.89 (0.09)	− 1.84 (0.18)	−2.04 (0.13)	− 1.73 (0.16)	0.267	0.10	0.118
206	Veillonella sp._oral_taxon_780	N	–	− 1.94 (0.08)	− 1.46 (0.20)	− 1.93 (0.12)	− 2.20 (0.12)	**0.004**	−0.10	**<.001**
207	Lachnospiraceae_[G-8] sp._oral_taxon_500	N	–	− 1.94 (0.07)	−1.91 (0.16)	−2.03 (0.10)	− 1.83 (0.12)	0.443	0.01	0.695
208	Capnocytophaga sp._oral_taxon_323	P	–	−1.94 (0.08)	−1.99 (0.17)	− 1.93 (0.12)	− 1.94 (0.14)	0.966	− 0.01	0.731
209	Scardovia wiggsiae	P	–	−1.94 (0.09)	− 2.07 (0.19)	− 2.11 (0.12)	− 1.66 (0.16)	0.061	0.10	**0.009**
210	Sphingomonas echinoides	N	–	− 1.95 (0.06)	−1.75 (0.14)	− 1.89 (0.10)	− 2.15 (0.11)	0.056	−0.10	**<.001**
211	SR1_[G-1] sp._oral_taxon_874	U	–	− 1.99 (0.07)	−1.76 (0.15)	− 1.88 (0.10)	−2.26 (0.11)	**0.008**	−0.10	**0.006**
212	Treponema lecithinolyticum	U	–	−2.01 (0.08)	−1.98 (0.17)	− 1.90 (0.12)	− 2.18 (0.13)	0.302	−0.10	0.068
213	Prevotella baroniae	P	–	−2.04 (0.07)	−2.20 (0.15)	− 2.03 (0.11)	− 1.96 (0.13)	0.490	0.04	0.209
214	*Pseudomonas fluorescens*	P	–	− 2.05 (0.07)	− 2.12 (0.15)	− 2.07 (0.11)	−1.98 (0.13)	0.767	0.03	0.334
215	Shuttleworthia satelles	P	–	− 2.08 (0.07)	− 2.27 (0.14)	− 2.21 (0.10)	−1.82 (0.12)	**0.016**	0.10	**<.001**
216	Prevotella sp._oral_taxon_306	U		−2.08 (0.07)	−2.32 (0.14)	− 2.21 (0.10)	−1.79 (0.13)	**0.010**	0.10	**<.001**
217	Porphyromonas sp._oral_taxon_275	N	–	− 2.11 (0.07)	−2.09 (0.16)	− 2.01 (0.12)	− 2.26 (0.12)	0.321	−0.04	0.184
218	Streptococcus sinensis	P	–	− 2.14 (0.06)	− 1.95 (0.13)	− 2.30 (0.08)	− 2.05 (0.11)	0.050	0.02	0.517
219	Anaerolineae_[G-1] sp._oral_taxon_439	N	–	− 2.14 (0.08)	− 2.47 (0.15)	− 2.22 (0.11)	−1.85 (0.14)	**0.008**	0.12	<.001*
220	Bacteroidaceae_[G-1] sp._oral_taxon_272	N	–	− 2.15 (0.07)	− 2.48 (0.15)	− 2.21 (0.10)	−1.87 (0.13)	**0.007**	0.10	**<.001**
221	Prevotella micans	N	–	− 2.15 (0.07)	−2.24 (0.14)	− 2.18 (0.10)	− 2.08 (0.12)	0.658	0.02	0.452
222	Leptotrichia goodfellowii	U	–	− 2.17 (0.07)	−1.90 (0.17)	−2.14 (0.11)	− 2.36 (0.12)	0.080	−0.10	**0.008**
223	Capnocytophaga sp._oral_taxon_903	N	–	− 2.17 (0.08)	−2.15 (0.17)	− 2.12 (0.11)	− 2.25 (0.13)	0.751	− 0.01	0.731
224	Peptostreptococcaceae_[XI][G-5] [Eubacterium]_saph	U	–	− 2.18 (0.08)	−2.46 (0.15)	− 2.13 (0.12)	− 2.08 (0.13)	0.195	0.04	0.229
225	Treponema sp._oral_taxon_237	P	–	− 2.20 (0.08)	−1.68 (0.19)	−2.21 (0.12)	− 2.46 (0.13)	**0.003**	−0.11	<.001*
226	Fusobacterium sp._oral_taxon_370	U	–	− 2.20 (0.06)	−2.25 (0.12)	− 2.08 (0.09)	− 2.32 (0.10)	0.176	−0.04	0.176
227	Atopobium sp._oral_taxon_199	U	–	− 2.26 (0.07)	−2.19 (0.16)	− 2.18 (0.11)	− 2.40 (0.12)	0.334	−0.02	0.433
228	Prevotella sp._oral_taxon_314	N	–	− 2.28 (0.07)	− 2.29 (0.16)	− 2.35 (0.11)	− 2.18 (0.13)	0.605	0.02	0.519
229	Capnocytophaga sp._oral_taxon_324	U	–	− 2.28 (0.07)	−2.43 (0.15)	− 2.24 (0.11)	− 2.25 (0.12)	0.588	0.03	0.256
230	Haemophilus parahaemolyticus	N	–	− 2.29 (0.08)	−1.86 (0.18)	−2.23 (0.12)	− 2.61 (0.11)	**0.002**	−0.11	<.001*
231	Porphyromonas sp._oral_taxon_278	U	–	− 2.29 (0.07)	−2.40 (0.14)	− 2.24 (0.11)	− 2.30 (0.12)	0.716	0.00	0.977
232	Selenomonas sp._oral_taxon_937	U	–	− 2.35 (0.06)	−2.34 (0.12)	− 2.46 (0.09)	− 2.22 (0.11)	0.203	0.03	0.386
233	Selenomonas dianae	N	–	− 2.36 (0.07)	− 2.52 (0.14)	− 2.27 (0.10)	− 2.38 (0.11)	0.367	0.02	0.569
234	Selenomonas sp._oral_taxon_133	U	–	− 2.37 (0.08)	− 2.25 (0.18)	− 2.40 (0.11)	− 2.41 (0.13)	0.719	−0.02	0.396
235	Selenomonas sp._oral_taxon_478	P	–	− 2.49 (0.06)	−2.54 (0.13)	− 2.46 (0.09)	− 2.49 (0.10)	0.882	0.03	0.306
236	Capnocytophaga sp._oral_taxon_332	U	–	− 2.50 (0.07)	−2.26 (0.16)	− 2.42 (0.11)	− 2.72 (0.11)	**0.043**	−0.10	**0.043**
237	Megasphaera sp._oral_taxon_123	N	–	− 2.51 (0.08)	− 2.06 (0.19)	− 2.51 (0.12)	− 2.77 (0.13)	**0.009**	− 0.10	**0.002**
238	Bradyrhizobium elkanii	U	–	− 2.54 (0.08)	−2.37 (0.17)	− 2.68 (0.11)	− 2.44 (0.13)	0.209	−0.00	0.900
239	Prevotella sp._oral_taxon_376	P	–	− 2.54 (0.07)	−2.22 (0.17)	− 2.49 (0.11)	− 2.79 (0.12)	**0.015**	−0.10	**<.001**
240	Prevotella sp._oral_taxon_526	P	–	− 2.56 (0.07)	−2.59 (0.14)	− 2.57 (0.10)	− 2.52 (0.12)	0.923	0.01	0.736
241	Selenomonas sp._oral_taxon_442	U	–	− 2.58 (0.05)	− 2.78 (0.11)	− 2.52 (0.09)	− 2.53 (0.09)	0.177	0.10	0.065
242	Leptotrichia sp._oral_taxon_879	U	–	− 2.60 (0.07)	− 2.56 (0.16)	− 2.62 (0.10)	−2.59 (0.11)	0.951	− 0.02	0.597
243	Selenomonas sp._oral_taxon_149	P	–	− 2.61 (0.06)	− 2.75 (0.12)	− 2.70 (0.09)	− 2.42 (0.10)	**0.048**	0.10	**0.004**
244	Aggregatibacter sp._oral_taxon_513	U	–	− 2.66 (0.07)	− 2.64 (0.14)	− 2.52 (0.11)	− 2.85 (0.11)	0.107	− 0.03	0.368
245	Treponema vincentii	N	–	− 2.67 (0.06)	−2.55 (0.13)	− 2.60 (0.09)	− 2.83 (0.09)	0.140	−0.04	0.125
246	Capnocytophaga sp._oral_taxon_380	N	–	− 2.70 (0.07)	−2.65 (0.14)	− 2.59 (0.11)	− 2.88 (0.11)	0.132	−0.10	0.107
247	Johnsonella sp._oral_taxon_166	U	–	− 2.78 (0.06)	−2.96 (0.12)	− 2.69 (0.10)	− 2.80 (0.11)	0.292	−0.01	0.855
248	Treponema medium	P	–	−2.79 (0.06)	− 2.85 (0.14)	− 2.74 (0.10)	−2.81 (0.10)	0.777	0.02	0.579
249	Fretibacterium sp._oral_taxon_358	U	–	− 2.84 (0.07)	−2.87 (0.14)	− 2.88 (0.10)	− 2.77 (0.12)	0.708	0.02	0.494
250	Neisseria pharyngis	N	–	− 2.87 (0.06)	−2.81 (0.13)	− 2.95 (0.09)	− 2.80 (0.11)	0.479	0.01	0.760
251	Prevotella sp._oral_taxon_475	N	–	− 2.87 (0.06)	−2.81 (0.12)	− 2.84 (0.09)	− 2.95 (0.09)	0.575	−0.04	0.157
252	Microbacterium flavescens	P	–	− 2.89 (0.04)	− 2.73 (0.09)	− 2.89 (0.06)	−2.97 (0.07)	0.151	−0.10	0.051
253	Mitsuokella sp._oral_taxon_521	N	–	− 2.90 (0.06)	−2.91 (0.12)	− 2.92 (0.08)	− 2.87 (0.11)	0.929	0.02	0.603
254	Lactobacillus gasseri	N	–	− 2.95 (0.06)	−3.08 (0.12)	− 3.09 (0.09)	−2.70 (0.12)	**0.012**	0.10	**0.002**
255	Butyrivibrio sp._oral_taxon_080	P	–	− 2.96 (0.06)	−2.85 (0.13)	− 2.90 (0.09)	−3.10 (0.09)	0.193	−0.10	**0.038**
256	Fretibacterium sp._oral_taxon_361	P	–	− 2.98 (0.06)	− 3.18 (0.10)	− 2.97 (0.09)	− 2.88 (0.11)	0.207	0.02	0.469
257	Aggregatibacter actinomycetemcomitans	P	–	− 3.01 (0.07)	−2.97 (0.15)	−3.09 (0.10)	−2.94 (0.11)	0.575	0.03	0.349
258	Streptococcus sobrinus	U	–	− 3.04 (0.06)	−3.21 (0.10)	− 3.19 (0.09)	−2.75 (0.14)	**0.005**	0.11	<.001*
259	GN02_[G-2] sp._oral_taxon_873	P	–	− 3.06 (0.05)	−3.08 (0.12)	− 3.03 (0.08)	− 3.10 (0.09)	0.851	−0.00	0.927
260	Prevotella multiformis	N	–	− 3.08 (0.06)	− 3.12 (0.11)	− 3.10 (0.09)	− 3.05 (0.10)	0.901	0.03	0.359
261	Brevundimonas diminuta	N	–	− 3.30 (0.04)	− 3.17 (0.09)	− 3.35 (0.06)	− 3.30 (0.07)	0.232	−0.03	0.306
262	Atopobium sp._oral_taxon_416	P	–	− 3.38 (0.05)	− 3.45 (0.09)	− 3.44 (0.07)	− 3.27 (0.09)	0.202	0.10	**0.042**
263	Treponema sp._oral_taxon_247	P	–	− 3.45 (0.05)	− 3.35 (0.09)	− 3.50 (0.07)	− 3.44 (0.08)	0.436	− 0.02	0.566
264	Leptothrix sp._oral_taxon_025	P	–	− 3.50 (0.04)	−3.37 (0.08)	− 3.53 (0.06)	− 3.53 (0.06)	0.216	− 0.04	0.221
265	Pyramidobacter piscolens	P	–	− 3.53 (0.04)	− 3.44 (0.10)	− 3.60 (0.06)	−3.47 (0.07)	0.251	− 0.01	0.919
266	Sphingomonas sp._oral_taxon_006	P	–	− 3.55 (0.04)	−3.49 (0.08)	− 3.60 (0.05)	− 3.52 (0.06)	0.407	− 0.00	0.989
267	Porphyrobacter tepidarius	N	–	−3.58 (0.03)	−3.51 (0.08)	− 3.65 (0.05)	−3.54 (0.06)	0.216	0.01	0.869

Rank order, OTUs are sorted high to low, with most abundant OTU at the top of the table

The CLR OTU is interpreted as a log [2] fold-difference for the given species relative to the overall compositional geometric mean. A mean CLR of 3 indicates a 8-fold [23] higher abundance, and a mean CLR of − 3 indicates a 8-fold lower abundance, relative to the overall compositional geometric mean

*SE* standard error, *Pearson r* the Pearson product-moment correlation coefficient

*p*-values: **bolded** are significant at alpha 0.05; asterisk are significant at alpha 0.05 after Bonferroni correction

Culture status: N = named; U = unnamed; P = phylotyped

Socransky complex^32^: R = red; O = orange; P = purple; G = green; Y = yellow; B = blue; −-- = not part of the Socransky classification

Among all 1219 women (Table 4), Pearson correlations ranged from *r* = − 0.18 to *r* = 0.18. Eighty two (31%) taxa were significantly correlated with age (uncorrected *P* < 0.05; bolded), of which 28 (34.2%) remained significant after Bonferroni correction. The largest positive correlation was with *Oribacterium sp._oral_taxon 078* (*r* = 0.18; corrected *P* < 0.001); the most negative correlation was with *Strep. sanguinis* (*r* = − 0.18; corrected *P* < .001). Correlations between established pathogenic bacteria from Socransky’s complex [34] and age were of weak (*T. denticola, r* = − 0.04; *P. gingivalis, r* = 0.03; *F. nucleatum, r* = 0.03) to moderate (*Fusobacterium nucleatum polymorphum, r* = − 0.10; *T. forsythia, r* = 0.10) magnitude. Bacteria associated with healthy periodontium were correlated with age on a similar (*S. oralis*, *r* = − 0.10; *intermedius r* = − 0.10; *mutans, r* = 0.10) or somewhat stronger (*S. sanguinis, r* = − 0.16) magnitude.

**Fig. 5:**
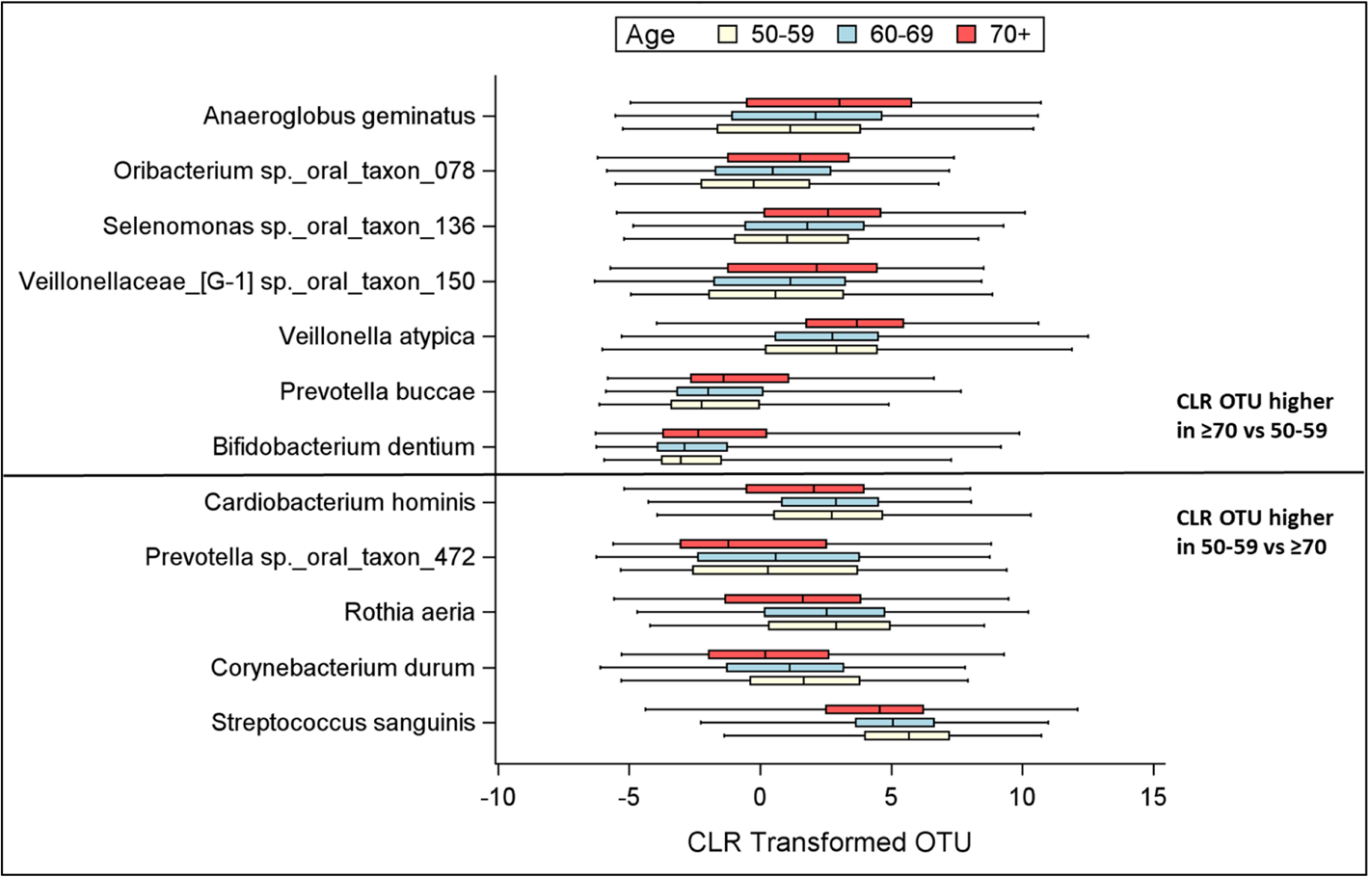
Box plots of mean CLR OTUs that differed between age categories (corrected *P* < 0.05). Box reflects the 25th, 50th, and 75th percentile CLR OTU; whiskers reflect the range of CLR OTU

Ninety (33.7%) bacteria were observed to be significantly different across age categories (uncorrected *P* < 0.05; bolded in Table 4), of which 12 (13.3%) remained significant after Bonferroni correction (corrected *P* < 0.05). Fig. 5 presents box-and-whisker plots depicting the variability of CLR OTUs for the 12 bacteria that were significantly different across age groups (corrected *P* < 0.001). Of these 12 bacteria, 7 were significantly higher in older than younger women; whilst the remaining 5 were higher in the younger women. *Bifidobacterium dentium* showed the greatest difference (0.73 CLR OTU units) between age groups among the bacteria observed to be higher in older women, whereas *S. sanguinis* showed the largest difference (1.19 CLR OTU units) between age groups for bacteria higher in younger women.

Tables 5 and 6 present additional measures used in previous studies to characterize the oral microbiome. Relative abundance for the overall cohort and according to age categories is shown in Table 5, ranked high to low, with dashed lines denoting the top 20 taxa. *V. dispar* demonstrated the highest relative abundance (mean, 8.9%), and the remaining bacterial order is quite similar to the top 20 when ordered according to CLR mean OTU (Table 2). Patterns of relative abundance across age categories also were generally comparable to those observed for CLR mean OTUs. Bacterial prevalence (present at any abundance) is shown in Table 6, for which a slightly different ordering is noted for the top 20 most prevalent bacteria compared to those ordered on CLR OTU or relative abundance. There were 12 (4.5%) bacteria prevalent at 99% or higher and 3 (1.1%) present in all samples (*S. oralis, V. dispar and parvula*). Differences in prevalence across age categories were modest. Among all women, prevalence of established pathogenic bacteria in Socransky’s complex was 86.8, 82.9 56.6, and 55.1% for *F. nucleatum, T. forsythia, T. denticola, and P. gingivalis*, respectively. Prevalence of *T. forsythia* did not vary consistently with age, whereas prevalence of *F. nucleatum and T. denticola tended to decline with age and P. gingivalis* tended to increase with age.

**Table 5:** Mean Relative Abundance for each of 267 bacterial species, overall and by age categories

Rank Order^a^	OTU label	Overall (*N* = 1219)	50–59 (*N* = 239)	60–69 (*N* = 554)	≥70 (*N* = 426)
		Relative Abundance (%) Mean (SE)	Relative Abundance (%) Mean (SE)	Relative Abundance (%) Mean (SE)	Relative Abundance (%) Mean (SE)
1	Veillonella dispar	8.94 (0.26)	8.66 (0.52)	8.52 (0.37)	9.64 (0.48)
2	Streptococcus oralis	7.68 (0.24)	9.52 (0.60)	7.95 (0.39)	6.29 (0.31)
3	Veillonella parvula	6.57 (0.22)	6.14 (0.44)	6.07 (0.30)	7.46 (0.42)
4	Fusobacterium nucleatum_subsp._vincentii	4.00 (0.14)	3.82 (0.32)	4.11 (0.21)	3.97 (0.23)
5	Selenomonas sputigena	2.94 (0.12)	2.23 (0.20)	2.66 (0.16)	3.70 (0.25)
6	Fusobacterium sp._oral_taxon_203	2.35 (0.13)	2.15 (0.28)	2.63 (0.21)	2.09 (0.20)
7	Prevotella oris	1.85 (0.07)	1.81 (0.14)	1.89 (0.11)	1.81 (0.10)
8	Prevotella nigrescens	1.78 (0.07)	1.64 (0.13)	1.81 (0.11)	1.81 (0.12)
9	Fusobacterium nucleatum_subsp._animalis	1.65 (0.06)	1.54 (0.13)	1.63 (0.09)	1.74 (0.09)
10	Selenomonas noxia	1.48 (0.07)	1.52 (0.18)	1.45 (0.11)	1.50 (0.11)
11	Fusobacterium nucleatum_subsp._polymorphum	1.42 (0.04)	1.57 (0.11)	1.50 (0.07)	1.24 (0.07)
12	Rothia dentocariosa	1.41 (0.10)	1.43 (0.17)	1.60 (0.18)	1.16 (0.13)
13	Streptococcus sanguinis	1.40 (0.07)	1.83 (0.14)	1.40 (0.09)	1.16 (0.13)
14	Alloprevotella tannerae	1.39 (0.07)	1.22 (0.16)	1.46 (0.12)	1.41 (0.11)
15	Haemophilus parainfluenzae	1.21 (0.09)	1.62 (0.24)	1.15 (0.13)	1.07 (0.14)
16	Corynebacterium matruchotii	1.17 (0.05)	1.34 (0.13)	1.24 (0.07)	0.99 (0.06)
17	Fretibacterium sp._oral_taxon_360	1.15 (0.07)	0.89 (0.13)	1.24 (0.11)	1.19 (0.10)
18	Streptococcus intermedius	1.05 (0.05)	1.27 (0.12)	1.13 (0.08)	0.84 (0.05)
19	TM7_[G-1] sp._oral_taxon_349	1.04 (0.06)	0.97 (0.14)	0.97 (0.07)	1.18 (0.14)
20	Streptococcus gordonii	1.03 (0.05)	0.98 (0.15)	1.01 (0.07)	1.09 (0.10)
21	TM7_[G-1] sp._oral_taxon_346	1.02 (0.05)	1.01 (0.11)	1.05 (0.07)	1.00 (0.08)
22	Porphyromonas gingivalis	0.94 (0.10)	0.95 (0.27)	0.74 (0.10)	1.21 (0.19)
23	Campylobacter gracilis	0.94 (0.02)	0.90 (0.06)	0.96 (0.04)	0.95 (0.04)
24	Fusobacterium naviforme	0.89 (0.06)	0.91 (0.13)	0.92 (0.10)	0.83 (0.07)
25	Parvimonas micra	0.88 (0.03)	0.87 (0.07)	0.96 (0.05)	0.78 (0.05)
26	Neisseria sicca	0.80 (0.07)	0.83 (0.21)	0.87 (0.11)	0.69 (0.09)
27	Leptotrichia wadei	0.79 (0.06)	0.81 (0.15)	0.74 (0.08)	0.86 (0.11)
28	Selenomonas artemidis	0.79 (0.05)	0.87 (0.12)	0.75 (0.07)	0.79 (0.08)
29	Bacteroidales_[G-2] sp._oral_taxon_274	0.79 (0.04)	0.64 (0.08)	0.85 (0.07)	0.79 (0.06)
30	Prevotella sp._oral_taxon_317	0.77 (0.04)	0.67 (0.07)	0.80 (0.05)	0.78 (0.06)
31	Anaeroglobus geminatus	0.67 (0.04)	0.43 (0.08)	0.58 (0.06)	0.92 (0.09)
32	Capnocytophaga granulosa	0.65 (0.03)	0.63 (0.09)	0.66 (0.05)	0.64 (0.06)
33	Streptococcus salivarius	0.64 (0.05)	0.61 (0.09)	0.67 (0.09)	0.64 (0.07)
34	TM7_[G-1] sp._oral_taxon_952	0.64 (0.03)	0.72 (0.08)	0.71 (0.05)	0.50 (0.05)
35	Prevotella denticola	0.62 (0.05)	0.49 (0.07)	0.57 (0.08)	0.76 (0.09)
36	Capnocytophaga leadbetteri	0.61 (0.03)	0.59 (0.07)	0.63 (0.05)	0.61 (0.07)
37	Gemella morbillorum	0.61 (0.02)	0.69 (0.07)	0.65 (0.04)	0.52 (0.04)
38	Veillonella atypica	0.61 (0.05)	0.50 (0.09)	0.55 (0.07)	0.75 (0.08)
39	Streptococcus mutans	0.60 (0.05)	0.47 (0.09)	0.51 (0.07)	0.80 (0.10)
40	Fretibacterium sp._oral_taxon_359	0.60 (0.06)	0.77 (0.17)	0.60 (0.09)	0.49 (0.07)
41	Prevotella intermedia	0.59 (0.06)	0.62 (0.15)	0.63 (0.09)	0.52 (0.09)
42	Dialister invisus	0.57 (0.02)	0.47 (0.04)	0.60 (0.04)	0.60 (0.04)
43	Granulicatella adiacens	0.56 (0.02)	0.65 (0.05)	0.55 (0.03)	0.53 (0.03)
44	Porphyromonas endodontalis	0.55 (0.04)	0.63 (0.08)	0.57 (0.05)	0.49 (0.06)
45	Streptococcus cristatus	0.55 (0.03)	0.51 (0.07)	0.59 (0.05)	0.53 (0.04)
46	Capnocytophaga gingivalis	0.55 (0.03)	0.60 (0.07)	0.55 (0.04)	0.51 (0.04)
47	Prevotella pleuritidis	0.53 (0.04)	0.51 (0.08)	0.56 (0.06)	0.51 (0.06)
48	Neisseria elongata	0.53 (0.04)	0.66 (0.10)	0.55 (0.05)	0.43 (0.07)
49	Actinomyces naeslundii	0.52 (0.02)	0.62 (0.05)	0.53 (0.03)	0.46 (0.03)
50	Selenomonas sp._oral_taxon_137	0.51 (0.04)	0.51 (0.09)	0.52 (0.05)	0.50 (0.07)
51	Tannerella forsythia	0.49 (0.03)	0.42 (0.06)	0.46 (0.04)	0.58 (0.05)
52	TM7_[G-5] sp._oral_taxon_356	0.47 (0.03)	0.36 (0.07)	0.51 (0.06)	0.48 (0.06)
53	Campylobacter showae	0.46 (0.02)	0.44 (0.04)	0.47 (0.04)	0.46 (0.03)
54	Fretibacterium fastidiosum	0.46 (0.03)	0.54 (0.10)	0.45 (0.04)	0.42 (0.04)
55	Rothia aeria	0.45 (0.03)	0.45 (0.05)	0.55 (0.06)	0.33 (0.04)
56	Capnocytophaga sputigena	0.45 (0.03)	0.47 (0.05)	0.44 (0.04)	0.46 (0.05)
57	Gemella haemolysans	0.44 (0.04)	0.54 (0.08)	0.45 (0.07)	0.38 (0.06)
58	Leptotrichia hongkongensis	0.43 (0.03)	0.36 (0.04)	0.44 (0.05)	0.44 (0.05)
59	Neisseria flavescens	0.42 (0.04)	0.29 (0.06)	0.38 (0.06)	0.55 (0.09)
60	Neisseria oralis	0.42 (0.05)	0.59 (0.15)	0.32 (0.04)	0.45 (0.11)
61	Streptococcus anginosus	0.42 (0.03)	0.38 (0.05)	0.38 (0.04)	0.49 (0.06)
62	Aggregatibacter aphrophilus	0.37 (0.04)	0.33 (0.05)	0.45 (0.07)	0.28 (0.05)
63	Kingella oralis	0.36 (0.02)	0.41 (0.05)	0.31 (0.02)	0.39 (0.04)
64	Leptotrichia shahii	0.36 (0.04)	0.32 (0.07)	0.26 (0.03)	0.50 (0.08)
65	Leptotrichia buccalis	0.35 (0.03)	0.36 (0.07)	0.40 (0.05)	0.29 (0.05)
66	Selenomonas sp._oral_taxon_134	0.34 (0.02)	0.23 (0.04)	0.32 (0.03)	0.43 (0.05)
67	Leptotrichia hofstadii	0.33 (0.04)	0.42 (0.11)	0.34 (0.05)	0.27 (0.05)
68	Leptotrichia sp._oral_taxon_417	0.32 (0.03)	0.23 (0.04)	0.36 (0.05)	0.32 (0.05)
69	Cardiobacterium hominis	0.32 (0.02)	0.47 (0.08)	0.33 (0.02)	0.23 (0.02)
70	Selenomonas sp._oral_taxon_136	0.31 (0.02)	0.20 (0.03)	0.29 (0.03)	0.40 (0.04)
71	Eikenella corrodens	0.31 (0.01)	0.34 (0.03)	0.33 (0.02)	0.25 (0.02)
72	Prevotella melaninogenica	0.30 (0.02)	0.25 (0.03)	0.28 (0.03)	0.36 (0.04)
73	Porphyromonas sp._oral_taxon_279	0.30 (0.02)	0.28 (0.04)	0.32 (0.04)	0.30 (0.03)
74	Treponema denticola	0.28 (0.02)	0.26 (0.05)	0.28 (0.03)	0.31 (0.04)
75	Filifactor alocis	0.28 (0.02)	0.29 (0.05)	0.29 (0.04)	0.27 (0.04)
76	Actinomyces oris	0.28 (0.02)	0.31 (0.04)	0.27 (0.02)	0.27 (0.03)
77	Parvimonas sp._oral_taxon_393	0.28 (0.02)	0.36 (0.05)	0.28 (0.02)	0.23 (0.02)
78	Veillonellaceae_[G-1] sp._oral_taxon_150	0.27 (0.02)	0.22 (0.04)	0.23 (0.02)	0.35 (0.03)
79	Actinomyces sp._oral_taxon_169	0.27 (0.02)	0.39 (0.06)	0.26 (0.02)	0.22 (0.03)
80	Leptotrichia sp._oral_taxon_212	0.27 (0.02)	0.28 (0.03)	0.28 (0.02)	0.24 (0.04)
81	Rothia mucilaginosa	0.27 (0.02)	0.21 (0.03)	0.28 (0.04)	0.29 (0.03)
82	Selenomonas infelix	0.27 (0.02)	0.21 (0.02)	0.27 (0.03)	0.29 (0.03)
83	Cardiobacterium valvarum	0.25 (0.01)	0.31 (0.04)	0.26 (0.02)	0.21 (0.02)
84	Capnocytophaga sp._oral_taxon_326	0.25 (0.02)	0.26 (0.04)	0.28 (0.03)	0.22 (0.03)
85	Prevotella sp._oral_taxon_472	0.25 (0.02)	0.30 (0.05)	0.27 (0.02)	0.19 (0.02)
86	Treponema socranskii	0.25 (0.01)	0.22 (0.02)	0.24 (0.01)	0.27 (0.02)
87	Streptococcus constellatus	0.25 (0.02)	0.19 (0.03)	0.25 (0.03)	0.28 (0.03)
88	Veillonella rogosae	0.24 (0.02)	0.21 (0.04)	0.27 (0.04)	0.23 (0.03)
89	Fusobacterium nucleatum_subsp._nucleatum	0.24 (0.03)	0.21 (0.06)	0.19 (0.04)	0.34 (0.07)
90	Prevotella sp._oral_taxon_300	0.23 (0.01)	0.24 (0.03)	0.20 (0.01)	0.27 (0.03)
91	Veillonellaceae_[G-1] sp._oral_taxon_155	0.23 (0.02)	0.20 (0.03)	0.20 (0.02)	0.28 (0.03)
92	Campylobacter concisus	0.22 (0.01)	0.20 (0.02)	0.23 (0.01)	0.23 (0.02)
93	Leptotrichia sp._oral_taxon_225	0.22 (0.02)	0.26 (0.06)	0.28 (0.04)	0.12 (0.03)
94	Selenomonas sp._oral_taxon_892	0.22 (0.01)	0.24 (0.04)	0.21 (0.02)	0.21 (0.02)
95	Catonella morbi	0.21 (0.01)	0.23 (0.02)	0.21 (0.01)	0.19 (0.01)
96	Aggregatibacter segnis	0.20 (0.02)	0.21 (0.05)	0.24 (0.04)	0.15 (0.02)
97	Prevotella oulorum	0.20 (0.01)	0.21 (0.03)	0.18 (0.01)	0.22 (0.02)
98	Leptotrichia sp._oral_taxon_392	0.19 (0.01)	0.20 (0.04)	0.22 (0.02)	0.16 (0.02)
99	Dialister pneumosintes	0.19 (0.01)	0.18 (0.03)	0.19 (0.02)	0.20 (0.02)
100	Veillonella denticariosi	0.19 (0.03)	0.17 (0.06)	0.17 (0.04)	0.22 (0.07)
101	Peptostreptococcaceae_[XI][G-9] [Eubacterium]_brac	0.19 (0.01)	0.21 (0.02)	0.19 (0.01)	0.17 (0.01)
102	Megasphaera micronuciformis	0.19 (0.02)	0.15 (0.03)	0.17 (0.02)	0.23 (0.04)
103	Lautropia mirabilis	0.18 (0.01)	0.23 (0.03)	0.20 (0.02)	0.14 (0.01)
104	Streptococcus parasanguinis_II	0.18 (0.02)	0.15 (0.03)	0.17 (0.02)	0.21 (0.03)
105	Porphyromonas sp._oral_taxon_284	0.18 (0.01)	0.18 (0.02)	0.19 (0.02)	0.17 (0.02)
106	Selenomonas sp._oral_taxon_919	0.18 (0.01)	0.18 (0.02)	0.17 (0.02)	0.20 (0.03)
107	Bergeyella sp._oral_taxon_322	0.18 (0.01)	0.22 (0.02)	0.19 (0.01)	0.14 (0.01)
108	Leptotrichia sp._oral_taxon_498	0.18 (0.02)	0.14 (0.03)	0.16 (0.03)	0.22 (0.04)
109	Kingella denitrificans	0.17 (0.01)	0.17 (0.04)	0.17 (0.02)	0.18 (0.02)
110	Prevotella maculosa	0.17 (0.01)	0.16 (0.02)	0.16 (0.01)	0.18 (0.01)
111	Atopobium rimae	0.17 (0.01)	0.12 (0.02)	0.15 (0.02)	0.21 (0.03)
112	Corynebacterium durum	0.17 (0.01)	0.24 (0.03)	0.16 (0.01)	0.13 (0.03)
113	TM7_[G-1] sp._oral_taxon_488	0.17 (0.02)	0.23 (0.07)	0.18 (0.02)	0.11 (0.01)
114	Capnocytophaga sp._oral_taxon_336	0.17 (0.01)	0.15 (0.02)	0.17 (0.01)	0.17 (0.02)
115	Selenomonas flueggei	0.16 (0.01)	0.15 (0.03)	0.16 (0.02)	0.18 (0.03)
116	Aggregatibacter sp._oral_taxon_458	0.16 (0.02)	0.14 (0.02)	0.16 (0.02)	0.17 (0.03)
117	Fretibacterium sp._oral_taxon_362	0.16 (0.02)	0.15 (0.04)	0.18 (0.04)	0.14 (0.03)
118	Neisseria subflava	0.16 (0.02)	0.14 (0.04)	0.16 (0.04)	0.17 (0.03)
119	Peptostreptococcaceae_[XI][G-7] [Eubacterium]_yuri	0.16 (0.01)	0.18 (0.02)	0.17 (0.01)	0.12 (0.01)
120	TM7_[G-1] sp._oral_taxon_869	0.16 (0.03)	0.15 (0.04)	0.20 (0.06)	0.11 (0.02)
121	Lachnoanaerobaculum saburreum	0.15 (0.01)	0.14 (0.02)	0.15 (0.01)	0.15 (0.01)
122	Prevotella oralis	0.15 (0.01)	0.09 (0.01)	0.13 (0.01)	0.20 (0.02)
123	TM7_[G-2] sp._oral_taxon_350	0.15 (0.02)	0.12 (0.03)	0.14 (0.02)	0.17 (0.04)
124	Megasphaera sp._oral_taxon_123	0.14 (0.03)	0.17 (0.05)	0.12 (0.03)	0.15 (0.06)
125	Aggregatibacter paraphrophilus	0.14 (0.02)	0.18 (0.06)	0.18 (0.03)	0.07 (0.02)
126	Desulfobulbus sp._oral_taxon_041	0.14 (0.01)	0.11 (0.02)	0.13 (0.02)	0.17 (0.02)
127	Actinomyces massiliensis	0.14 (0.01)	0.18 (0.02)	0.15 (0.02)	0.10 (0.01)
128	Johnsonella ignava	0.13 (0.01)	0.13 (0.02)	0.14 (0.01)	0.13 (0.01)
129	Prevotella loescheii	0.13 (0.01)	0.15 (0.03)	0.15 (0.02)	0.09 (0.02)
130	Lachnospiraceae_[G-3] sp._oral_taxon_100	0.13 (0.01)	0.12 (0.01)	0.14 (0.01)	0.12 (0.01)
131	Actinomyces johnsonii	0.13 (0.01)	0.12 (0.01)	0.13 (0.02)	0.12 (0.02)
132	TM7_[G-1] sp._oral_taxon_347	0.12 (0.02)	0.16 (0.05)	0.14 (0.02)	0.08 (0.02)
133	Bacteroidetes_[G-5] sp._oral_taxon_511	0.12 (0.01)	0.10 (0.02)	0.13 (0.02)	0.12 (0.02)
134	Actinomyces sp._oral_taxon_180	0.12 (0.01)	0.13 (0.01)	0.12 (0.01)	0.10 (0.01)
135	Prevotella salivae	0.12 (0.01)	0.09 (0.02)	0.12 (0.01)	0.13 (0.02)
136	Actinomyces sp._oral_taxon_171	0.12 (0.01)	0.12 (0.02)	0.12 (0.01)	0.11 (0.01)
137	Selenomonas sp._oral_taxon_146	0.12 (0.01)	0.12 (0.02)	0.11 (0.01)	0.12 (0.01)
138	Abiotrophia defectiva	0.12 (0.01)	0.14 (0.03)	0.13 (0.01)	0.09 (0.01)
139	Pseudomonas fluorescens	0.11 (0.05)	0.07 (0.04)	0.15 (0.10)	0.10 (0.04)
140	Veillonella sp._oral_taxon_780	0.11 (0.02)	0.16 (0.06)	0.12 (0.04)	0.08 (0.03)
141	Porphyromonas catoniae	0.11 (0.01)	0.13 (0.03)	0.13 (0.02)	0.08 (0.01)
142	Mitsuokella sp._oral_taxon_131	0.11 (0.02)	0.05 (0.01)	0.11 (0.03)	0.14 (0.04)
143	Actinobaculum sp._oral_taxon_183	0.11 (0.01)	0.12 (0.02)	0.11 (0.01)	0.09 (0.01)
144	Oribacterium sp._oral_taxon_078	0.11 (0.01)	0.07 (0.01)	0.10 (0.01)	0.14 (0.01)
145	Treponema sp._oral_taxon_237	0.11 (0.01)	0.11 (0.03)	0.10 (0.02)	0.11 (0.02)
146	Haemophilus sp._oral_taxon_036	0.10 (0.01)	0.11 (0.03)	0.10 (0.02)	0.09 (0.02)
147	Leptotrichia sp._oral_taxon_215	0.10 (0.01)	0.12 (0.02)	0.10 (0.01)	0.08 (0.01)
148	Prevotella histicola	0.10 (0.01)	0.06 (0.01)	0.08 (0.02)	0.15 (0.03)
149	TM7_[G-1] sp._oral_taxon_348	0.10 (0.01)	0.08 (0.01)	0.12 (0.01)	0.09 (0.01)
150	Peptostreptococcus stomatis	0.10 (0.01)	0.11 (0.02)	0.10 (0.01)	0.08 (0.01)
151	Prevotella veroralis	0.10 (0.01)	0.08 (0.02)	0.12 (0.02)	0.08 (0.02)
152	Streptococcus sp._oral_taxon_056	0.10 (0.01)	0.11 (0.02)	0.09 (0.01)	0.09 (0.01)
153	Selenomonas sp._oral_taxon_126	0.10 (0.01)	0.09 (0.02)	0.10 (0.01)	0.09 (0.01)
154	Treponema sp._oral_taxon_231	0.09 (0.01)	0.07 (0.01)	0.10 (0.01)	0.09 (0.01)
155	Neisseria bacilliformis	0.09 (0.01)	0.11 (0.04)	0.08 (0.01)	0.08 (0.02)
156	Actinomyces gerencseriae	0.09 (0.01)	0.09 (0.01)	0.08 (0.01)	0.09 (0.01)
157	Prevotella dentalis	0.08 (0.01)	0.08 (0.02)	0.08 (0.02)	0.08 (0.01)
158	Fusobacterium periodonticum	0.08 (0.01)	0.06 (0.01)	0.09 (0.01)	0.09 (0.01)
159	Prevotella pallens	0.08 (0.01)	0.08 (0.02)	0.06 (0.01)	0.11 (0.02)
160	Centipeda periodontii	0.08 (0.01)	0.05 (0.01)	0.10 (0.01)	0.08 (0.01)
161	Scardovia wiggsiae	0.08 (0.01)	0.09 (0.03)	0.07 (0.02)	0.09 (0.02)
162	Streptococcus sp._oral_taxon_074	0.08 (0.01)	0.07 (0.01)	0.09 (0.01)	0.07 (0.01)
163	Haemophilus parahaemolyticus	0.08 (0.02)	0.09 (0.03)	0.11 (0.03)	0.04 (0.02)
164	Tannerella sp._oral_taxon_286	0.08 (0.00)	0.06 (0.01)	0.08 (0.01)	0.08 (0.01)
165	Selenomonas sp._oral_taxon_936	0.08 (0.01)	0.06 (0.01)	0.07 (0.01)	0.10 (0.02)
166	Actinomyces sp._oral_taxon_170	0.08 (0.01)	0.06 (0.01)	0.10 (0.02)	0.06 (0.02)
167	Ruminococcaceae_[G-1] sp._oral_taxon_075	0.08 (0.01)	0.09 (0.02)	0.08 (0.01)	0.07 (0.01)
168	Bifidobacterium dentium	0.08 (0.01)	0.07 (0.02)	0.06 (0.01)	0.10 (0.02)
169	Alloprevotella rava	0.08 (0.01)	0.07 (0.01)	0.07 (0.01)	0.09 (0.01)
170	Atopobium parvulum	0.08 (0.01)	0.06 (0.01)	0.06 (0.01)	0.11 (0.02)
171	Ottowia sp._oral_taxon_894	0.08 (0.01)	0.08 (0.02)	0.09 (0.01)	0.06 (0.01)
172	Prevotella sp._oral_taxon_313	0.07 (0.01)	0.09 (0.02)	0.05 (0.01)	0.10 (0.03)
173	TM7_[G-6] sp._oral_taxon_870	0.07 (0.01)	0.08 (0.02)	0.08 (0.01)	0.06 (0.01)
174	Capnocytophaga sp._oral_taxon_864	0.07 (0.01)	0.08 (0.01)	0.08 (0.01)	0.05 (0.01)
175	Actinomyces meyeri	0.07 (0.00)	0.08 (0.01)	0.08 (0.01)	0.06 (0.00)
176	Leptotrichia sp._oral_taxon_223	0.07 (0.01)	0.05 (0.01)	0.07 (0.02)	0.09 (0.02)
177	Capnocytophaga sp._oral_taxon_338	0.07 (0.00)	0.07 (0.01)	0.07 (0.01)	0.07 (0.01)
178	Streptococcus parasanguinis_I	0.07 (0.01)	0.06 (0.01)	0.06 (0.01)	0.08 (0.01)
179	Pseudoramibacter alactolyticus	0.07 (0.01)	0.10 (0.05)	0.05 (0.01)	0.07 (0.01)
180	Haemophilus haemolyticus	0.07 (0.01)	0.15 (0.05)	0.05 (0.01)	0.05 (0.01)
181	Neisseria pharyngis	0.07 (0.02)	0.06 (0.03)	0.04 (0.02)	0.10 (0.05)
182	Treponema maltophilum	0.07 (0.00)	0.06 (0.01)	0.06 (0.00)	0.08 (0.01)
183	Bradyrhizobium elkanii	0.07 (0.02)	0.12 (0.06)	0.05 (0.01)	0.05 (0.02)
184	Alloprevotella sp._oral_taxon_473	0.07 (0.01)	0.05 (0.01)	0.08 (0.02)	0.06 (0.01)
185	Streptococcus lactarius	0.06 (0.01)	0.11 (0.04)	0.05 (0.01)	0.06 (0.01)
186	Prevotella sp._oral_taxon_292	0.06 (0.00)	0.05 (0.01)	0.06 (0.01)	0.08 (0.01)
187	Capnocytophaga sp._oral_taxon_332	0.06 (0.01)	0.08 (0.04)	0.07 (0.01)	0.05 (0.01)
188	Prevotella saccharolytica	0.06 (0.00)	0.06 (0.01)	0.07 (0.01)	0.06 (0.00)
189	Leptotrichia sp._oral_taxon_879	0.06 (0.02)	0.14 (0.11)	0.03 (0.01)	0.05 (0.01)
190	Prevotella sp._oral_taxon_314	0.06 (0.01)	0.05 (0.02)	0.05 (0.01)	0.07 (0.01)
191	Actinobaculum sp._oral_taxon_848	0.06 (0.00)	0.04 (0.01)	0.06 (0.01)	0.06 (0.01)
192	Fretibacterium sp._oral_taxon_358	0.06 (0.01)	0.04 (0.02)	0.06 (0.02)	0.06 (0.01)
193	Streptococcus sobrinus	0.06 (0.01)	0.00 (0.00)	0.03 (0.01)	0.12 (0.04)
194	Aggregatibacter actinomycetemcomitans	0.06 (0.01)	0.06 (0.02)	0.06 (0.02)	0.05 (0.02)
195	Treponema lecithinolyticum	0.06 (0.01)	0.06 (0.01)	0.05 (0.01)	0.06 (0.01)
196	Capnocytophaga sp._oral_taxon_902	0.06 (0.01)	0.08 (0.02)	0.05 (0.01)	0.05 (0.01)
197	Peptostreptococcaceae_[XI][G-5] [Eubacterium]_saph	0.06 (0.01)	0.04 (0.01)	0.07 (0.01)	0.05 (0.01)
198	Lachnoanaerobaculum umeaense	0.06 (0.00)	0.06 (0.01)	0.06 (0.01)	0.05 (0.01)
199	Peptostreptococcaceae_[XI][G-6] [Eubacterium]_noda	0.05 (0.00)	0.06 (0.01)	0.05 (0.01)	0.06 (0.01)
200	Veillonellaceae_[G-1] sp._oral_taxon_145	0.05 (0.01)	0.04 (0.01)	0.05 (0.01)	0.06 (0.01)
201	TM7_[G-1] sp._oral_taxon_352	0.05 (0.01)	0.05 (0.01)	0.05 (0.01)	0.06 (0.01)
202	Capnocytophaga sp._oral_taxon_412	0.05 (0.01)	0.05 (0.01)	0.05 (0.01)	0.06 (0.01)
203	Gemella sanguinis	0.05 (0.00)	0.04 (0.01)	0.05 (0.01)	0.06 (0.01)
204	Aggregatibacter sp._oral_taxon_513	0.05 (0.01)	0.03 (0.02)	0.07 (0.01)	0.04 (0.01)
205	Selenomonas sp._oral_taxon_133	0.05 (0.01)	0.06 (0.02)	0.05 (0.01)	0.05 (0.01)
206	Granulicatella elegans	0.05 (0.01)	0.06 (0.01)	0.05 (0.01)	0.05 (0.01)
207	Veillonellaceae_[G-1] sp._oral_taxon_129	0.05 (0.01)	0.03 (0.01)	0.04 (0.01)	0.07 (0.02)
208	Selenomonas dianae	0.05 (0.01)	0.04 (0.02)	0.04 (0.01)	0.07 (0.03)
209	Prevotella sp._oral_taxon_526	0.05 (0.01)	0.02 (0.01)	0.06 (0.02)	0.05 (0.01)
210	Porphyromonas sp._oral_taxon_275	0.05 (0.01)	0.04 (0.01)	0.06 (0.01)	0.03 (0.01)
211	Anaerolineae_[G-1] sp._oral_taxon_439	0.05 (0.01)	0.04 (0.01)	0.04 (0.01)	0.06 (0.01)
212	Stomatobaculum longum	0.05 (0.00)	0.04 (0.01)	0.04 (0.00)	0.05 (0.01)
213	Olsenella sp._oral_taxon_807	0.04 (0.00)	0.04 (0.01)	0.04 (0.00)	0.05 (0.00)
214	Capnocytophaga sp._oral_taxon_323	0.04 (0.00)	0.03 (0.01)	0.05 (0.01)	0.05 (0.01)
215	Capnocytophaga sp._oral_taxon_903	0.04 (0.00)	0.03 (0.01)	0.04 (0.01)	0.05 (0.01)
216	Fretibacterium sp._oral_taxon_361	0.04 (0.01)	0.01 (0.00)	0.05 (0.02)	0.05 (0.02)
217	Leptotrichia sp._oral_taxon_219	0.04 (0.00)	0.05 (0.02)	0.04 (0.00)	0.04 (0.01)
218	Prevotella sp._oral_taxon_306	0.04 (0.01)	0.04 (0.02)	0.03 (0.01)	0.06 (0.01)
219	Atopobium sp._oral_taxon_199	0.04 (0.01)	0.04 (0.01)	0.04 (0.01)	0.03 (0.01)
220	Capnocytophaga sp._oral_taxon_324	0.04 (0.01)	0.03 (0.01)	0.04 (0.01)	0.05 (0.01)
221	Prevotella buccae	0.04 (0.00)	0.02 (0.00)	0.04 (0.01)	0.05 (0.01)
222	Prevotella baroniae	0.04 (0.00)	0.02 (0.01)	0.04 (0.01)	0.04 (0.01)
223	Lachnospiraceae_[G-8] sp._oral_taxon_500	0.04 (0.00)	0.04 (0.01)	0.04 (0.01)	0.04 (0.00)
224	Prevotella sp._oral_taxon_376	0.04 (0.00)	0.05 (0.01)	0.04 (0.01)	0.03 (0.01)
225	Porphyromonas sp._oral_taxon_278	0.04 (0.01)	0.03 (0.01)	0.04 (0.01)	0.04 (0.01)
226	Solobacterium moorei	0.04 (0.00)	0.04 (0.00)	0.04 (0.00)	0.04 (0.00)
227	Campylobacter curvus	0.03 (0.00)	0.05 (0.01)	0.03 (0.01)	0.03 (0.01)
228	Prevotella micans	0.03 (0.00)	0.03 (0.01)	0.03 (0.01)	0.04 (0.01)
229	Bacteroidaceae_[G-1] sp._oral_taxon_272	0.03 (0.00)	0.03 (0.01)	0.03 (0.00)	0.04 (0.01)
230	Streptococcus australis	0.03 (0.00)	0.03 (0.01)	0.03 (0.00)	0.04 (0.01)
231	Tannerella sp._oral_taxon_808	0.03 (0.00)	0.02 (0.00)	0.03 (0.00)	0.04 (0.01)
232	Bergeyella sp._oral_taxon_907	0.03 (0.00)	0.03 (0.01)	0.04 (0.01)	0.03 (0.00)
233	Prevotella multiformis	0.03 (0.01)	0.01 (0.00)	0.03 (0.01)	0.05 (0.02)
234	Fusobacterium sp._oral_taxon_370	0.03 (0.01)	0.02 (0.01)	0.04 (0.01)	0.03 (0.01)
235	Capnocytophaga sp._oral_taxon_380	0.03 (0.01)	0.03 (0.01)	0.04 (0.01)	0.03 (0.01)
236	Peptostreptococcaceae_[XI][G-1] [Eubacterium]_infi	0.03 (0.00)	0.03 (0.01)	0.03 (0.00)	0.04 (0.00)
237	Treponema medium	0.03 (0.00)	0.03 (0.01)	0.04 (0.01)	0.02 (0.01)
238	Lachnoanaerobaculum orale	0.03 (0.00)	0.04 (0.01)	0.02 (0.00)	0.03 (0.01)
239	Alloprevotella sp._oral_taxon_308	0.03 (0.00)	0.03 (0.00)	0.03 (0.00)	0.03 (0.01)
240	SR1_[G-1] sp._oral_taxon_874	0.03 (0.00)	0.03 (0.00)	0.03 (0.01)	0.02 (0.00)
241	Actinomyces israelii	0.03 (0.00)	0.03 (0.00)	0.03 (0.00)	0.03 (0.00)
242	Shuttleworthia satelles	0.03 (0.00)	0.02 (0.01)	0.03 (0.01)	0.04 (0.01)
243	Mycoplasma salivarium	0.03 (0.00)	0.02 (0.00)	0.03 (0.00)	0.03 (0.00)
244	Leptotrichia goodfellowii	0.03 (0.00)	0.04 (0.01)	0.03 (0.01)	0.02 (0.00)
245	Streptococcus sinensis	0.03 (0.00)	0.03 (0.01)	0.02 (0.00)	0.04 (0.01)
246	TM7_[G-3] sp._oral_taxon_351	0.03 (0.00)	0.02 (0.00)	0.03 (0.00)	0.03 (0.00)
247	Mitsuokella sp._oral_taxon_521	0.03 (0.01)	0.01 (0.01)	0.02 (0.01)	0.04 (0.02)
248	Selenomonas sp._oral_taxon_442	0.03 (0.01)	0.01 (0.00)	0.04 (0.01)	0.02 (0.01)
249	Actinomyces sp._oral_taxon_178	0.03 (0.00)	0.02 (0.00)	0.03 (0.00)	0.03 (0.00)
250	Johnsonella sp._oral_taxon_166	0.02 (0.00)	0.02 (0.01)	0.03 (0.00)	0.02 (0.00)
251	Lactobacillus gasseri	0.02 (0.01)	0.01 (0.00)	0.02 (0.01)	0.03 (0.01)
252	Treponema sp._oral_taxon_247	0.02 (0.01)	0.01 (0.01)	0.03 (0.01)	0.02 (0.01)
253	GN02_[G-2] sp._oral_taxon_873	0.02 (0.00)	0.03 (0.02)	0.02 (0.01)	0.02 (0.01)
254	Selenomonas sp._oral_taxon_937	0.02 (0.00)	0.01 (0.00)	0.02 (0.01)	0.02 (0.00)
255	Atopobium sp._oral_taxon_416	0.02 (0.01)	0.00 (0.00)	0.01 (0.01)	0.04 (0.02)
256	Sphingomonas echinoides	0.02 (0.00)	0.02 (0.01)	0.02 (0.00)	0.02 (0.01)
257	Butyrivibrio sp._oral_taxon_080	0.02 (0.00)	0.02 (0.01)	0.02 (0.01)	0.02 (0.00)
258	Treponema vincentii	0.02 (0.00)	0.02 (0.00)	0.03 (0.00)	0.01 (0.00)
259	Selenomonas sp._oral_taxon_149	0.02 (0.00)	0.01 (0.00)	0.02 (0.01)	0.02 (0.00)
260	Selenomonas sp._oral_taxon_478	0.02 (0.00)	0.01 (0.00)	0.01 (0.00)	0.02 (0.01)
261	Prevotella sp._oral_taxon_475	0.02 (0.00)	0.02 (0.01)	0.02 (0.00)	0.02 (0.01)
262	Pyramidobacter piscolens	0.01 (0.00)	0.01 (0.01)	0.00 (0.00)	0.01 (0.00)
263	Microbacterium flavescens	0.00 (0.00)	0.00 (0.00)	0.00 (0.00)	0.00 (0.00)
264	Brevundimonas diminuta	0.00 (0.00)	0.00 (0.00)	0.00 (0.00)	0.00 (0.00)
265	Sphingomonas sp._oral_taxon_006	0.00 (0.00)	0.00 (0.00)	0.00 (0.00)	0.00 (0.00)
266	Leptothrix sp._oral_taxon_025	0.00 (0.00)	0.00 (0.00)	0.00 (0.00)	0.00 (0.00)
267	Porphyrobacter tepidarius	0.00 (0.00)	0.00 (0.00)	0.00 (0.00)	0.00 (0.00)

^a^Bacteria in the table are rank ordered according to their mean relative abundance (%) in the overall cohort

Dashed line inserted below the top 20 taxa

**Table 6:** Prevalence (present, absent) for each of 267 bacterial species identified, overall and by age categories

Rank Order^a^	OTU Label		Age categories (years)		
		Overall (*N* = 1219)	50–59 (*N* = 239)	60–69 (*N* = 554)	≥70 (*N* = 426)
		%	%	%	%
1	Streptococcus oralis	100	100	100	100
1	Veillonella dispar	100	100	100	100
1	Veillonella parvula	100	100	100	100
4	Selenomonas sputigena	99.8	99.6	99.8	99.8
5	Fusobacterium nucleatum_subsp._vincentii	99.6	99.6	99.6	99.5
6	Fusobacterium nucleatum_subsp._animalis	99.3	99.6	99.3	99.3
6	Granulicatella adiacens	99.3	99.2	99.5	99.3
8	Streptococcus sanguinis	99.3	100	99.3	99.1
9	Rothia dentocariosa	99.2	99.2	99.3	99.1
10	Campylobacter gracilis	99.1	99.6	98.9	99.1
11	Selenomonas noxia	99.0	98.7	99.1	99.1
11	Streptococcus gordonii	99.0	98.7	98.9	99.3
13	Fusobacterium nucleatum_subsp._polymorphum	98.6	99.2	98.9	97.9
14	Fusobacterium sp._oral_taxon_203	98.4	98.7	98.0	98.8
14	Streptococcus cristatus	98.4	98.7	98.2	98.6
14	Fusobacterium naviforme	98.4	99.2	98.6	97.7
17	Actinomyces naeslundii	98.0	97.9	98.2	97.9
18	Corynebacterium matruchotii	97.5	96.7	97.3	98.1
19	Prevotella oris	97.2	97.5	97.3	96.9
20	Actinomyces oris	97.1	97.5	96.9	97.2
21	Streptococcus salivarius	97.0	96.7	96.8	97.7
21	Parvimonas micra	97.0	95.8	96.9	97.7
23	Haemophilus parainfluenzae	96.9	98.7	97.3	95.3
24	Streptococcus intermedius	96.8	97.9	95.5	97.9
25	Veillonella atypica	96.4	95.0	95.7	98.1
26	Rothia mucilaginosa	96.1	96.2	96.2	96.0
27	Dialister invisus	95.4	95.8	94.6	96.2
28	Campylobacter concisus	95.2	95.8	94.8	95.3
29	Capnocytophaga gingivalis	94.9	95.8	94.9	94.4
29	TM7_[G-1] sp._oral_taxon_346	94.9	95.0	96.0	93.4
31	Prevotella nigrescens	94.2	95.0	94.2	93.7
32	Kingella oralis	93.0	92.9	94.2	91.5
33	Bergeyella sp._oral_taxon_322	92.7	92.9	91.9	93.7
34	Gemella haemolysans	92.5	91.6	94.2	90.8
35	Actinomyces sp._oral_taxon_169	92.3	93.7	93.0	90.6
36	Actinomyces sp._oral_taxon_180	92.1	94.1	92.2	90.8
36	Eikenella corrodens	92.1	95.4	92.6	89.7
38	Prevotella melaninogenica	91.6	92.1	90.6	92.7
38	TM7_[G-1] sp._oral_taxon_349	91.6	92.9	92.4	89.7
40	Campylobacter showae	91.3	91.6	91.0	91.5
41	Actinomyces johnsonii	91.1	91.6	91.7	90.1
42	Capnocytophaga granulosa	90.8	89.5	92.4	89.4
43	Selenomonas sp._oral_taxon_136	90.7	89.1	90.4	92.0
44	Bacteroidales_[G-2] sp._oral_taxon_274	90.4	88.3	91.5	90.1
45	Selenomonas artemidis	90.3	90.4	90.4	90.1
46	Capnocytophaga leadbetteri	90.2	90.0	91.7	88.5
46	Actinomyces massiliensis	90.2	92.1	91.3	87.6
48	Fretibacterium sp._oral_taxon_360	90.0	91.2	90.4	88.7
49	Gemella morbillorum	89.8	89.5	90.3	89.4
50	Streptococcus sp._oral_taxon_074	89.3	87.0	91.0	88.3
51	Rothia aeria	89.2	90.0	91.9	85.2
52	Treponema socranskii	88.8	88.7	88.4	89.4
53	Leptotrichia hongkongensis	88.7	85.4	89.2	89.9
54	Streptococcus mutans	88.4	84.9	88.1	90.8
54	Cardiobacterium hominis	88.4	91.6	89.2	85.4
54	TM7_[G-1] sp._oral_taxon_952	88.4	90.0	90.3	85.0
57	Actinomyces gerencseriae	87.9	84.9	87.0	90.6
58	Leptotrichia wadei	87.7	86.6	87.0	89.2
59	Anaeroglobus geminatus	87.6	89.1	86.5	88.3
60	Streptococcus parasanguinis_II	87.4	87.4	84.3	91.5
61	Alloprevotella tannerae	87.3	90.0	85.6	88.0
62	Actinomyces sp._oral_taxon_171	87.2	88.7	87.4	86.2
62	Capnocytophaga sputigena	87.2	88.7	89.0	84.0
64	Fusobacterium nucleatum_subsp._nucleatum	86.8	90.8	86.5	85.0
65	Catonella morbi	86.6	87.0	87.2	85.7
66	Corynebacterium durum	85.6	88.7	86.8	82.2
66	Selenomonas infelix	85.6	88.7	85.6	83.8
68	Prevotella denticola	85.5	82.8	84.7	88.0
69	Prevotella sp._oral_taxon_317	85.2	88.3	83.6	85.4
70	Leptotrichia sp._oral_taxon_212	84.8	87.4	87.0	80.5
71	Porphyromonas sp._oral_taxon_279	84.5	83.7	85.7	83.3
72	Neisseria sicca	83.5	80.8	86.3	81.5
73	Gemella sanguinis	83.1	85.4	80.9	84.7
74	Peptostreptococcaceae_[XI][G-9] [Eubacterium]_brac	83.0	87.4	82.5	81.2
75	Tannerella forsythia	82.9	82.0	83.8	82.4
75	Neisseria flavescens	82.9	77.8	85.4	82.4
77	Prevotella sp._oral_taxon_300	82.5	80.8	81.4	85.0
78	Prevotella maculosa	82.3	80.8	81.4	84.3
79	Neisseria elongata	82.2	80.3	84.1	80.8
80	Selenomonas flueggei	82.0	79.9	82.9	81.9
81	Selenomonas sp._oral_taxon_892	81.8	85.4	83.6	77.5
82	Actinomyces israelii	81.7	79.9	80.9	83.8
83	Oribacterium sp._oral_taxon_078	81.4	78.2	80.1	84.7
84	Lachnoanaerobaculum saburreum	81.3	78.7	82.5	81.2
85	Megasphaera micronuciformis	81.1	79.1	78.5	85.4
85	Prevotella salivae	81.1	73.2	80.9	85.7
87	Streptococcus parasanguinis_I	80.6	80.3	78.2	84.0
88	Cardiobacterium valvarum	80.5	81.2	82.1	77.9
89	Prevotella oulorum	80.1	82.8	77.6	81.9
89	Veillonellaceae_[G-1] sp._oral_taxon_150	80.1	78.2	79.1	82.4
91	Leptotrichia sp._oral_taxon_417	79.7	74.5	80.3	81.7
92	Veillonella rogosae	79.6	82.4	82.7	73.9
93	Streptococcus anginosus	79.5	81.2	78.0	80.5
94	Veillonellaceae_[G-1] sp._oral_taxon_155	79.4	77.4	77.8	82.6
95	Fretibacterium fastidiosum	79.2	79.1	80.1	78.2
96	Lautropia mirabilis	78.2	82.8	78.9	74.6
97	Actinobaculum sp._oral_taxon_183	78.0	78.7	78.3	77.2
98	Fusobacterium periodonticum	77.7	74.9	82.1	73.5
99	Selenomonas sp._oral_taxon_919	77.2	84.1	75.6	75.4
100	Selenomonas sp._oral_taxon_137	76.8	80.3	76.9	74.6
101	Atopobium parvulum	76.5	76.2	73.8	80.3
101	Streptococcus lactarius	76.5	80.3	75.1	76.1
103	Kingella denitrificans	76.3	72.0	76.0	79.1
104	Capnocytophaga sp._oral_taxon_336	75.1	77.4	75.5	73.5
105	Lachnospiraceae_[G-3] sp._oral_taxon_100	73.7	77.0	73.6	72.1
106	Atopobium rimae	73.3	72.4	73.1	74.2
107	Streptococcus constellatus	73.0	72.8	71.7	74.9
108	Tannerella sp._oral_taxon_286	72.8	69.9	74.9	71.8
108	Selenomonas sp._oral_taxon_134	72.8	75.7	72.9	70.9
110	Leptotrichia buccalis	72.0	64.9	76.0	70.9
111	Selenomonas sp._oral_taxon_146	71.9	73.2	72.9	69.7
112	Leptotrichia sp._oral_taxon_215	71.8	75.3	72.4	69.0
113	Leptotrichia sp._oral_taxon_392	71.4	74.5	74.9	65.0
114	TM7_[G-5] sp._oral_taxon_356	71.2	74.5	72.2	68.1
115	Dialister pneumosintes	70.6	74.1	70.2	69.2
115	Prevotella sp._oral_taxon_472	70.6	71.5	75.3	64.1
117	Streptococcus sp._oral_taxon_056	70.6	69.0	73.3	68.1
118	Fretibacterium sp._oral_taxon_359	70.5	66.9	71.1	71.6
119	Solobacterium moorei	70.4	69.0	70.0	71.6
120	Abiotrophia defectiva	70.1	69.9	72.9	66.7
121	Lachnoanaerobaculum umeaense	69.3	69.9	70.2	67.8
122	Parvimonas sp._oral_taxon_393	69.2	69.9	70.2	67.4
123	Actinomyces meyeri	67.8	71.1	69.7	63.4
124	Centipeda periodontii	67.7	64.0	69.1	67.8
125	Leptotrichia shahii	67.6	72.4	66.6	66.2
126	Olsenella sp._oral_taxon_807	66.9	61.9	67.1	69.2
127	Actinobaculum sp._oral_taxon_848	66.3	64.9	66.1	67.4
128	Leptotrichia hofstadii	66.0	63.6	68.2	64.6
128	Neisseria oralis	66.0	66.1	69.0	62.2
128	Prevotella histicola	66.0	62.3	62.8	72.1
131	Actinomyces sp._oral_taxon_178	65.8	61.1	65.3	69.0
132	Stomatobaculum longum	65.7	62.3	63.7	70.2
132	TM7_[G-1] sp._oral_taxon_352	65.7	60.3	70.6	62.4
134	Capnocytophaga sp._oral_taxon_326	65.4	64.4	68.1	62.4
135	Prevotella oralis	64.5	63.6	65.2	64.1
136	Streptococcus sinensis	63.9	65.7	62.1	65.3
137	Alloprevotella sp._oral_taxon_308	63.5	68.6	61.9	62.7
138	Porphyromonas sp._oral_taxon_284	63.3	64.4	66.8	58.2
138	Prevotella pallens	63.3	63.2	61.7	65.5
140	Aggregatibacter sp._oral_taxon_458	63.2	65.7	65.3	58.9
141	Porphyromonas endodontalis	63.1	69.5	63.7	58.7
142	Prevotella saccharolytica	63.0	65.3	65.0	59.2
143	Selenomonas sp._oral_taxon_126	62.0	62.3	63.4	60.1
144	Lachnoanaerobaculum orale	61.1	63.2	58.5	63.4
145	Treponema maltophilum	61.0	59.0	62.3	60.3
146	Peptostreptococcaceae_[XI][G-7] [Eubacterium]_yuri	60.2	64.0	62.3	55.4
147	Veillonella denticariosi	60.1	56.5	60.8	61.3
148	Sphingomonas echinoides	59.8	64.9	61.7	54.5
149	Streptococcus australis	59.7	57.3	59.0	62.0
150	TM7_[G-1] sp._oral_taxon_348	59.6	57.7	63.4	55.9
151	TM7_[G-1] sp._oral_taxon_488	57.8	56.9	61.2	54.0
152	Ruminococcaceae_[G-1] sp._oral_taxon_075	56.9	58.2	59.7	52.6
153	Selenomonas sp._oral_taxon_936	56.8	58.2	56.3	56.8
154	Prevotella buccae	56.7	49.4	54.7	63.4
155	Leptotrichia sp._oral_taxon_225	56.4	60.3	58.7	51.4
155	Prevotella pleuritidis	56.4	55.2	59.4	53.3
157	Alloprevotella rava	56.2	57.7	56.9	54.5
158	Aggregatibacter aphrophilus	56.1	57.7	59.6	50.7
159	Treponema denticola	56.0	59.4	58.1	51.4
160	Peptostreptococcus stomatis	55.9	64.0	55.1	52.3
161	Pseudomonas fluorescens	55.5	53.6	57.0	54.5
162	Actinomyces sp._oral_taxon_170	55.2	60.3	57.0	50.0
163	Porphyromonas gingivalis	55.1	51.0	56.3	55.9
164	Haemophilus sp._oral_taxon_036	55.0	59.4	54.7	52.8
165	Johnsonella ignava	54.6	54.0	56.3	52.8
166	Porphyromonas catoniae	54.4	57.7	56.5	49.8
167	Peptostreptococcaceae_[XI][G-1] [Eubacterium]_infi	54.2	47.3	54.7	57.5
168	Prevotella sp._oral_taxon_292	53.8	49.4	54.3	55.6
169	Fretibacterium sp._oral_taxon_362	53.6	53.6	53.8	53.3
170	TM7_[G-1] sp._oral_taxon_347	53.2	58.2	54.3	49.1
171	Leptotrichia sp._oral_taxon_219	52.7	49.4	54.2	52.6
172	Aggregatibacter segnis	52.1	56.1	54.9	46.2
173	TM7_[G-3] sp._oral_taxon_351	51.9	45.6	55.4	50.9
174	Fusobacterium sp._oral_taxon_370	51.1	45.6	55.6	48.4
175	Prevotella sp._oral_taxon_313	50.5	53.1	46.4	54.2
176	Prevotella loescheii	50.4	52.3	54.5	43.9
177	Desulfobulbus sp._oral_taxon_041	50.3	48.1	50.9	50.7
178	Capnocytophaga sp._oral_taxon_864	49.1	51.9	52.0	43.9
179	Ottowia sp._oral_taxon_894	48.4	49.4	50.2	45.5
180	Microbacterium flavescens	48.0	50.2	49.5	44.8
181	Leptotrichia sp._oral_taxon_498	47.8	42.3	48.2	50.5
182	Granulicatella elegans	47.3	52.7	45.5	46.5
183	Filifactor alocis	46.8	50.2	46.8	44.8
184	Campylobacter curvus	46.2	45.6	46.4	46.2
185	Mycoplasma salivarium	45.0	47.7	45.3	43.2
186	Capnocytophaga sp._oral_taxon_338	44.8	42.7	44.4	46.5
187	Capnocytophaga sp._oral_taxon_902	44.3	43.1	46.6	42.0
188	Treponema sp._oral_taxon_231	43.6	51.5	45.3	37.1
189	Tannerella sp._oral_taxon_808	43.1	39.3	45.7	41.8
190	Prevotella intermedia	42.8	40.2	43.3	43.7
191	TM7_[G-6] sp._oral_taxon_870	42.7	43.9	44.2	39.9
192	Mitsuokella sp._oral_taxon_131	42.2	35.6	41.0	47.7
193	Haemophilus haemolyticus	42.0	47.3	41.3	39.9
194	Prevotella veroralis	41.7	44.4	40.8	41.3
195	Veillonellaceae_[G-1] sp._oral_taxon_129	40.9	37.7	40.8	43.0
195	Aggregatibacter paraphrophilus	40.9	45.2	42.2	36.6
197	Pseudoramibacter alactolyticus	40.9	33.5	41.5	44.1
198	Bergeyella sp._oral_taxon_907	40.6	39.7	42.8	38.3
199	Bifidobacterium dentium	40.5	33.9	37.9	47.7
199	TM7_[G-1] sp._oral_taxon_869	40.5	41.8	41.5	38.5
201	Prevotella sp._oral_taxon_306	40.3	36.0	39.5	43.7
202	Prevotella dentalis	39.3	40.2	36.5	42.5
203	Shuttleworthia satelles	39.2	34.7	37.5	43.9
204	Veillonella sp._oral_taxon_780	39.1	40.6	42.4	34.0
205	Capnocytophaga sp._oral_taxon_412	38.7	38.9	41.5	35.0
206	Neisseria subflava	38.6	39.3	37.4	39.7
206	Scardovia wiggsiae	38.6	37.2	37.7	40.4
208	Leptotrichia sp._oral_taxon_223	37.8	42.3	36.5	37.1
209	Peptostreptococcaceae_[XI][G-6] [Eubacterium]_noda	37.2	35.6	37.0	38.5
209	Selenomonas sp._oral_taxon_937	37.2	36.4	35.2	40.1
211	Bacteroidetes_[G-5] sp._oral_taxon_511	36.8	41.0	37.2	33.8
212	Capnocytophaga sp._oral_taxon_323	36.7	36.0	37.4	36.2
213	Selenomonas sp._oral_taxon_149	36.4	30.5	33.8	43.2
213	Selenomonas sp._oral_taxon_442	36.4	30.1	37.5	38.5
215	SR1_[G-1] sp._oral_taxon_874	36.3	39.3	40.3	29.6
216	Porphyromonas sp._oral_taxon_275	36.0	36.4	37.7	33.6
217	Selenomonas sp._oral_taxon_478	35.9	31.4	38.4	35.2
218	Alloprevotella sp._oral_taxon_473	35.8	39.7	36.8	32.4
219	Neisseria bacilliformis	35.5	35.1	35.4	35.9
220	Capnocytophaga sp._oral_taxon_324	35.4	32.6	38.1	33.3
221	Veillonellaceae_[G-1] sp._oral_taxon_145	35.2	35.1	36.1	34.0
222	Atopobium sp._oral_taxon_199	34.9	36.0	36.1	32.6
223	Lachnospiraceae_[G-8] sp._oral_taxon_500	34.8	33.1	35.7	34.5
224	TM7_[G-2] sp._oral_taxon_350	34.6	34.7	34.7	34.5
225	Bacteroidaceae_[G-1] sp._oral_taxon_272	33.1	26.8	33.9	35.7
225	Capnocytophaga sp._oral_taxon_903	33.1	31.8	36.1	30.0
225	Selenomonas dianae	33.1	28.9	35.0	32.9
228	Prevotella baroniae	33.0	32.2	33.4	32.9
229	Leptotrichia goodfellowii	32.2	37.2	34.1	27.0
230	Prevotella micans	31.4	29.3	32.7	31.0
231	Porphyromonas sp._oral_taxon_278	31.0	31.8	31.9	29.3
232	Prevotella sp._oral_taxon_314	30.8	30.1	31.4	30.5
233	Haemophilus parahaemolyticus	30.3	37.2	31.8	24.4
234	Anaerolineae_[G-1] sp._oral_taxon_439	30.2	25.5	30.0	33.1
235	Selenomonas sp._oral_taxon_133	30.1	28.9	30.5	30.3
236	Treponema lecithinolyticum	29.5	30.1	31.2	26.8
237	Peptostreptococcaceae_[XI][G-5] [Eubacterium]_saph	28.3	22.2	31.4	27.7
238	Treponema sp._oral_taxon_237	27.0	32.2	28.9	21.6
239	Neisseria pharyngis	25.8	25.1	25.5	26.8
240	Leptotrichia sp._oral_taxon_879	25.4	25.9	25.6	24.9
241	Brevundimonas diminuta	25.3	27.6	25.8	23.2
242	Bradyrhizobium elkanii	24.9	26.4	22.7	26.8
242	Capnocytophaga sp._oral_taxon_332	24.9	29.7	26.2	20.4
244	Megasphaera sp._oral_taxon_123	24.5	32.2	24.2	20.7
245	Capnocytophaga sp._oral_taxon_380	22.2	20.5	24.9	19.7
246	Treponema vincentii	22.0	23.0	23.5	19.5
247	Prevotella sp._oral_taxon_376	21.4	26.8	23.5	15.7
248	Prevotella sp._oral_taxon_526	21.1	21.8	21.7	20.0
249	Aggregatibacter sp._oral_taxon_513	20.2	21.8	23.1	15.5
250	Lactobacillus gasseri	19.9	18.0	17.9	23.7
251	Fretibacterium sp._oral_taxon_358	19.3	16.7	19.3	20.7
252	Mitsuokella sp._oral_taxon_521	18.7	18.4	19.9	17.4
253	Fretibacterium sp._oral_taxon_361	18.5	15.5	19.1	19.2
254	Prevotella sp._oral_taxon_475	18.2	19.2	20.0	15.3
255	Treponema medium	18.1	16.3	19.5	17.4
256	Johnsonella sp._oral_taxon_166	16.7	12.6	18.6	16.7
257	Streptococcus sobrinus	15.9	13.8	14.4	19.0
258	Prevotella multiformis	15.5	16.3	15.5	15.0
259	Butyrivibrio sp._oral_taxon_080	14.5	15.1	15.9	12.4
260	GN02_[G-2] sp._oral_taxon_873	13.7	10.9	15.0	13.6
261	Aggregatibacter actinomycetemcomitans	12.3	12.1	11.4	13.6
262	Leptothrix sp._oral_taxon_025	10.9	13.4	11.4	8.9
263	Atopobium sp._oral_taxon_416	8.7	5.4	8.5	10.8
264	Sphingomonas sp._oral_taxon_006	7.2	5.9	7.9	7.0
265	Porphyrobacter tepidarius	6.9	7.1	6.1	7.7
266	Treponema sp._oral_taxon_247	4.8	6.3	4.5	4.2
267	Pyramidobacter piscolens	4.0	2.9	3.8	4.9

^a^Bacteria in the table are rank ordered according to their prevalence in the overall cohort

Dashed line inserted below top 20 taxa

## Discussion

The objective of the present study was to characterize, using high throughput sequencing of the 16S rRNA bacterial gene, the subgingival microbiome in relation to age among community-dwelling postmenopausal women, aged 53–81 years, whose selection into the study was not conditioned on presence or severity of periodontitis. We identified 267 taxa, of which 55% had previously been named within the HOMD database. The remaining previously unnamed OTUs could potentially identify novel microbiota residing in human subgingival biofilm, new discovery that could have important implications to periodontal microbiology [17, 34, 35]. The majority of taxa identified in our study fell within the four major human bacterial phyla (*Actinobacteria, Bacteroidetes, Firmicutes,* and *Proteobacteria*) determined in the HMP [36] and by others [3, 23] including the oral microbiome [21, 22, 37–39]. As in other studies on the oral microbiome [21, 22, 38–41], the most abundant phyla in our study were *Firmicutes*, *Bacteroidetes*, *Fusobacteria* and *Proteobacteria,* accounting for 46, 17, 14, and 9%, respectively, of the 265 taxa identified. The *Firmicutes-to-Bacteroidetes* ratio has been suggested as a possible indicator of the overall status of a microbial habitat in aging humans [6]. Previous studies on the gut microbiome have shown a lower ratio in older compared with younger individuals [42, 43]. In contrast, we observed a tendency toward higher *Firmicutes-to-Bacteroidetes* ratios across incremental age groups. In so much as some of the most virulent and well established periodontal pathogens (e.g., *P. gingivalis, T. forsythia, T. denticola*) reside in the phylum *Bacteroides*, whereas bacteria associated with a healthy periodontium (*S. sanguis, oralis*) reside in the phylum *Firmicutes*, a higher *F-B* ratio in the present cohort of aging women might be expected given the relatively small mean probing pocket depth (2.2 mm) overall, and lack of difference in this clinical measure of periodontitis across age groups. Whether the *F-B* ratio has similar relevance in the oral microbiome as has been reported previously for the gut microbiome requires further investigation.

The most abundant genus in our cohort was *Veillonella*, followed by *Streptococcus*, *Fusobacterium*, and *Prevotella*, with little variation in the distribution across age categories. Previous studies using targeted methods for measuring oral microbiota found substantially elevated abundance of *Actinomyces* and *Fusobacterium* genera in older adults [17, 38], which was not the case in our study (*Actinomyces, overall: 1%, 70–79 years: 2%; Fusobacterium, overall: 11%, 70–79 years 10%)* when using untargeted high-throughput sequencing. Other studies that measured the oral microbiome using 16S rRNA sequencing have reported the distribution of genera. Among community-dwelling adults (mean age 83; 61% women), analysis of salivary microbiome revealed *Prevotella* (22%) was most abundant, followed by *Neisseria* (12%), *Veillonella* (10%), and *Streptococcus* (8%) [22]. In another study on the salivary microbiome in Mexican American women, aged 50 and older, Hoffman et al. [21] reported that *Streptococcus* was most abundant (37%), followed by *Prevotella* (11%), *Haemophilus* (10%), and *Veillonella* (6%). Among Alaskan adults, aged 20–40 years, *Streptococcus* (28%) and *Prevotella* (27%) were by far most abundant, followed by *Rothia* (11%) and *Veillonella* (8%) [41]. Variation of microbial genera with age was not reported in these previous studies. Notwithstanding, there does appear to be some consistency across studies using culture-independent sequencing methods, including ours, in that *Streptococcus, Prevotella,* and *Veillonella* are abundant microbial genera commonly observed in the adult human oral microbiome.

Our primary analysis on microbial species composition and variation with age was based on CLR transformed OTUs taking into account the complex compositional structure of microbiome data [32]. The top 20 most abundant bacterial species had CLR means from 3.85 to 8.25, indicating these species were 14- to 304-fold (i.e., 2^3.85^ to 2^8.25^) more abundant than the overall composition mean (Table 3). *V. dispar, S. oralis,* and *V. parvula* were the top three most abundant species, each with CLR means > 7. *V. dispar* and *parvula* are gram-negative anaerobic bacteria commonly found in the human oral cavity [44], and have been associated with caries and periodontitis [34]. Evidence suggests *V. parvula* synergizes with *Lachnoanaerobaculum (Eubacterium) saburreum,* and the energy it produces, as a critical part of human subgingival biofilm formation [45]. *L. saburreum* was found at a relatively small, but elevated, abundance in our cohort (mean CLR, 1.07). Both bacterial species were positively correlated with age in our study, with a stronger correlation for *V. parvula* (*r* = 0.10) compared with *L. saburreum* (*r* = 0.04). *S. oralis*, in contrast, tends to be abundant in soft tissues of healthy periodontium [34], and as such was an original component in Socransky’s “*yellow complex*” defined using the checkerboard DNA-DNA hybridization method. *S. oralis* abundance has been shown to decline in the setting of experimental subgingival biofilm growth [34], which suggests it might be a key bacterium involved with the *shift* from a healthy to disease subgingival microbial ecology leading to periodontitis. The correlations with age for *V. parvula* (*r* = 0.10) and *S. oralis* (*r* = − 0.10) observed in the present study suggest that age could be a potential host factor contributing to susceptibility for untoward shifts in the human subgingival microbial ecology. Chronological age, per se, however, may not be the biologically relevant effector of shifts in microbial ecology. Rather, the tendency of aging to be associated with chronic immune function decline and upregulated proinflammatory signaling [8], referred to as “inflamm-aging” by Franceshi and coworkers [46] is likely a culpable perturbation of colonizing microbiota. Consistent with this hypothesis are results from studies of experimentally induced gingivitis, which demonstrated markedly greater amounts and severity of biofilm development in older than younger adults, despite no obvious differences in microbial compositional characteristics of the biofilm between age groups [17].

In the present study, 12 bacterial species differed significantly across age groups (Fig. 5). The largest difference in bacteria elevated in older adults was for *B. dentium* (phylum *Actinobacteria*), an anaerobe that has strong adhesion capacity, tolerates highly acidic conditions, and has been associated with human dental caries [47], but also with suppression of *P. gingivalis*, a virulent periodontitis pathogen [48]. This might partially explain why *P. gingivalis* was in relatively low abundance in our cohort of older women. *Anaeroglobus geminatus* (phylum *Firmicutes*) *also* demonstrated a rather large elevation in older compared with younger adults in our cohort. This bacterium has an identified role in perturbing a shift in the subgingival microbial ecology that favors development of periodontitis [49]. There was no difference in mean pocket depth measures among age groups in our cohort of older women, among whom prevalence of major risk factors for periodontitis, smoking and diabetes, also were low. However, it is conceivable that higher abundance of *B. dentium* and *A. geminatus* in the older age group could be reflective of an ongoing subgingival microbial community shift that leads to increased susceptibility to periodontitis progression in these women over time. Longitudinal analyses are required to confirm this hypothesis.

*S. sanguinis and Corynebacterium durum* showed the largest differences in bacteria between age groups among those elevated in younger women (Fig. 5). *S. sanguinis* (phylum *Firmicutes*) is a gram-positive anaerobe that is abundant in healthy periodontium [34] and plays a role in modifying the environment on oral surfaces such as to suppress growth of other *Streptococci* bacteria involved with oral diseases, such as *S. mutans* which is a causal agent in human carries [50]. *S. sanguinis* also might play a role in the shift of subgingival microbiota from a healthy to a disease ecology, serving as an adhesion site for virulent periodontal pathogens, such as *P. gingivalis and F. nucleatum* [50], each of which were in relatively low abundance in the present study. The role that *C. durum* (phylum *Actinobacteria*), also a gram-positive bacterium, might have in the subginigival microbial ecology is not entirely clear. Elevations of this bacterium originally was identified in bronchial wash solution and implicated in maintaining a healthy respiratory tract [51] and later, it’s reduction in saliva was associated with halitosis [52] and celiac disease [53]. Given it’s propensity to produce acid from available sugar compounds in saliva [54], and perhaps in other oral fluids including the gingival crevice, it is possible that this bacterium has a role in establishing or maintaining pH of the gingival pocket at a level commensurate with survival of other bacteria associated with periodontal health, such as *S. sanguinis*.

The vast majority of studies using untargeted high-throughput sequencing methods of the oral microbiome have reported measures of relative abundance or prevalence when describing microbial composition. Our primary measure for analysis of microbiota abundance in was the centered log-transformed ratio (CLR) OTU, as recommended by Gloor and coworkers [32]. While the basic cross-sectional findings of the present study were generally consistent when based on mean CLR OTUs, relative abundance, and prevalence, we believe that the CLR approach is the method of choice. Compositional data are vectors of non-negative numbers that sum to a fixed value, a constraint that can lead to spurious correlations. Subsequent work by Aitchison and colleagues yielded a set of log-ratio transformations that alleviate the sum-constraint burden, provide a consistent variance-covariance structure, and ensure that statistical results show consistency over subcompositions and OTU permutations [32, 55]. Subcompositional consistency, in particular, is necessary for the fundamental scientific concept of reproducibility across studies. The application of methods which ignore the compositional structure of microbiome data, like simple proportions (e.g., relative abundance, prevalent, or rarefaction) can lead to false positive associations and inferences [32]. In addition, the CLR transformation does not reduce the dimensionality of the dataset, maintaining the correspondence between transformed variables and OTUs, and easing the interpretation of conventional statistical tests, such as bivariate correlations and analysis of variance. Given the recent growth in microbiome research, the plethora of published studies that used different analytic methods, and the potential impact that continued investigation of the human microbiome could have on future understanding of disease etiology and therapeutics [24], the need for standardization of methods for analyzing and reporting microbiome data is paramount.

The present study has both strengths and limitations that need be considered when interpreting and generalizing its findings. Strengths include the large sample size of community-dwelling older postmenopausal women whose selection into the study was not conditioned on periodontitis presence or severity. Of the published studies reporting on the oral microbiome in older adults, the vast majority included relatively small sample sizes (e.g., < 100) and individuals that were selected to have either periodontal health or disease, often recruited from dental or other healthcare settings [17, 18, 22, 37, 40, 56]. Understanding the epidemiology of oral microbiota composition and its association with host characteristics in a more general community setting is a critical foundational step for evaluating associations between oral microbiome and both oral and systemic disease, as well as response to therapeutic intervention [24]. Previous oral microbiome studies on older adults relied largely on targeted low-throughput methods for characterizing oral microbiota [17, 18, 38, 56]. The limitations of these methods have been discussed elsewhere [17, 57]. Only recently have studies, including ours, utilized state-of-the science untargeted high-throughput next generation sequencing methods to investigate the oral microbiome in adults in middle- and older ages [21–23, 39–41]. This not only allows for greater sensitivity in characterizing the complexity of oral microbial communities, but also for potential discovery of new previously unidentified microbiota, which is essential to deeper understanding of the oral microbiome [17, 35]. Weaknesses of the present study include its cross-sectional design, which precludes temporal understanding of the relationship between aging and formation of the observed oral microbiome. The cross-sectional nature of our results precludes causal inferences regarding the relationship between age and the subgingival microbial composition and diversity. Using means to describe complex data, such as the subgingival microbiome, is helpful for descriptive purposes and ease of understanding, however they do not provide insight on between-subject variability nor do they allow for understanding of shifts between healthy and disease ecologies [34]. Prospective studies are needed using statistical methods appropriate for quantifying changes in microbiota between groups differing on host characteristics, such as aging or periodontal disease onset and progression, or in response to therapeutic intervention. The present cross-sectional observations, such as the significant differences in CLR mean OTUs between older and younger women (Fig. 5), could inform development of hypotheses for testing in a prospective study design. Lastly, we were not able to determine the functional attributes of the particularly abundant or sparse microbiota identified in our older cohort of women, nor of the bacterium that differed in abundance between older and younger women. It is becoming clearer that the functions determined by the genes expressed by microbiota are likely more influential on health or disease states than is the microbial composition [1, 15, 17]. Because aging is a non-modifiable host characteristic intimately involved with both structural and functional changes in the human body over the adult lifespan, the relationship between age and microbial function is of high interest [7].

## Conclusion

We conclude that in a large cross-sectional analysis on the subgingival microbiome in postmenopausal women, aged 53–81 years, who were not selected on the basis of periodontitis status, a diverse subgingival microbiome was present and several bacterial species were correlated with age across the age range studied. Twelve microbiota were identified that differed significantly in abundance between women aged 50–59 versus 70 and older. Prospective data are needed to characterize the temporal relation between aging and shifts or stability in the abundance and pattern of subgingival microbiota observed herein to better elucidate the role, if any, that aging has on the oral microbiome. Age alone, however, does not determine the human subgingival microbiome. Other factors, including senescence of tissues and functions, side effects of medication use, status of the gingiva and dentition, systemic diseases, oral hygiene and behavioral habits, are thought to influence the microbiome. The extensive cross-sectional observations reported here provide a starting point and direction to define a targeted subset of bacteria that appear be related with age for further analysis in which issues such as confounding or interaction with the above and other factors can be evaluated with greater statistical efficiency involving fewer tests to correct for false discovery. This will be the focus of a forthcoming manuscript from our longitudinal cohort. Additional understanding about the functions of bacteria that differ with age in later life could identify intervention targets for enhanced oral health and, possibly control of other diseases.

## Data Availability

Data that support the findings of this study are available from the authors upon reasonable request and with permission of the U.S. Women’s Health Initiative program.

## Abbreviations

CLR: Centered-log(2)-ratio
OTU: Operational Taxonomic Unit
